# Supplementary material for: Nuclear phosphoinositide signaling promotes YAP/TAZ-TEAD transcriptional activity in breast cancer
Source: EMBO J. 2024 Apr 2;43(9):4. doi: 10.1038/s44318-024-00085-6 (PMC11066040; doi:10.1038/s44318-024-00085-6)
Supplement: Supplementary file 1 — Appendix [file 44318_2024_85_MOESM1_ESM.pdf]

Appendix for

Nuclear phosphoinositide signaling promotes YAP/TAZ-TEAD transcriptional activity in breast cancer

Table of contents:

Appendix Figure S1………………………………………………………….………..2-4

GST-YAP binds to $PI4,5P_2$ and $PI3,4,5P_3$ with submicromolar dissociation constants..5

Appendix Figure S2………………………………………………………….………..6-7

The phosphoinositide binding of YAP controls MDA-MB-231 cell migration……………8

Appendix Figure S3………………………………………………………….…………..9

PIPKIα and IPKM are overexpressed in breast cancer tissues………………………...10

Binding check (5 nM GST or GST-YAP and 5 μM PI, $PI4,5P_2$, or $PI3,4,5P_3$)

Signal to Noise Ratio: **GST and PI**

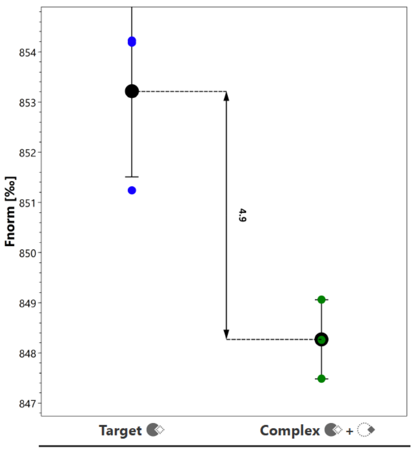

Response Evaluation:	On Time 20s	
Response:	853	848
Noise:	1.7	0.8
Response Amplitude:	4.9	
Signal to Noise Ratio:	4.0 !	

Signal to Noise Ratio: **GST-YAP and PI**

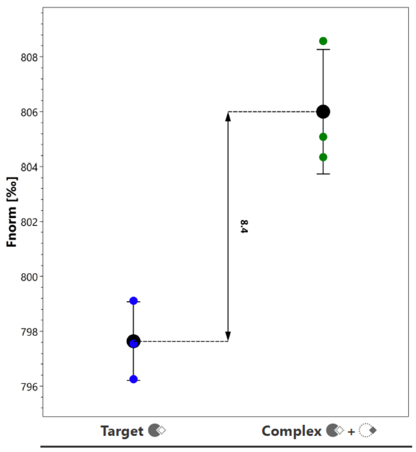

Response Evaluation:	On Time 20s	
Response:	798	806
Noise:	1.4	2.3
Response Amplitude:	8.4	
Signal to Noise Ratio:	4.5 !	

Signal to Noise Ratio: **GST and $PI4,5P_2$**

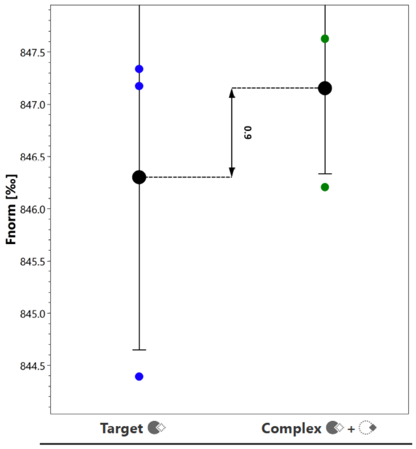

Response Evaluation:	On Time 20s	
Response:	846	847
Noise:	1.7	0.8
Response Amplitude:	0.9	
Signal to Noise Ratio:	0.7 !	

Signal to Noise Ratio: **GST-YAP and $PI4,5P_2$**

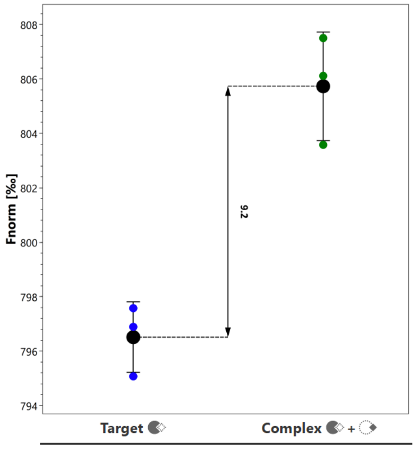

Response Evaluation:	On Time 20s	
Response:	797	806
Noise:	1.3	2.0
Response Amplitude:	9.2	
Signal to Noise Ratio:	5.6 ✓	

Signal to Noise Ratio: **GST and $PI3,4,5P_3$**

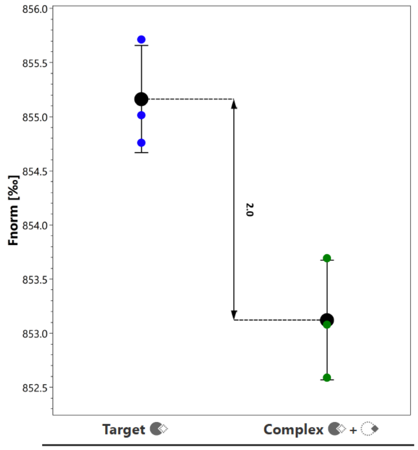

Response Evaluation:	On Time 10s	
Response:	855	853
Noise:	0.5	0.6
Response Amplitude:	2.0	
Signal to Noise Ratio:	3.9 !	

Signal to Noise Ratio: **GST-YAP and $PI3,4,5P_3$**

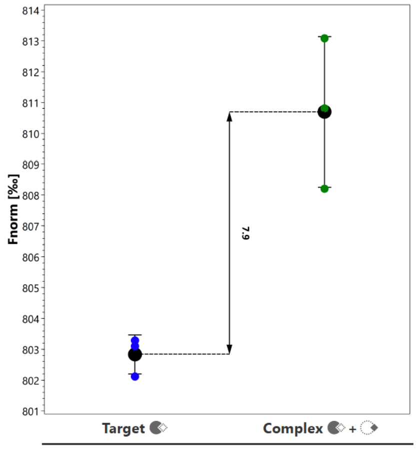

Response Evaluation:	On Time 10s	
Response:	803	811
Noise:	0.6	2.4
Response Amplitude:	7.9	
Signal to Noise Ratio:	5.1 ✓	

Appendix Figure S1

GST-YAP and 18:0/20:4 PI4,5P$_2$ binding

Repeat #1

Dose Response

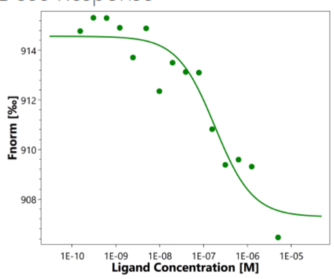

Response Evaluation: On Time 2.5s

Kd model

**Unbound**	914.6
**Bound**	907.3
**Kd**	179 nM
**TargetConc**	5 nM

Response Amplitude: 7.3
Noise: 0.8
Signal to Noise Ratio: 8.8

Capillary Scans

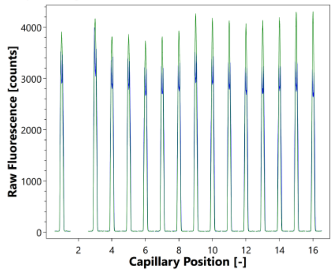

Initial Fluorescence:
Average: 4029 counts
Variation: ±7.3 %

No adsorption

No Ligand Induced Fluorescence Change

MST Traces

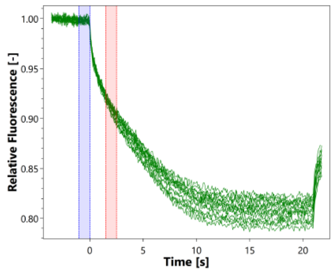

Cursor positions:
Cold Region: -1s - 0s
Hot Region: 1.5s - 2.5s

No Aggregation

No Ligand Induced Photobleaching Rate Change

Repeat #2

Dose Response

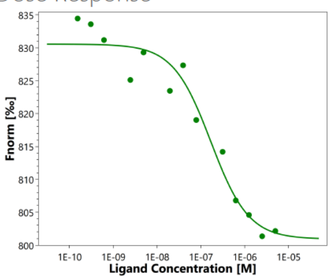

Response Evaluation: On Time 15s

Kd model

**Unbound**	830.6
**Bound**	800.9
**Kd**	166 nM
**TargetConc**	5 nM

Response Amplitude: 29.6
Noise: 2.7
Signal to Noise Ratio: 10.9

Capillary Scans

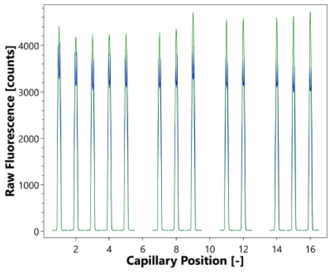

Initial Fluorescence:
Average: 4401 counts
Variation: ±6.8 %

No adsorption

No Ligand Induced Fluorescence Change

MST Traces

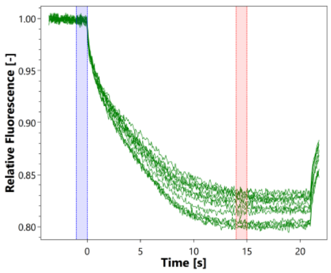

Cursor positions:
Cold Region: -1s - 0s
Hot Region: 14s - 15s

No Aggregation

No Ligand Induced Photobleaching Rate Change

Repeat #3

Dose Response

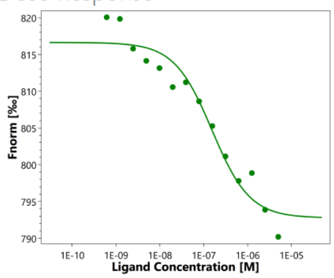

Response Evaluation: On Time 20s

Kd model

**Unbound**	816.6
**Bound**	792.8
**Kd**	152 nM
**TargetConc**	5 nM

Response Amplitude: 23.9
Noise: 2.3
Signal to Noise Ratio: 10.5

Capillary Scans

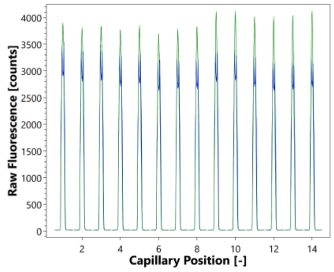

Initial Fluorescence:
Average: 3879 counts
Variation: ±6.3 %

No adsorption

No Ligand Induced Fluorescence Change

MST Traces

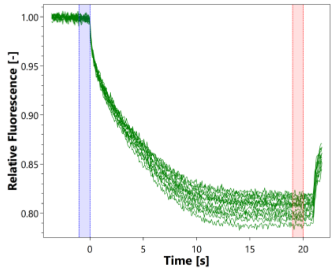

Cursor positions:
Cold Region: -1s - 0s
Hot Region: 19s - 20s

No Aggregation

No Ligand Induced Photobleaching Rate Change

	K_d (nM)
Repeat #1	179.00
Repeat #2	166.00
Repeat #3	152.00
Avarage	165.67
STDEV	13.50

Appendix Figure S1 (continued)

GST-YAP and 18:0/20:4 PI3,4,5P_3 binding

Dose Response

Repeat #1

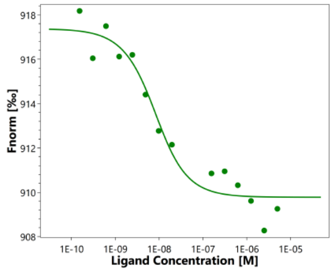

Response Evaluation: On Time 2.5s

Kd model

**Unbound**	917.4
**Bound**	909.8
**Kd**	5.66 nM
**TargetConc**	5 nM

Response Amplitude: 7.6
Noise: 0.8
Signal to Noise Ratio: 9.9

Capillary Scans

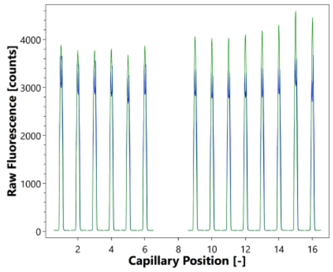

Initial Fluorescence:
Average: 3998 counts
Variation: ±14.7 %

No adsorption

No Ligand Induced Fluorescence Change

MST Traces

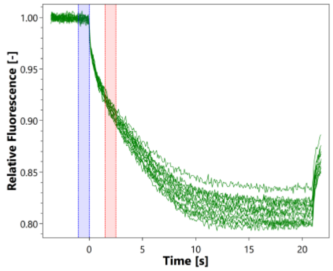

Cursor positions:
Cold Region: -1s - 0s
Hot Region: 1.5s - 2.5s

No Aggregation

No Ligand Induced Photobleaching Rate Change

Dose Response

Repeat #2

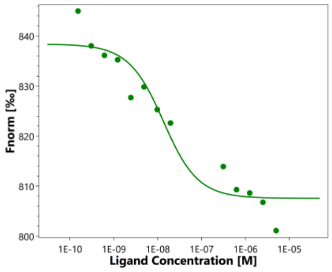

Response Evaluation: On Time 10s

Kd model

**Unbound**	838.4
**Bound**	807.6
**Kd**	10.9 nM
**TargetConc**	5 nM

Response Amplitude: 30.8
Noise: 3.8
Signal to Noise Ratio: 8.1

Capillary Scans

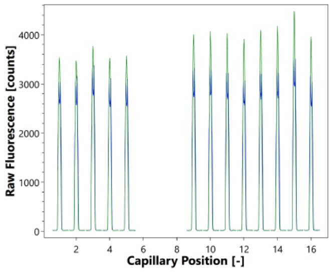

Initial Fluorescence:
Average: 3854 counts
Variation: ±15.0 %

No adsorption

No Ligand Induced Fluorescence Change

MST Traces

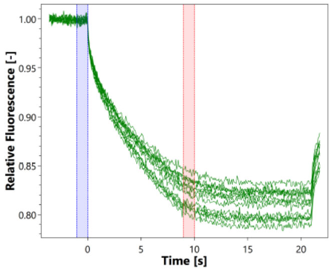

Cursor positions:
Cold Region: -1s - 0s
Hot Region: 9s - 10s

No Aggregation

No Ligand Induced Photobleaching Rate Change

Dose Response

Repeat #3

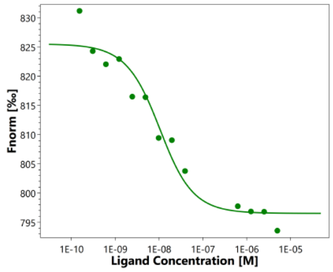

Response Evaluation: On Time 15s

Kd model

**Unbound**	825.5
**Bound**	796.5
**Kd**	7.83 nM
**TargetConc**	5 nM

Response Amplitude: 29.0
Noise: 2.7
Signal to Noise Ratio: 10.9

Capillary Scans

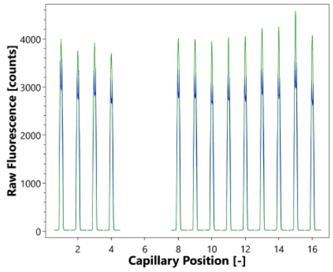

Initial Fluorescence:
Average: 3986 counts
Variation: ±13.8 %

No adsorption

No Ligand Induced Fluorescence Change

MST Traces

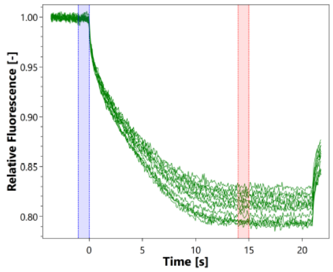

Cursor positions:
Cold Region: -1s - 0s
Hot Region: 14s - 15s

No Aggregation

No Ligand Induced Photobleaching Rate Change

	K_d (nM)
Repeat #1	5.66
Repeat #2	10.90
Repeat #3	7.83
Avarage	8.13
STDEV	2.63

Appendix Figure S1 (continued)

Appendix Figure S1. GST-YAP binds to PI4,5P_2 and PI3,4,5P_3 with submicromolar dissociation constants.

The quality of the indicated binding was monitored by incubating 5 nM fluorescently labelled GST proteins with 5 μM non-labelled lipids in triplicate. A signal-to-noise ratio must be above 5.0 for binding to be reproducibly measured in the MST instrument that was used. The binding of GST-YAP to PI4,5P_2 and PI3,4,5P_3 passed the quality check, while the binding to GST was not significant. We further measured the binding affinities between GST-YAP and PI4,5P_2 and PI3,4,5P_3 by incubating a constant concentration of fluorescently labelled GST or GST-YAP (target, 5 nM) with increasing concentrations of non-labelled 18:0/20:4 PI4,5P_2 or PI3,4,5P_3 (Fig. EV3E) to calculate a dissociation constant (K_d). The interaction of GST-YAP with PI4,5P_2 and PI3,4,5P_3 showed sigmoidal curves, and the K_ds were measured to be 165.67±13.50 and 8.13±2.63 nM, respectively.

Appendix Figure S2

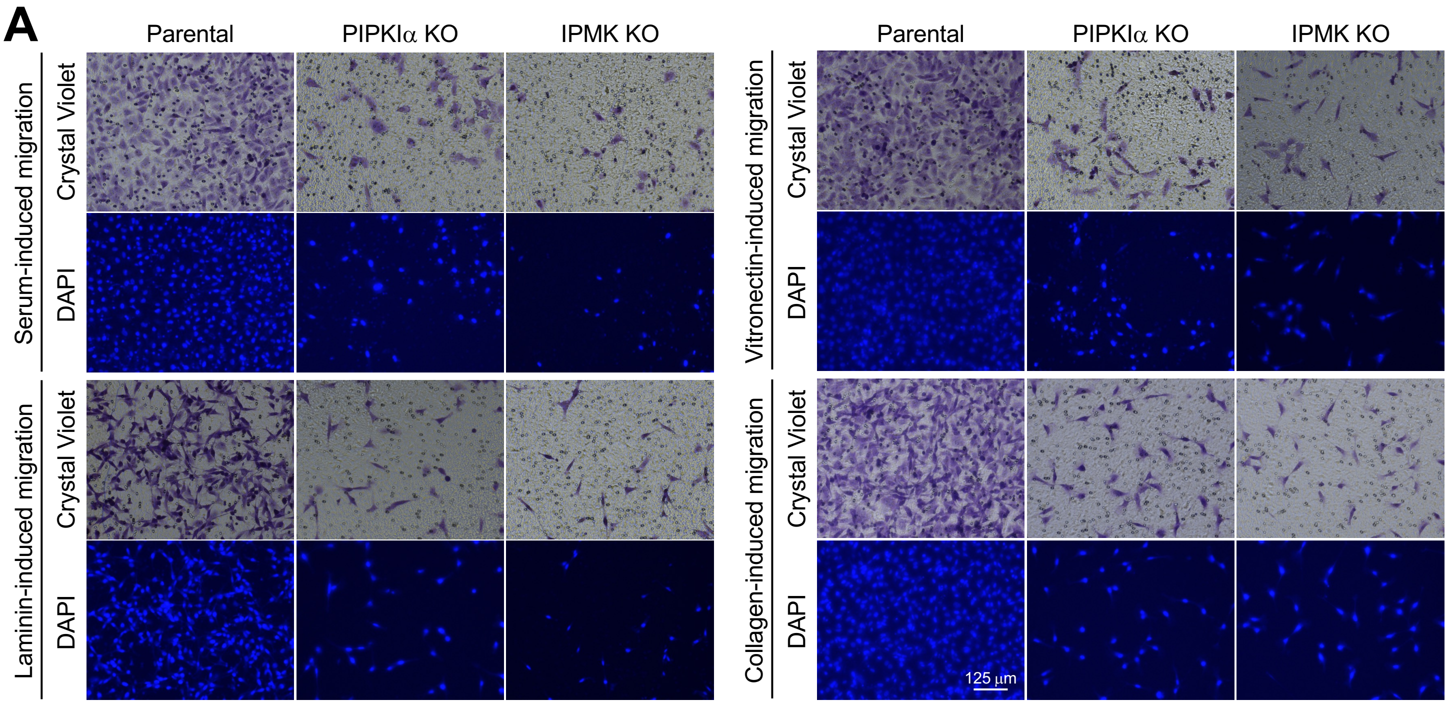

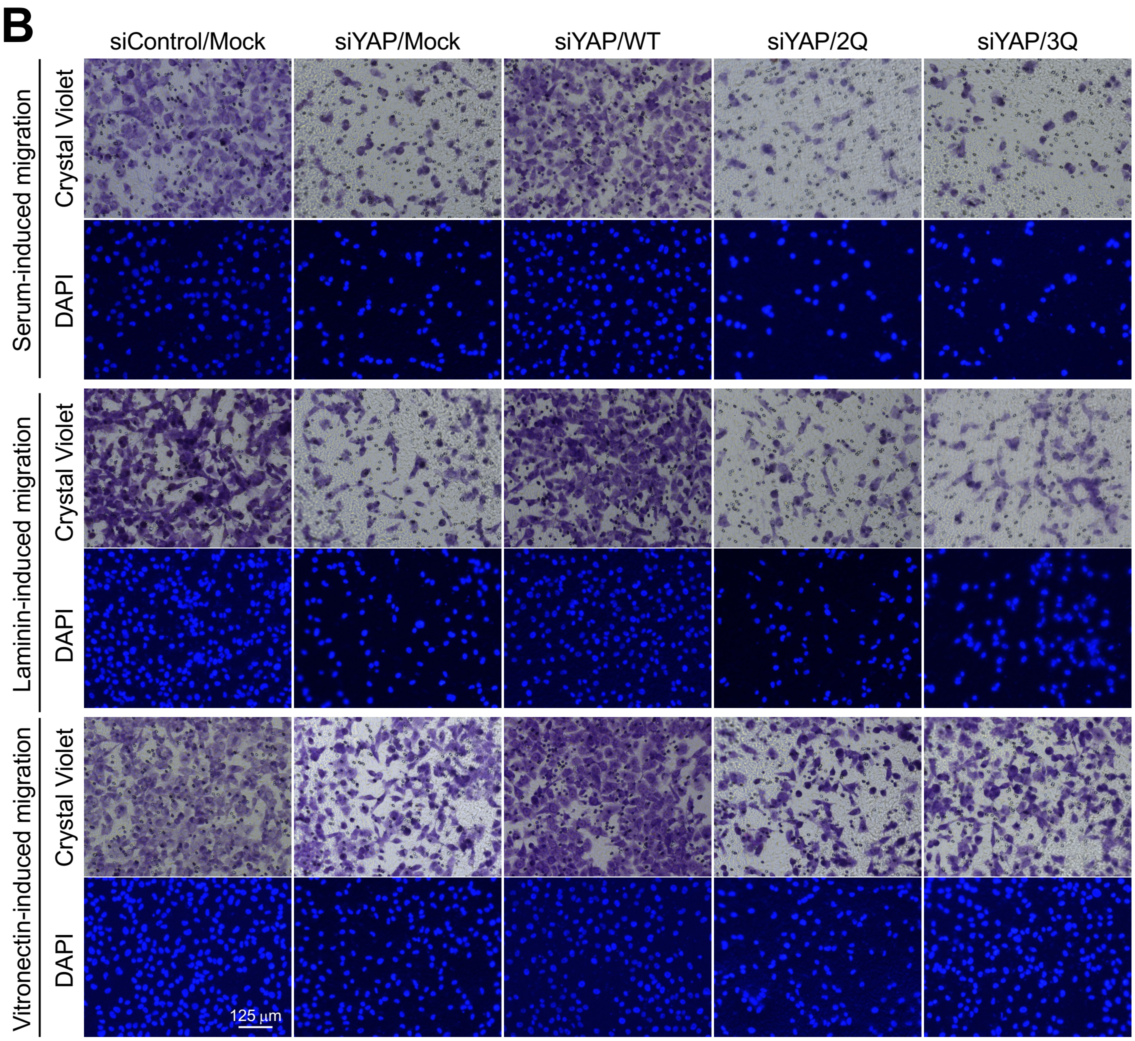

Appendix Figure S2 (continued)

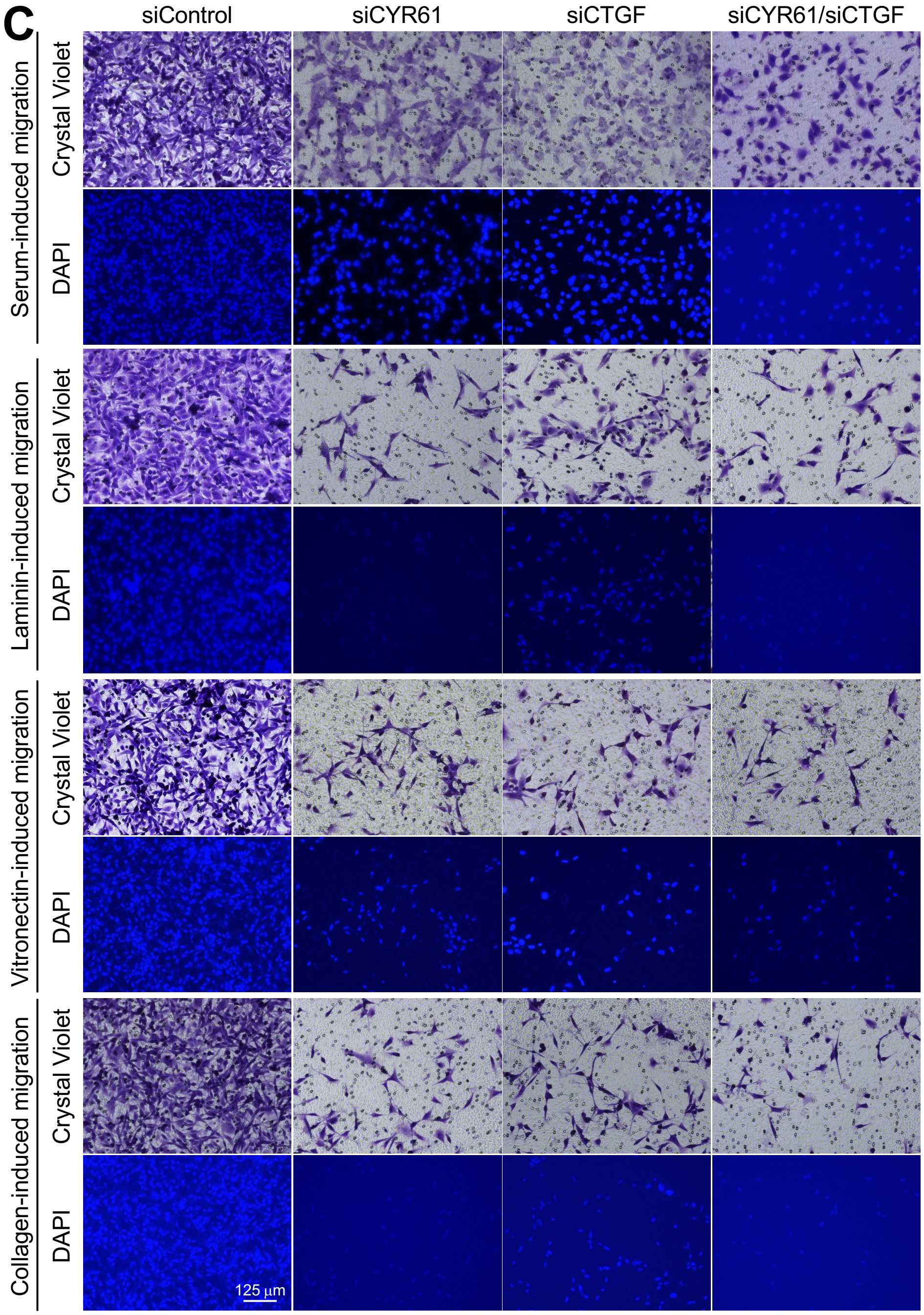

Appendix Figure S2. The phosphoinositide binding of YAP controls MDA-MB-231 cell migration.

A-C. Chemotaxis and haptotaxis of control, knock down and rescued cells were measured using Transwell assays. Cells on the bottom of the Transwell inserts were fixed and stained with 0.1% crystal violet and DAPI and then imaged using widefield epifluorescence microscopy. Representative images of *n*=3 independent experiments are shown. Scale bar, 125 μm.

Appendix Figure S3

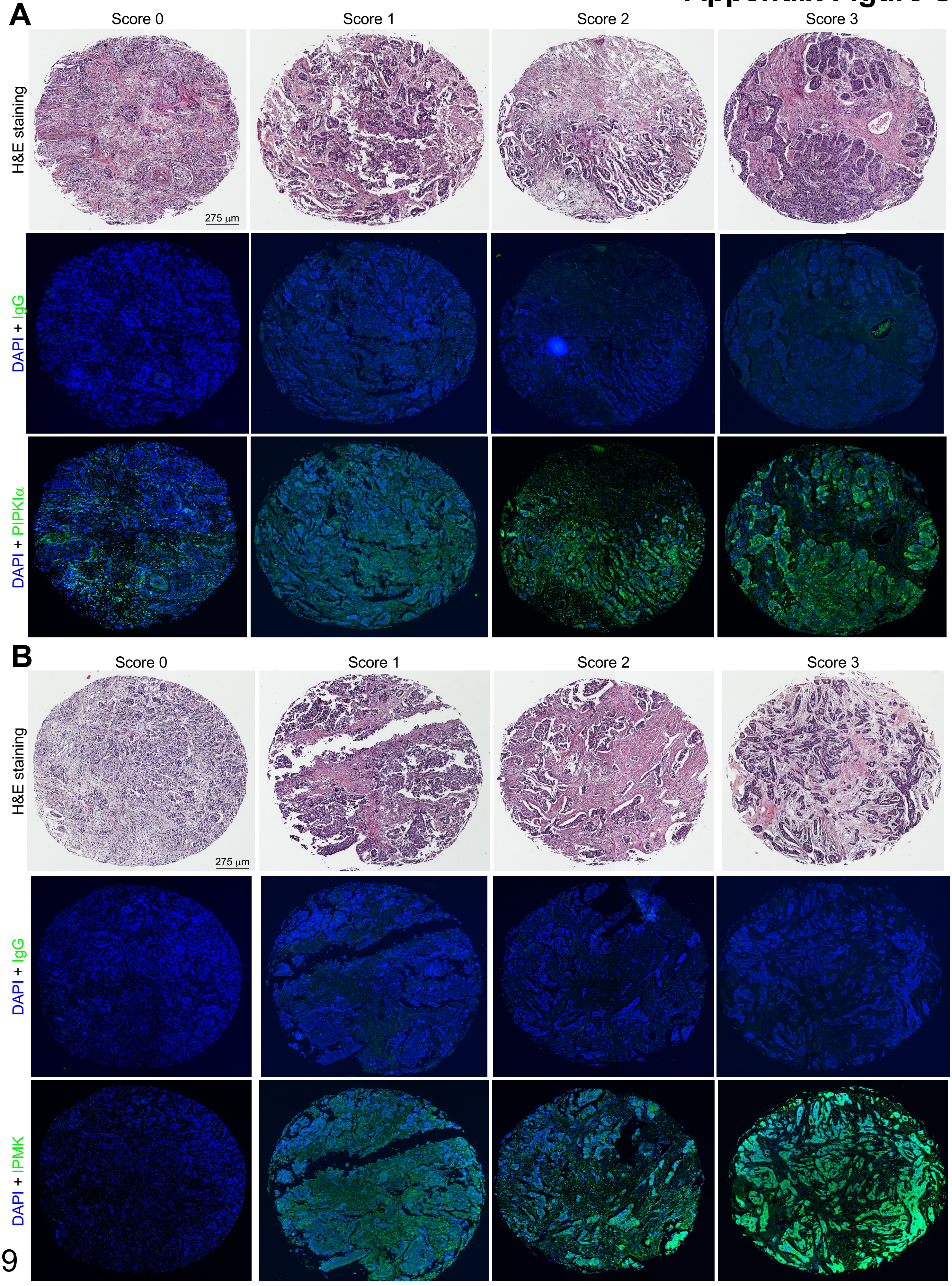

Appendix Figure S3. PIPKIα and IPKM are overexpressed in breast cancer tissues.

A, **B**. Normal and breast cancer biopsy tissues were stained with anti-PIPKIα or anti-IPMK antibodies. The tumor areas in the tissues were localized by H&E staining and the staining intensity of PIPKIα and IPMK were histologically scored with a 0, 1, 2, and 3 scale. Representative images of each score are shown. Scale bar, 275 μm.
