# Supplementary material for: Nuclear phosphoinositide signaling promotes YAP/TAZ-TEAD transcriptional activity in breast cancer
Source: EMBO J. 2024 Apr 2;43(9):4. doi: 10.1038/s44318-024-00085-6 (PMC11066040; doi:10.1038/s44318-024-00085-6)
Supplement: Supplementary file 2 — Source data Fig. 1 [file 44318_2024_85_MOESM2_ESM.zip › SD Figure 1/1A.pptx]

## Slide 1
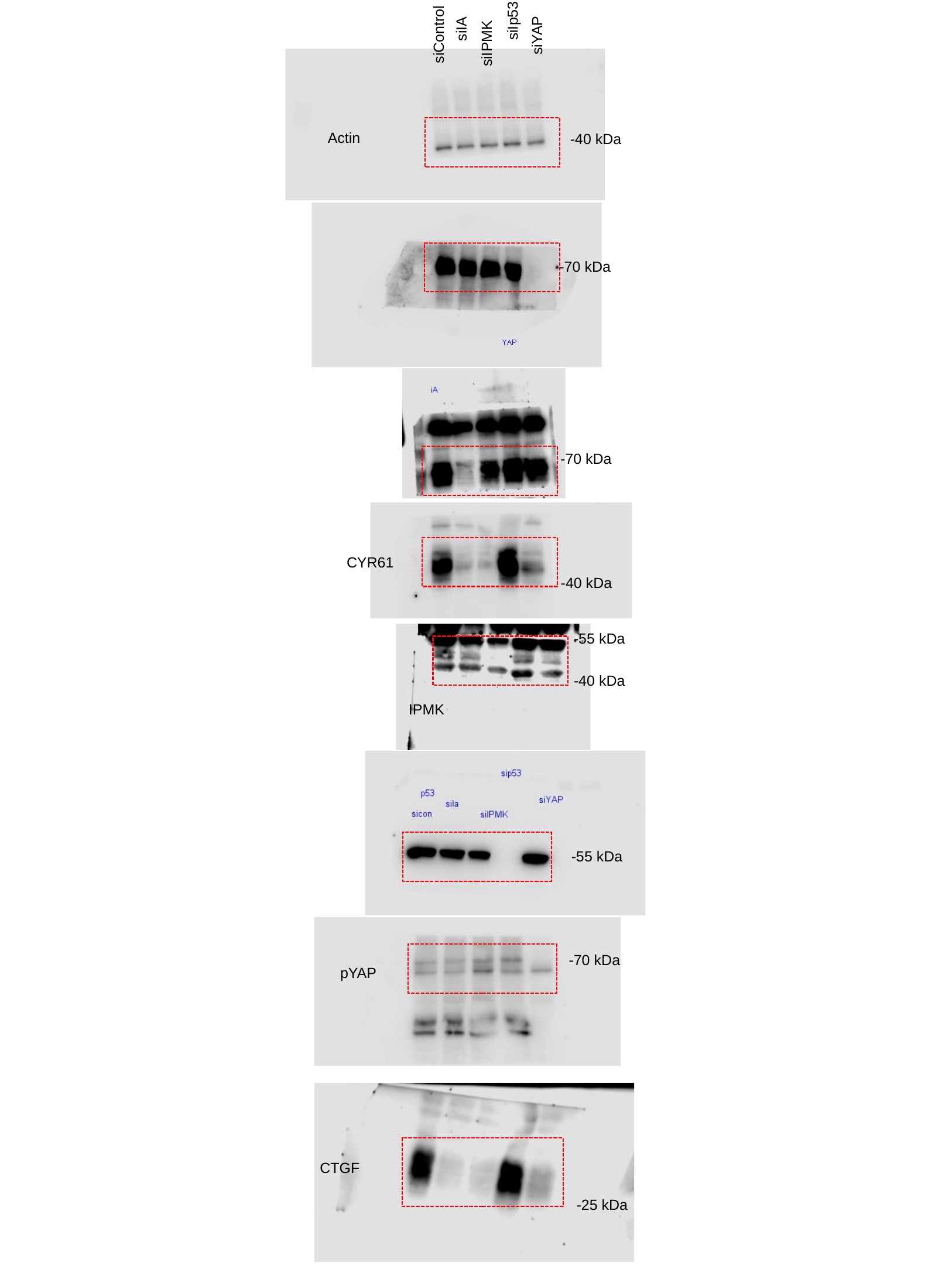

siIp53
siIA
siControl
siYAP
siIPMK
Actin
-40 kDa
-70 kDa
-70 kDa
CYR61
-40 kDa
-55 kDa
-40 kDa
IPMK
-55 kDa
-70 kDa
pYAP
CTGF
-25 kDa
