# Supplementary material for: Nuclear phosphoinositide signaling promotes YAP/TAZ-TEAD transcriptional activity in breast cancer
Source: EMBO J. 2024 Apr 2;43(9):4. doi: 10.1038/s44318-024-00085-6 (PMC11066040; doi:10.1038/s44318-024-00085-6)
Supplement: Supplementary file 2 — Source data Fig. 1 [file 44318_2024_85_MOESM2_ESM.zip › SD Figure 1/1F.pptx]

## Slide 1
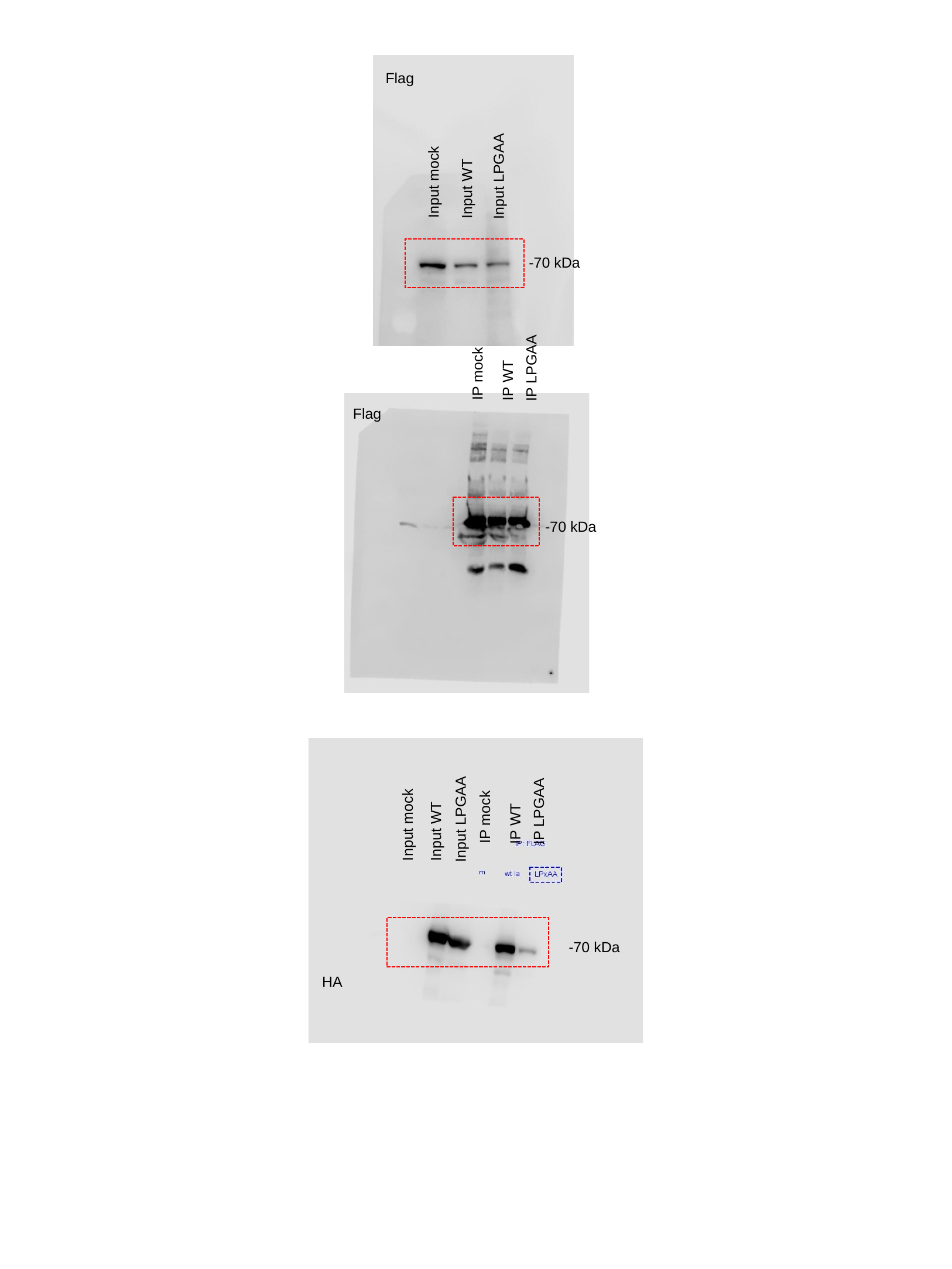

Flag
Input LPGAA
Input mock
Input WT
-70 kDa
IP LPGAA
IP mock
IP WT
Flag
-70 kDa
IP LPGAA
IP mock
Input LPGAA
IP WT
Input mock
Input WT
-70 kDa
HA
