# Supplementary figures and images for: Nuclear phosphoinositide signaling promotes YAP/TAZ-TEAD transcriptional activity in breast cancer
Source: EMBO J. 2024 Apr 2;43(9):4. doi: 10.1038/s44318-024-00085-6 (PMC11066040; doi:10.1038/s44318-024-00085-6)

## Slide 1
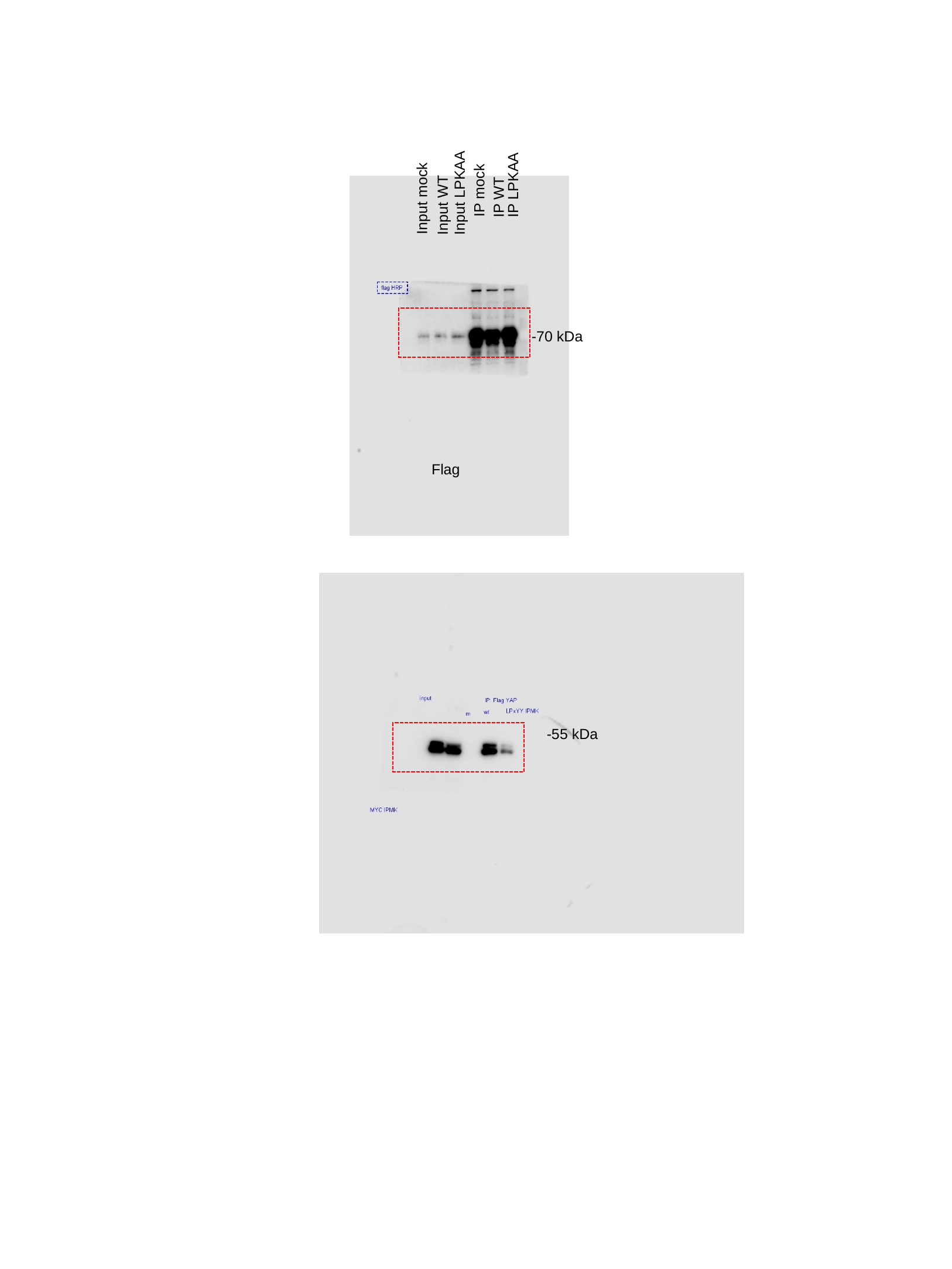

IP LPKAA
IP mock
Input LPKAA
IP WT
Input mock
Input WT
-70 kDa
Flag
-55 kDa

Supplement: Supplementary file 2 — Source data Fig. 1 [file 44318_2024_85_MOESM2_ESM.zip › SD Figure 1/1G.pptx]

## Slide 1
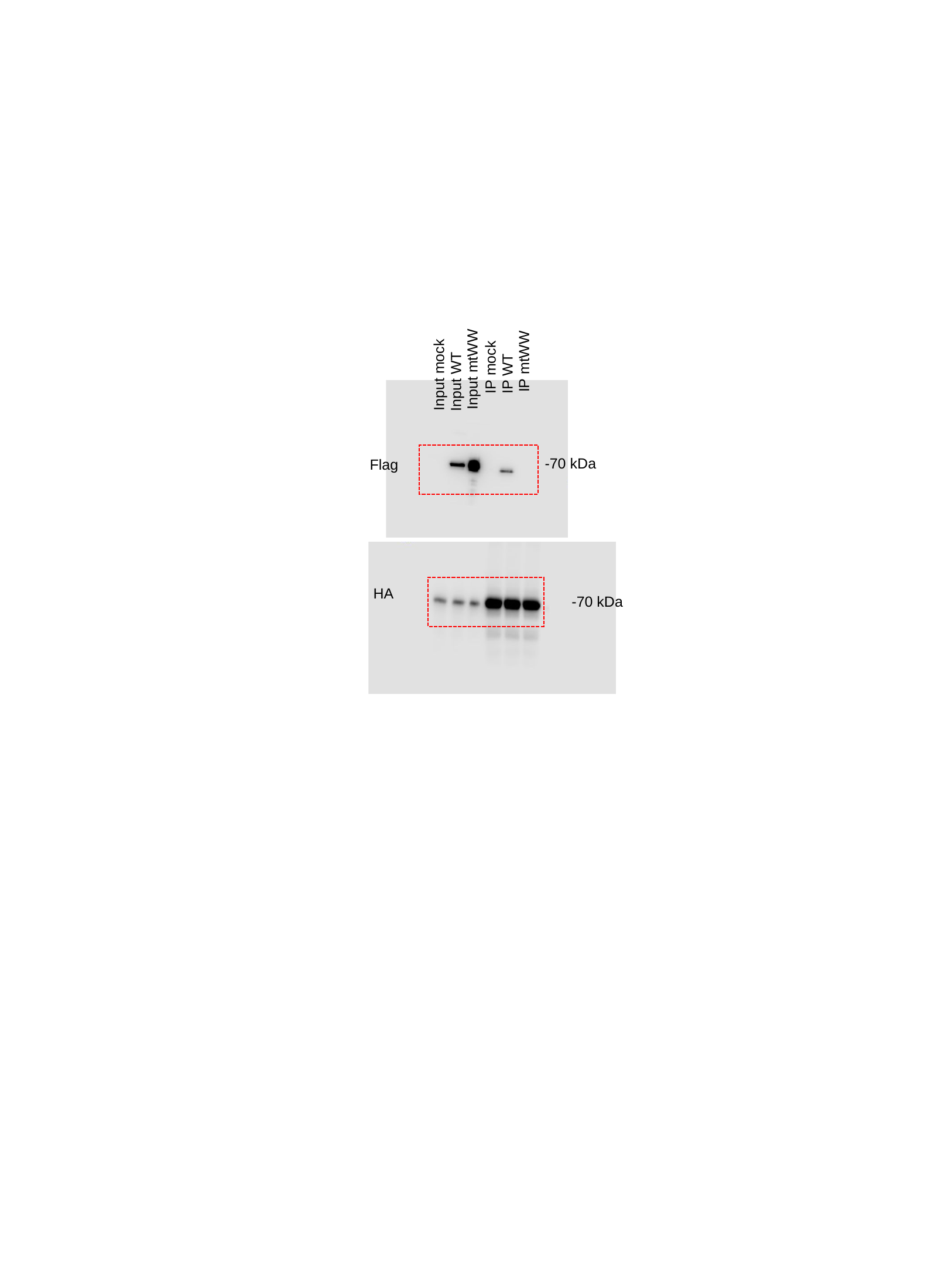

IP mtWW
IP mock
Input mtWW
IP WT
Input mock
Input WT
-70 kDa
Flag
HA
-70 kDa

Supplement: Supplementary file 2 — Source data Fig. 1 [file 44318_2024_85_MOESM2_ESM.zip › SD Figure 1/1D.pptx]

## Slide 1
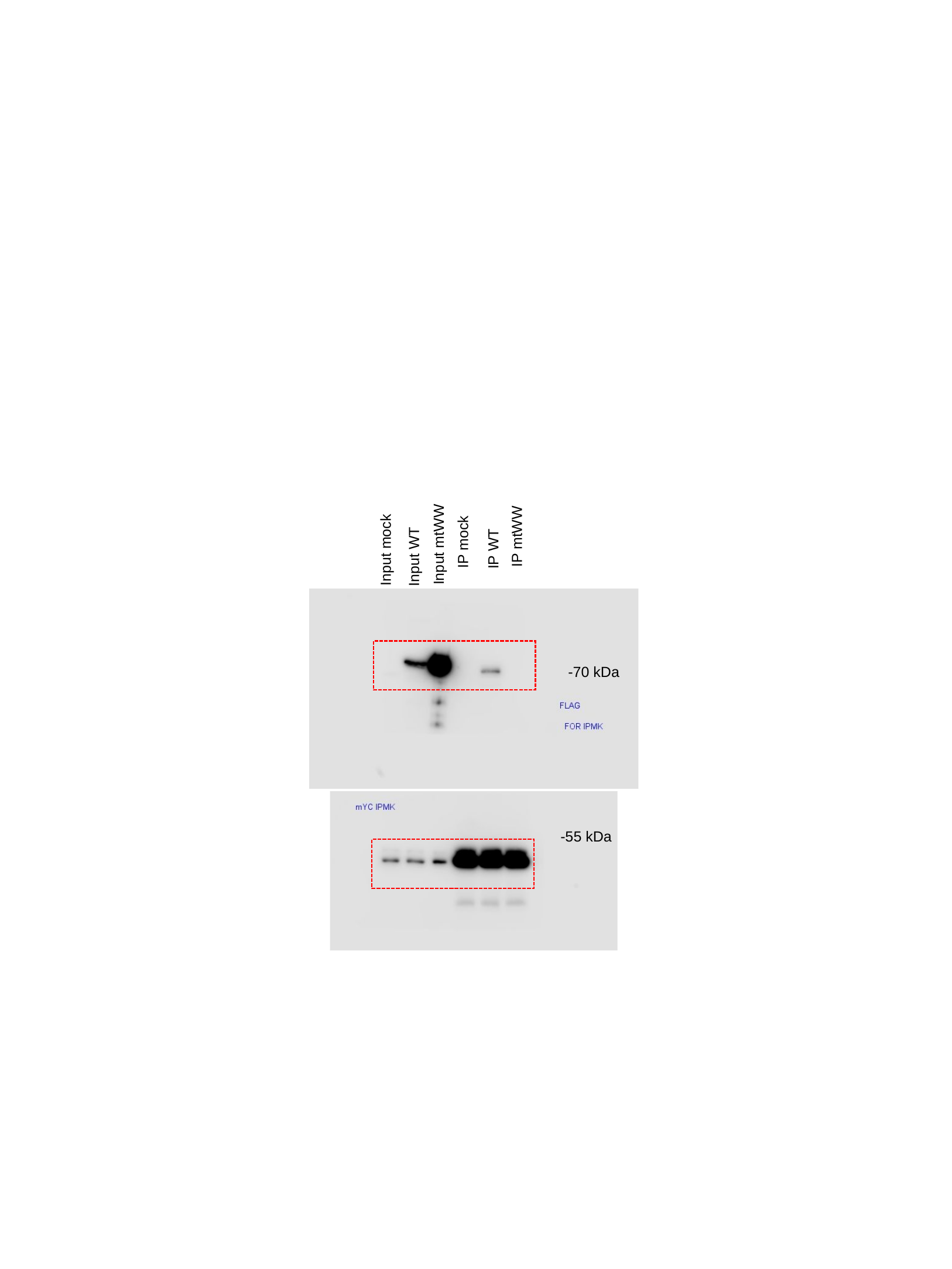

IP mtWW
IP mock
Input mtWW
IP WT
Input mock
Input WT
-70 kDa
-55 kDa

Supplement: Supplementary file 2 — Source data Fig. 1 [file 44318_2024_85_MOESM2_ESM.zip › SD Figure 1/1E.pptx]

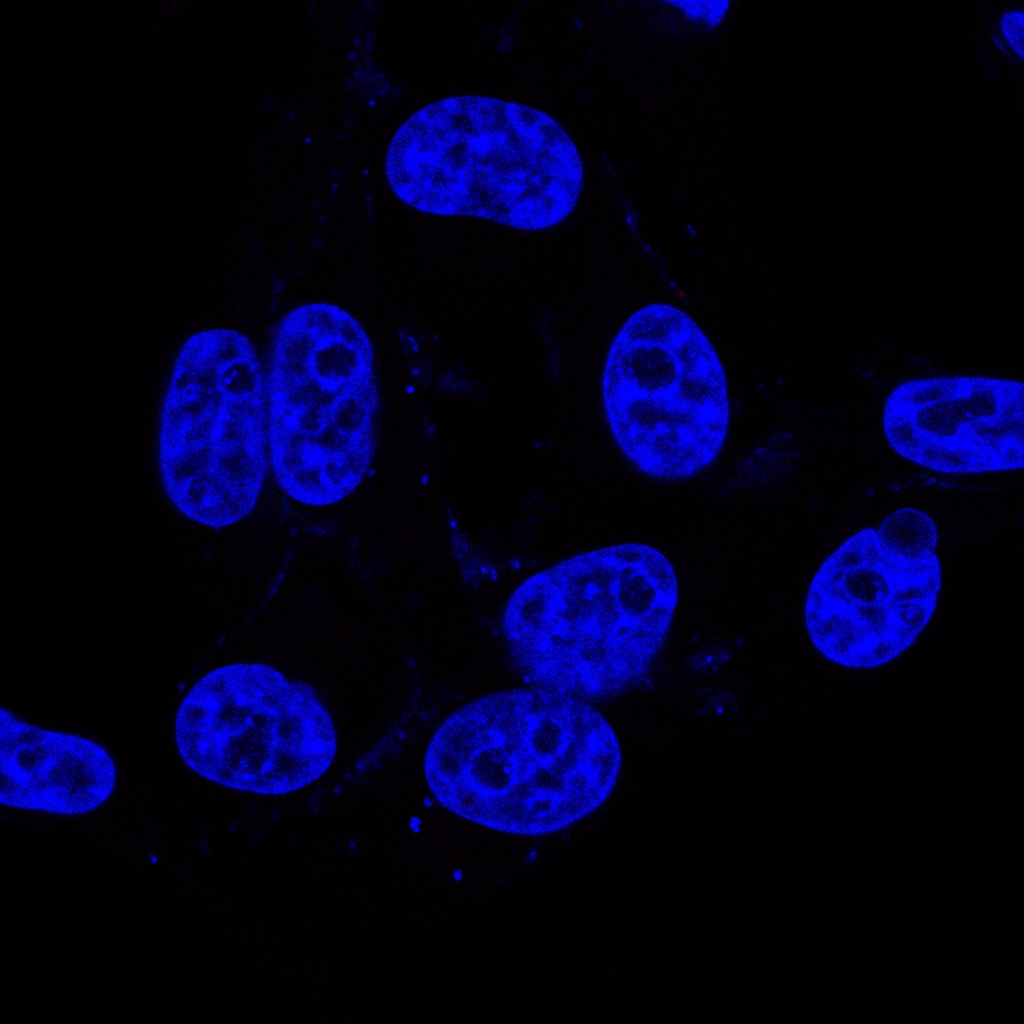

Supplement: Supplementary file 3 — Source data Fig. 2 [file 44318_2024_85_MOESM3_ESM.zip › SD Figure 2/2B high resolution/Free merge.jpg]

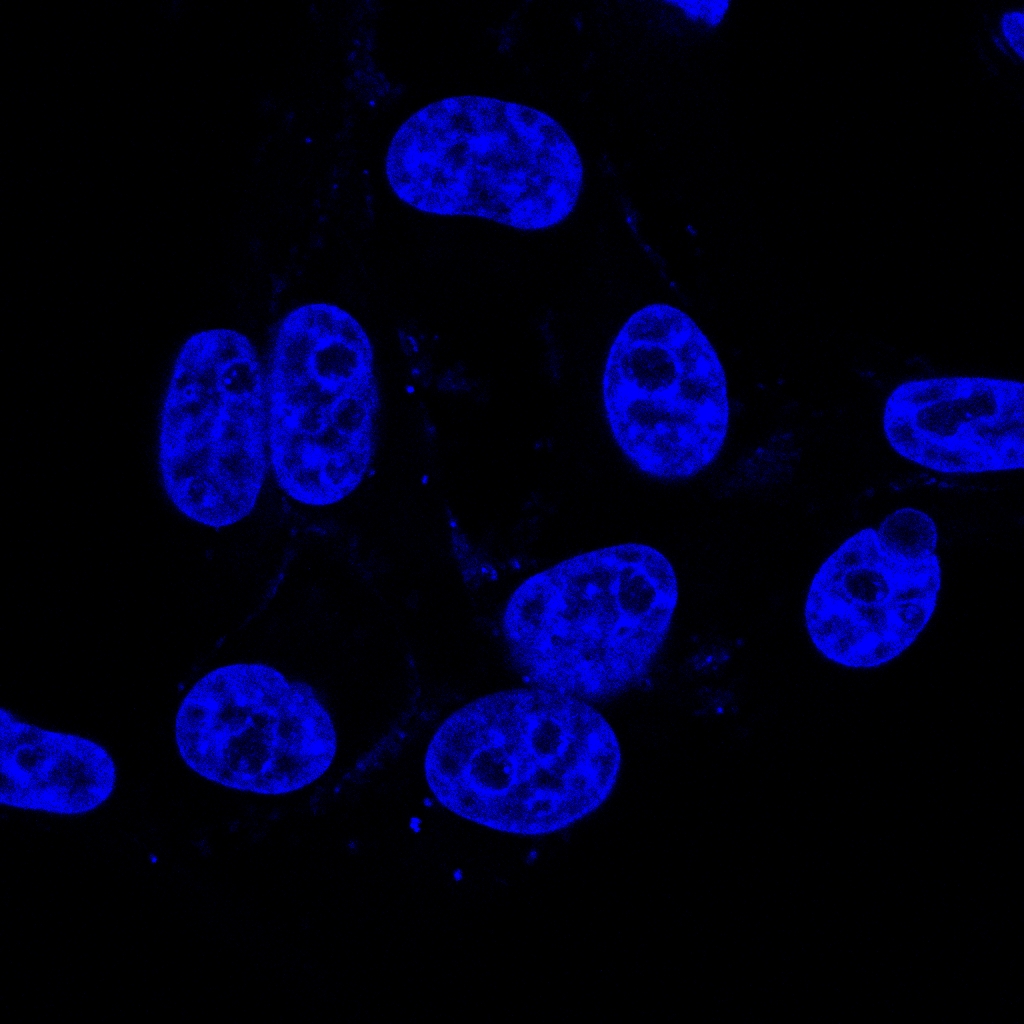

Supplement: Supplementary file 3 — Source data Fig. 2 [file 44318_2024_85_MOESM3_ESM.zip › SD Figure 2/2B high resolution/Free DAPI.jpg]

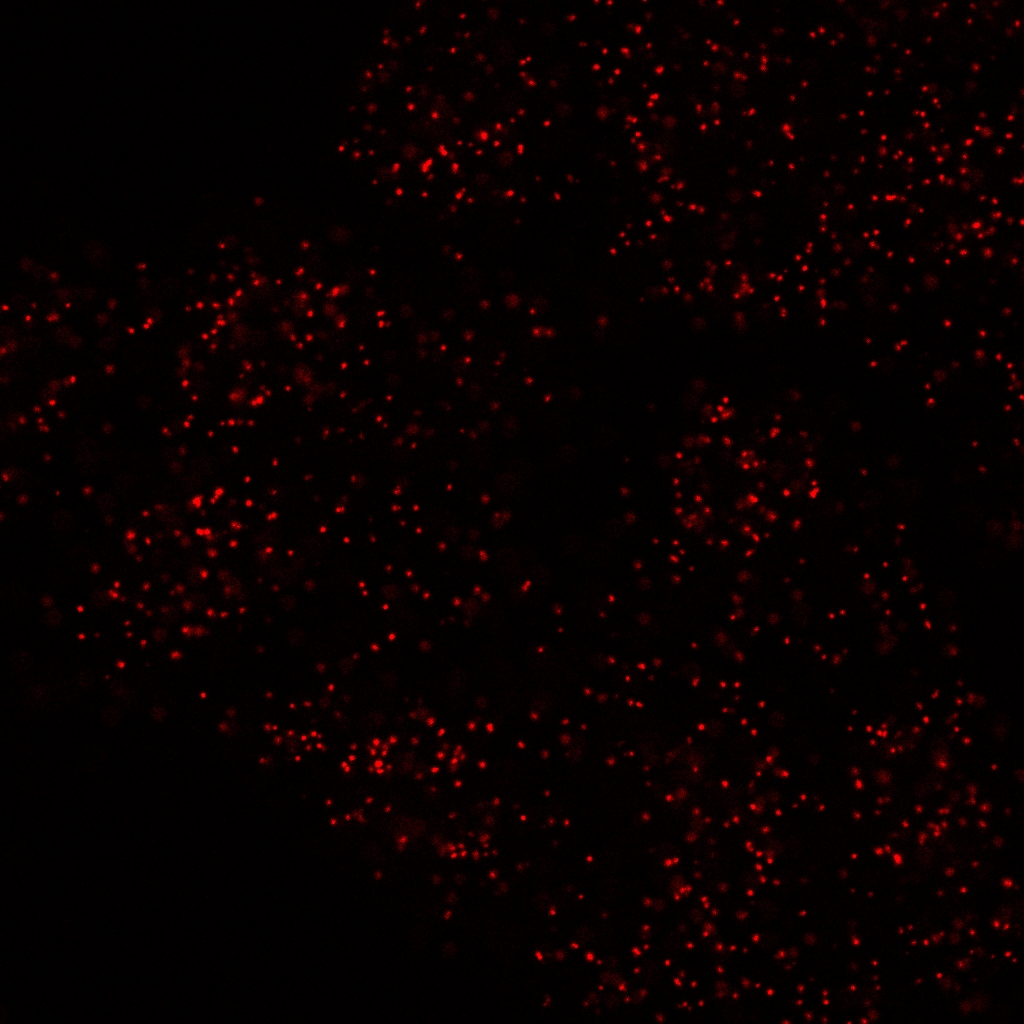

Supplement: Supplementary file 3 — Source data Fig. 2 [file 44318_2024_85_MOESM3_ESM.zip › SD Figure 2/2B high resolution/Serum PLA.jpg]

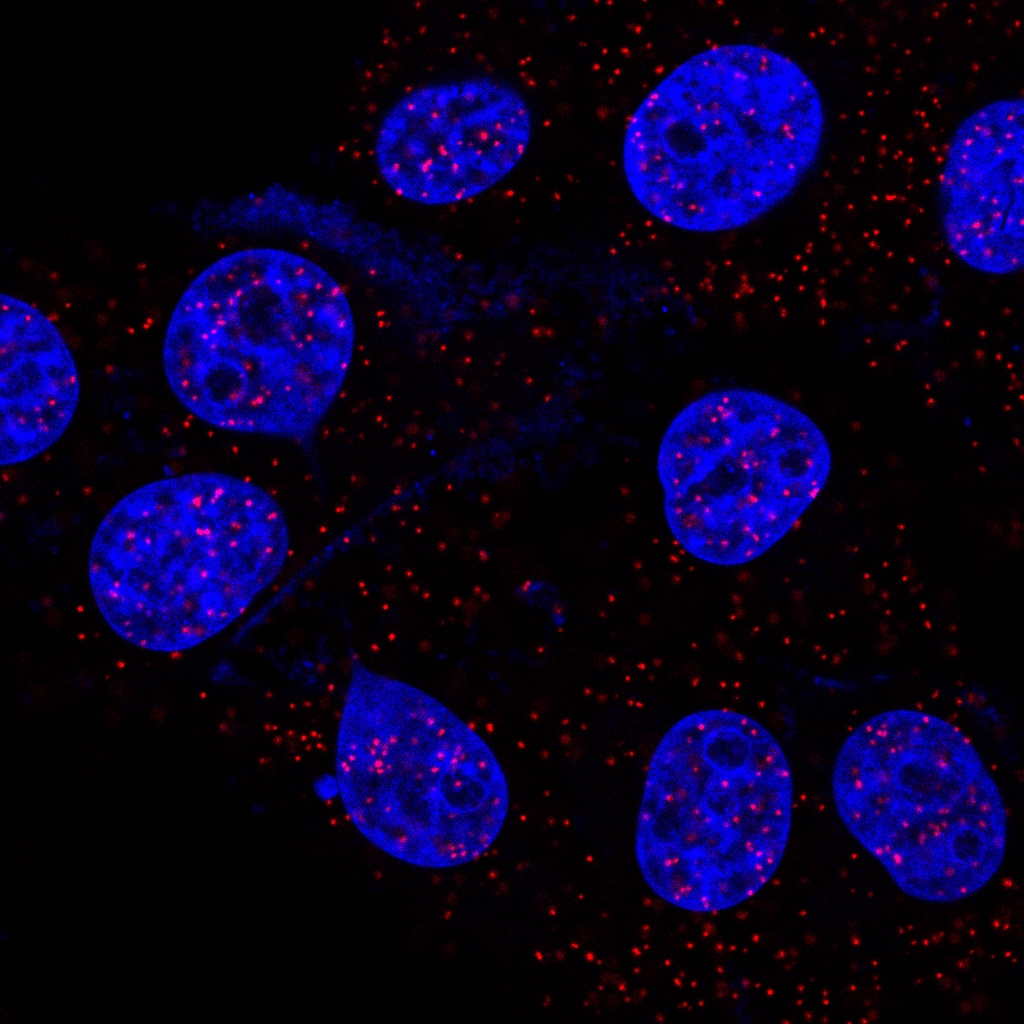

Supplement: Supplementary file 3 — Source data Fig. 2 [file 44318_2024_85_MOESM3_ESM.zip › SD Figure 2/2B high resolution/Serum merge.jpg]

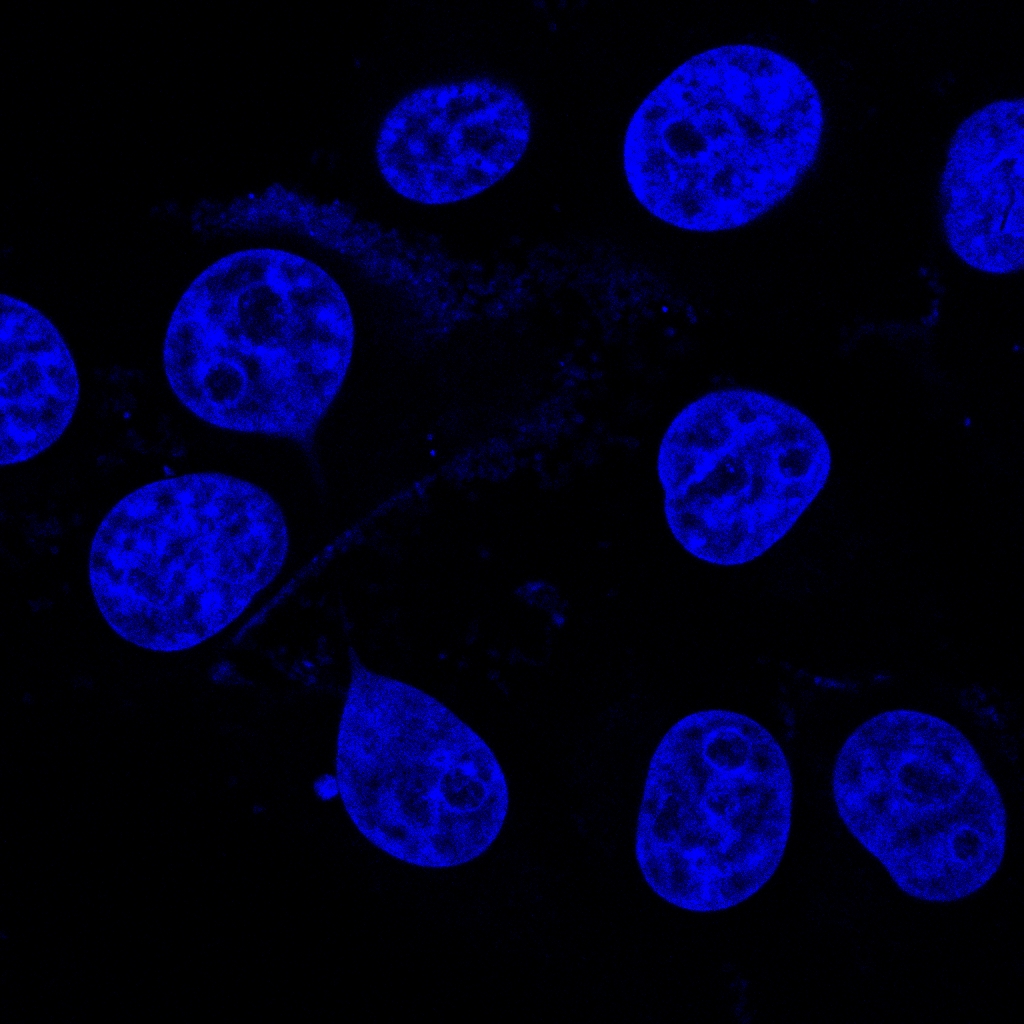

Supplement: Supplementary file 3 — Source data Fig. 2 [file 44318_2024_85_MOESM3_ESM.zip › SD Figure 2/2B high resolution/Serum DAPI.jpg]

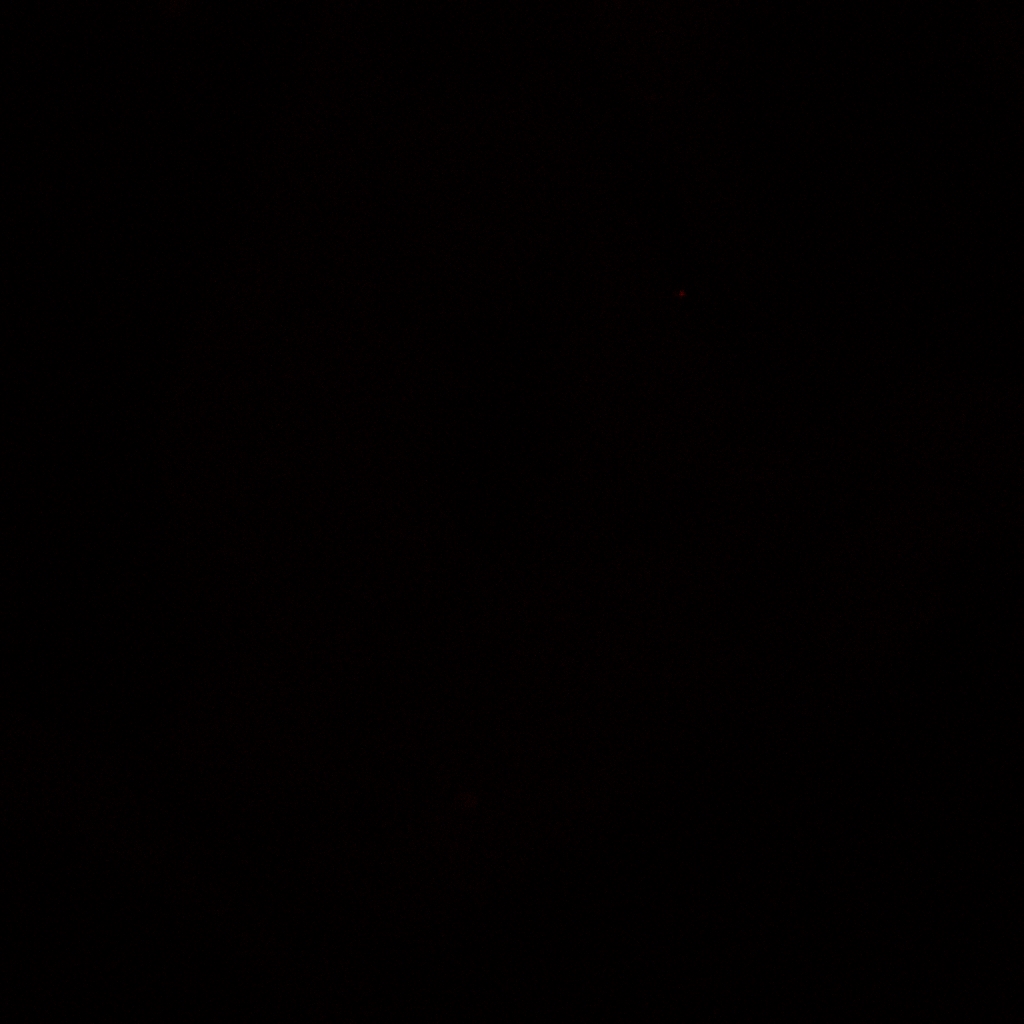

Supplement: Supplementary file 3 — Source data Fig. 2 [file 44318_2024_85_MOESM3_ESM.zip › SD Figure 2/2B high resolution/Free PLA.jpg]

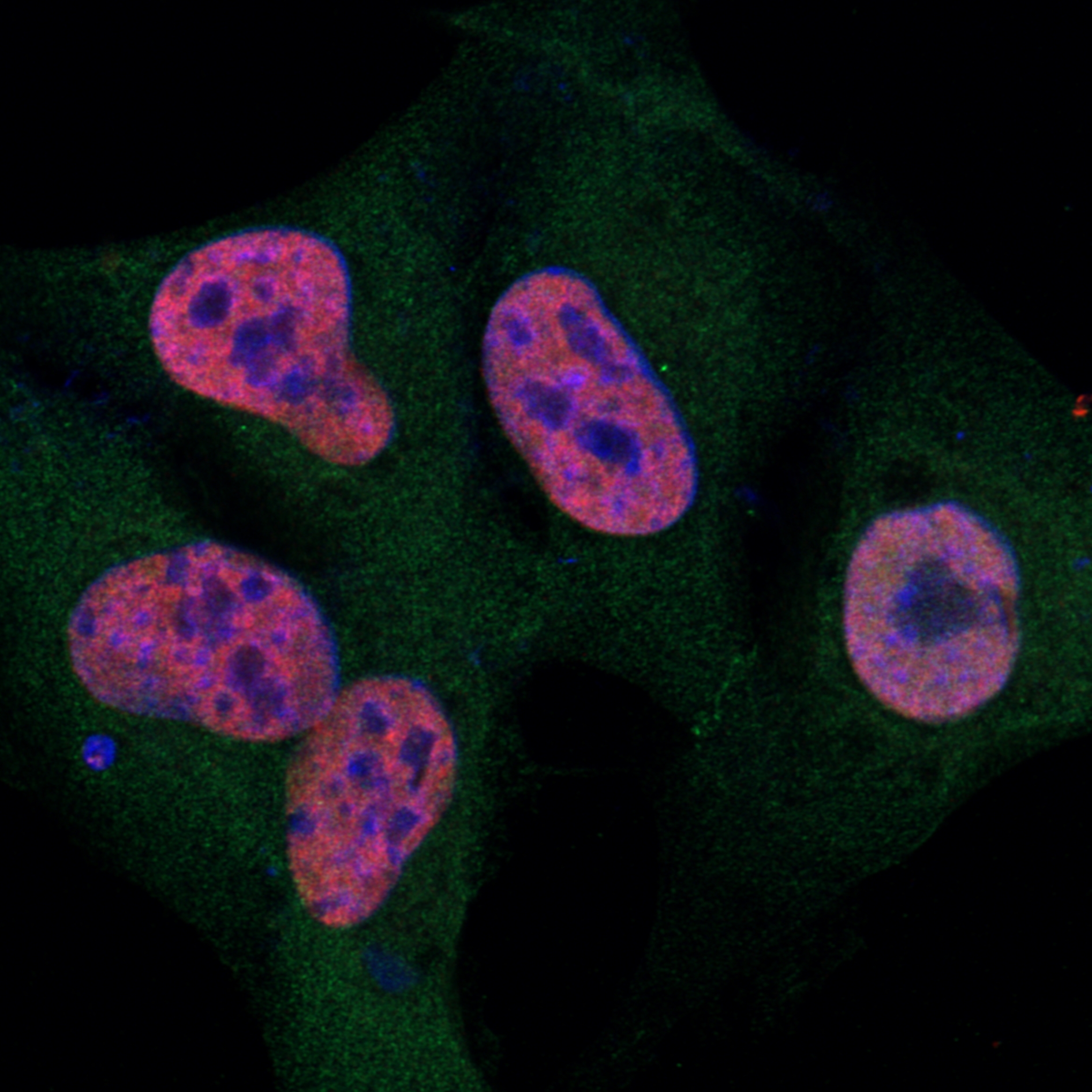

Supplement: Supplementary file 3 — Source data Fig. 2 [file 44318_2024_85_MOESM3_ESM.zip › SD Figure 2/2D high resolution/Free merge.jpg]

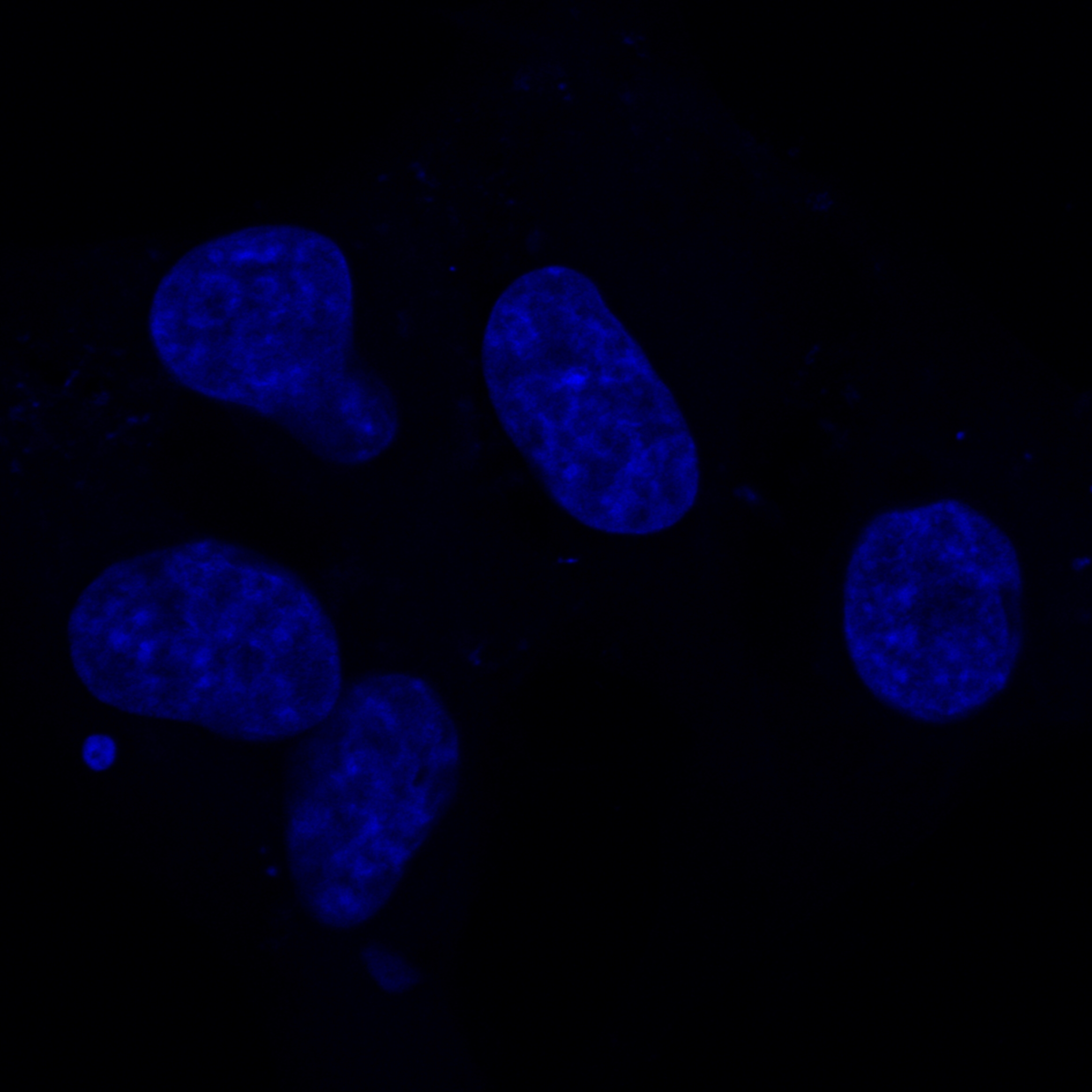

Supplement: Supplementary file 3 — Source data Fig. 2 [file 44318_2024_85_MOESM3_ESM.zip › SD Figure 2/2D high resolution/Free DAPI.jpg]

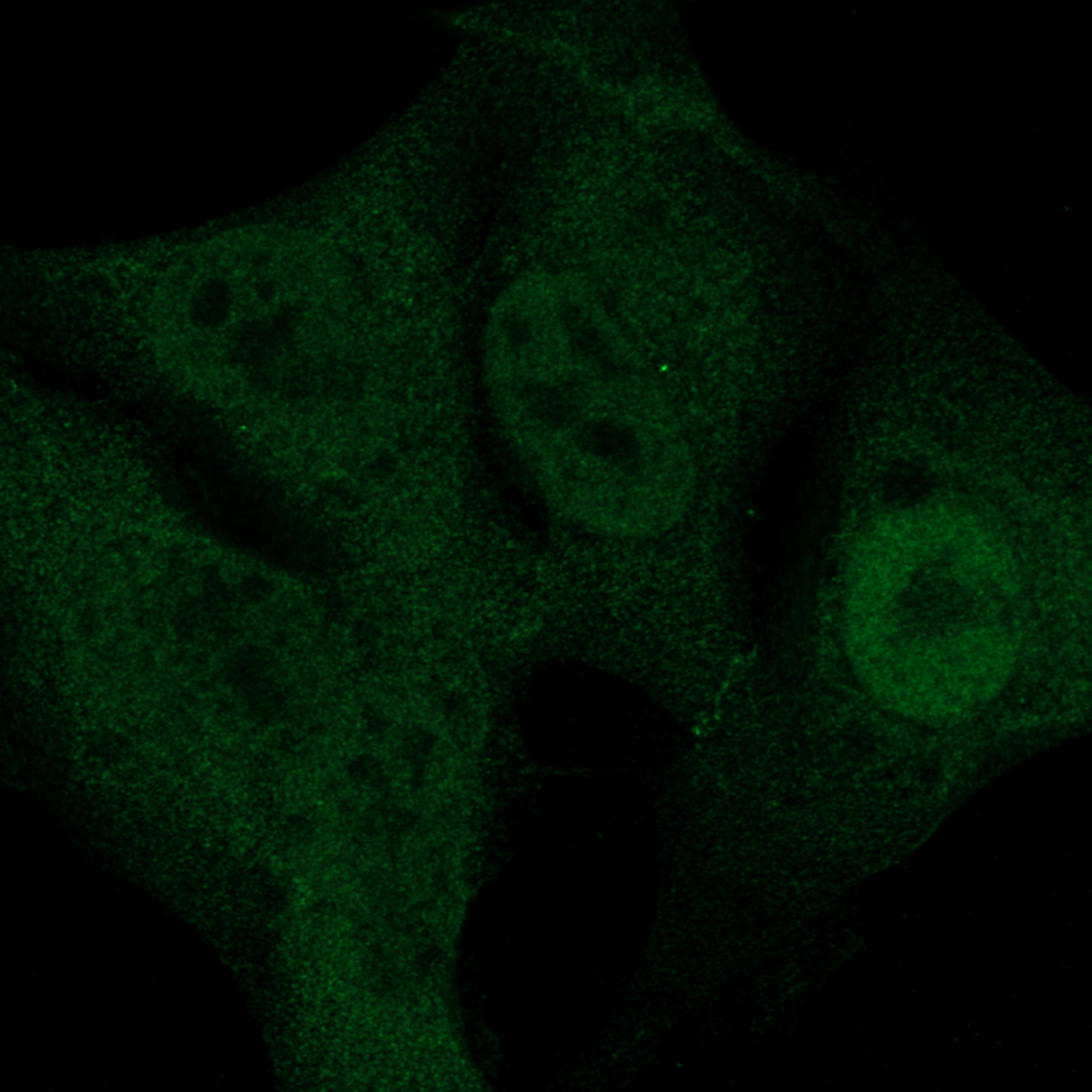

Supplement: Supplementary file 3 — Source data Fig. 2 [file 44318_2024_85_MOESM3_ESM.zip › SD Figure 2/2D high resolution/Free YAP.jpg]

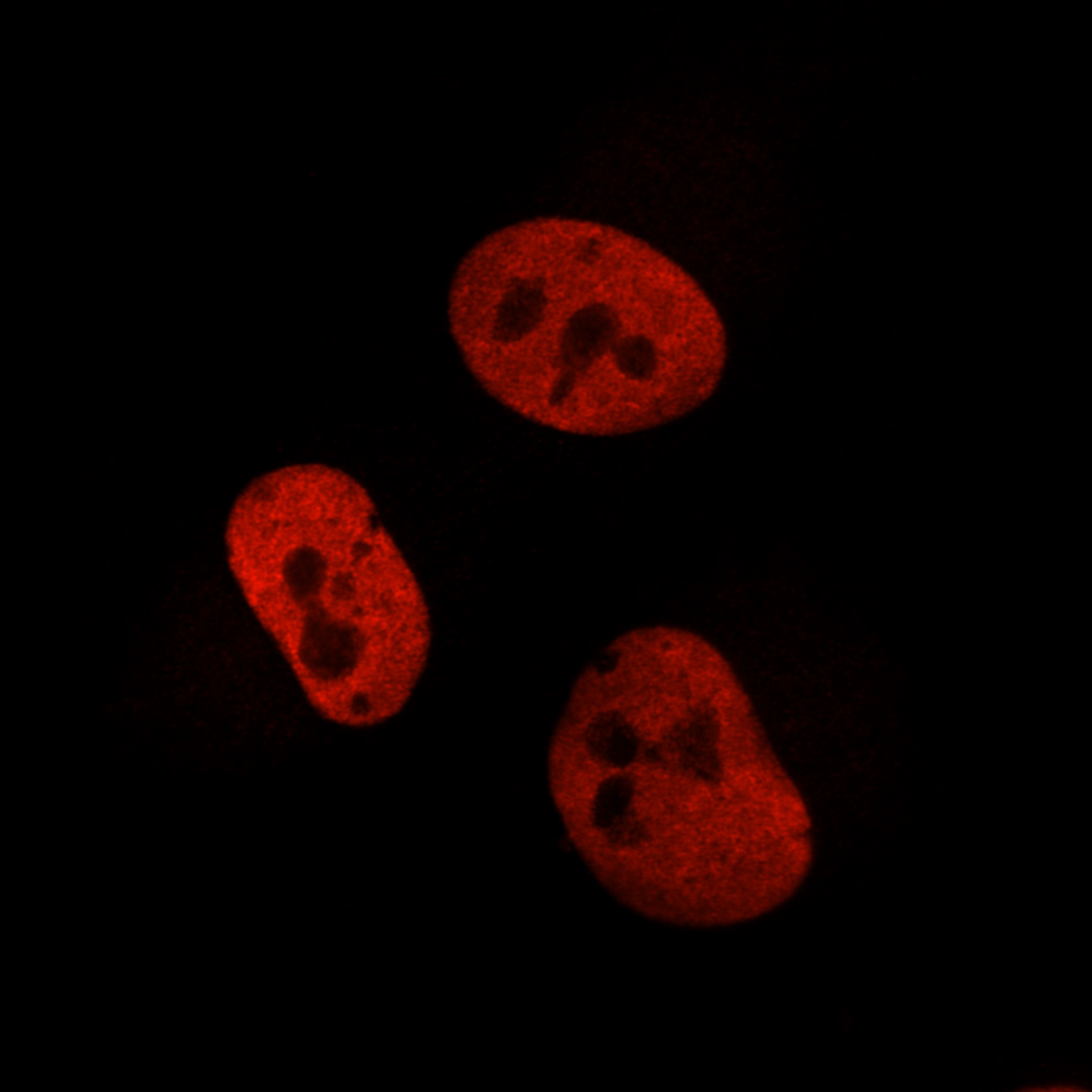

Supplement: Supplementary file 3 — Source data Fig. 2 [file 44318_2024_85_MOESM3_ESM.zip › SD Figure 2/2D high resolution/Serum IPMK.jpg]

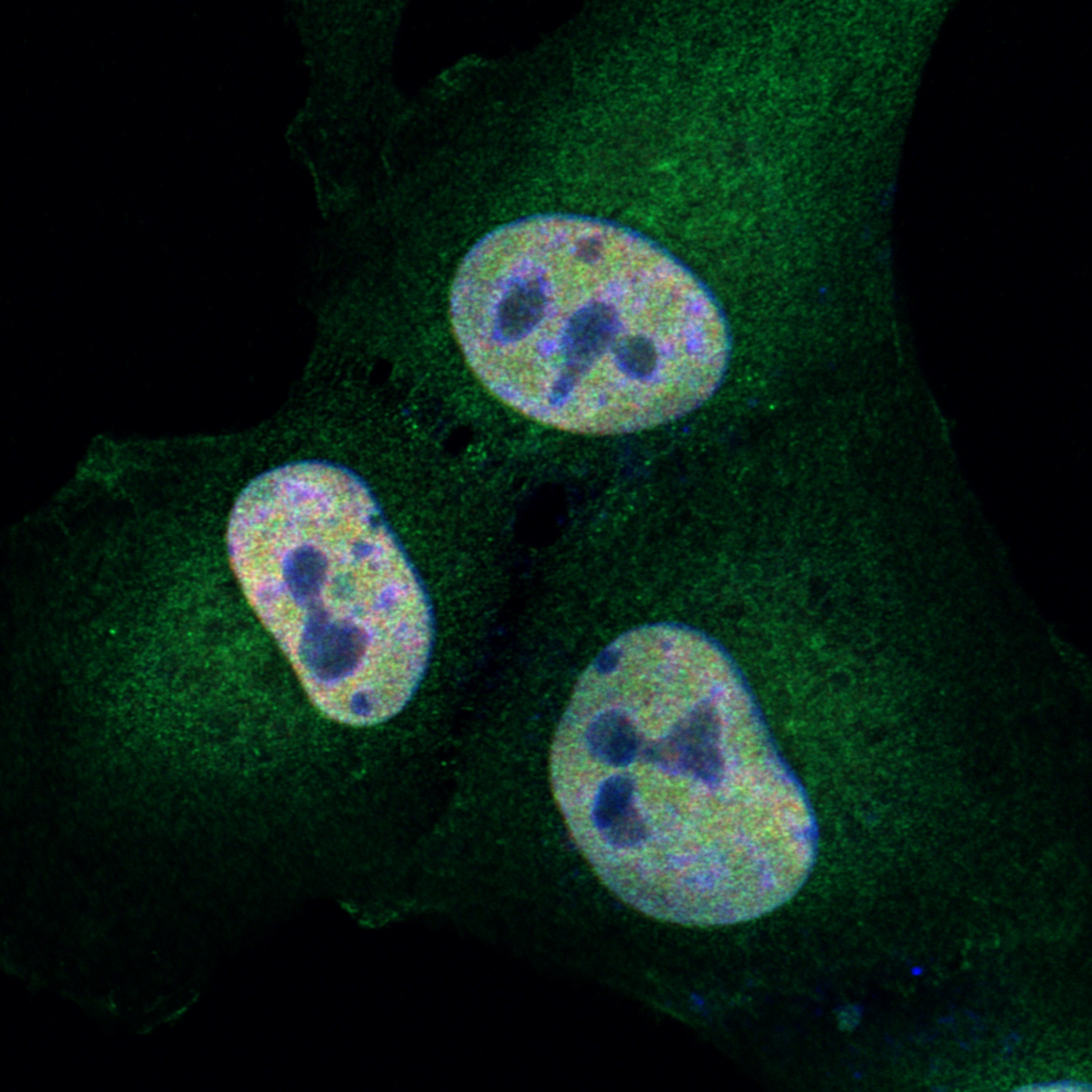

Supplement: Supplementary file 3 — Source data Fig. 2 [file 44318_2024_85_MOESM3_ESM.zip › SD Figure 2/2D high resolution/Serum merge.jpg]

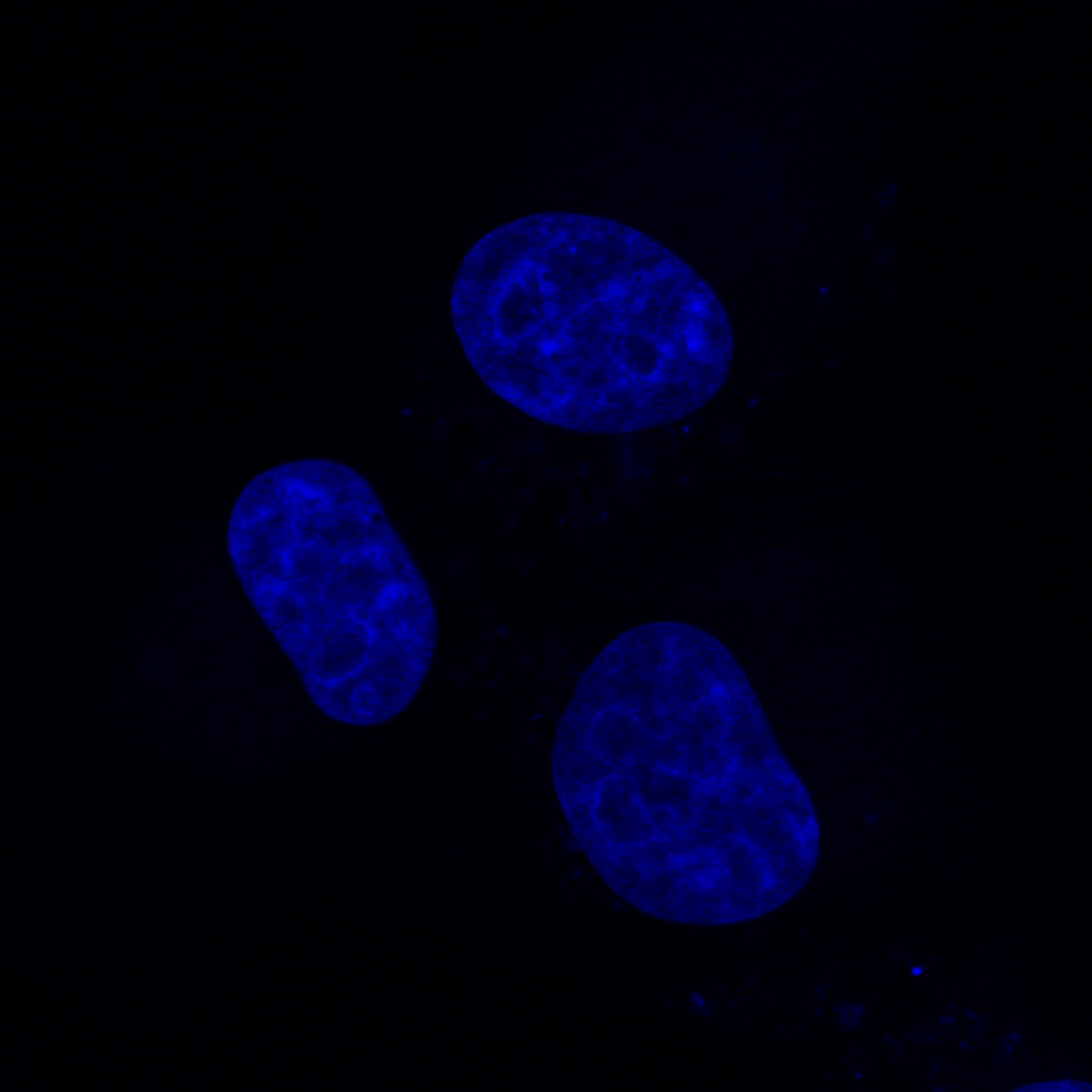

Supplement: Supplementary file 3 — Source data Fig. 2 [file 44318_2024_85_MOESM3_ESM.zip › SD Figure 2/2D high resolution/Serum DAPI.jpg]

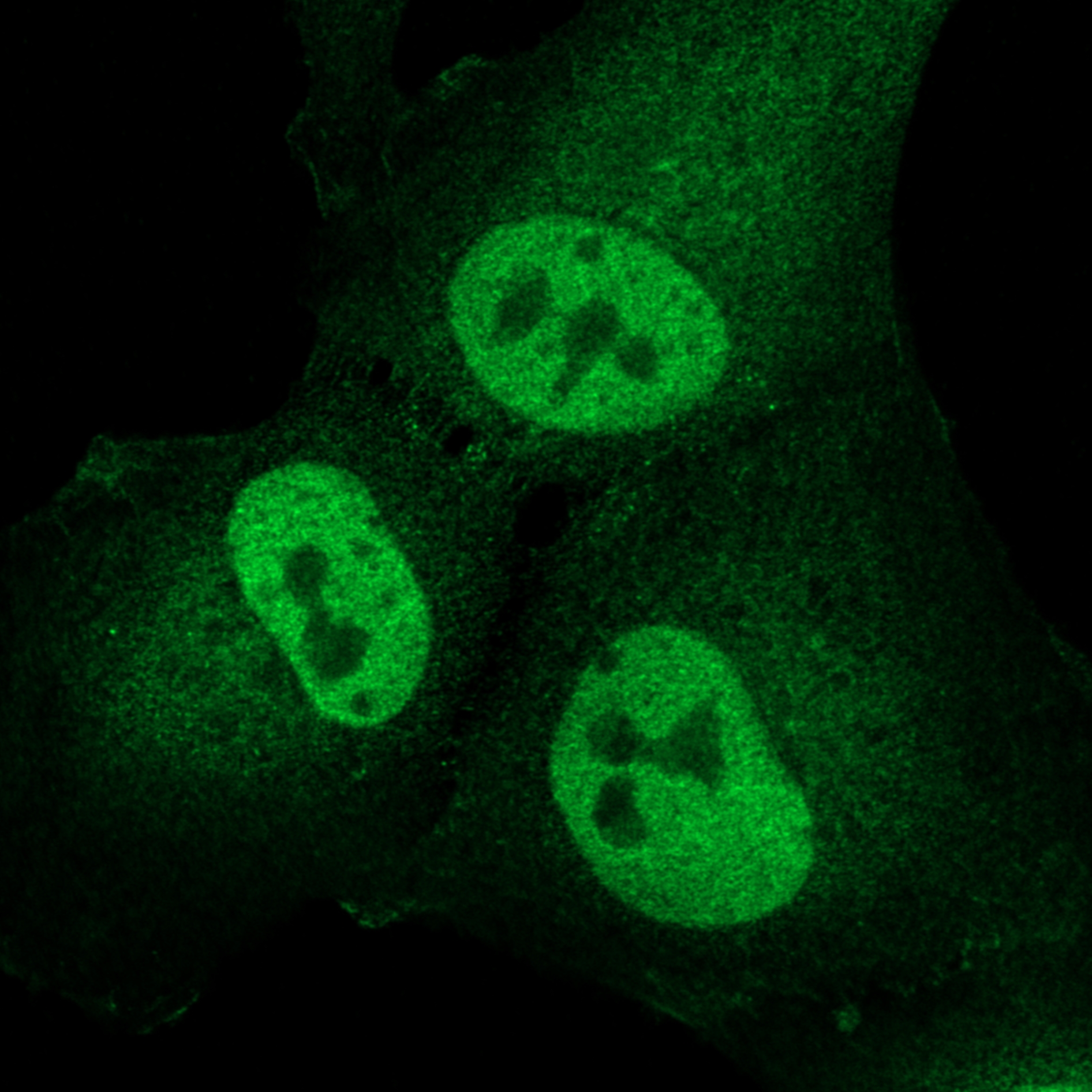

Supplement: Supplementary file 3 — Source data Fig. 2 [file 44318_2024_85_MOESM3_ESM.zip › SD Figure 2/2D high resolution/Serum YAP.jpg]

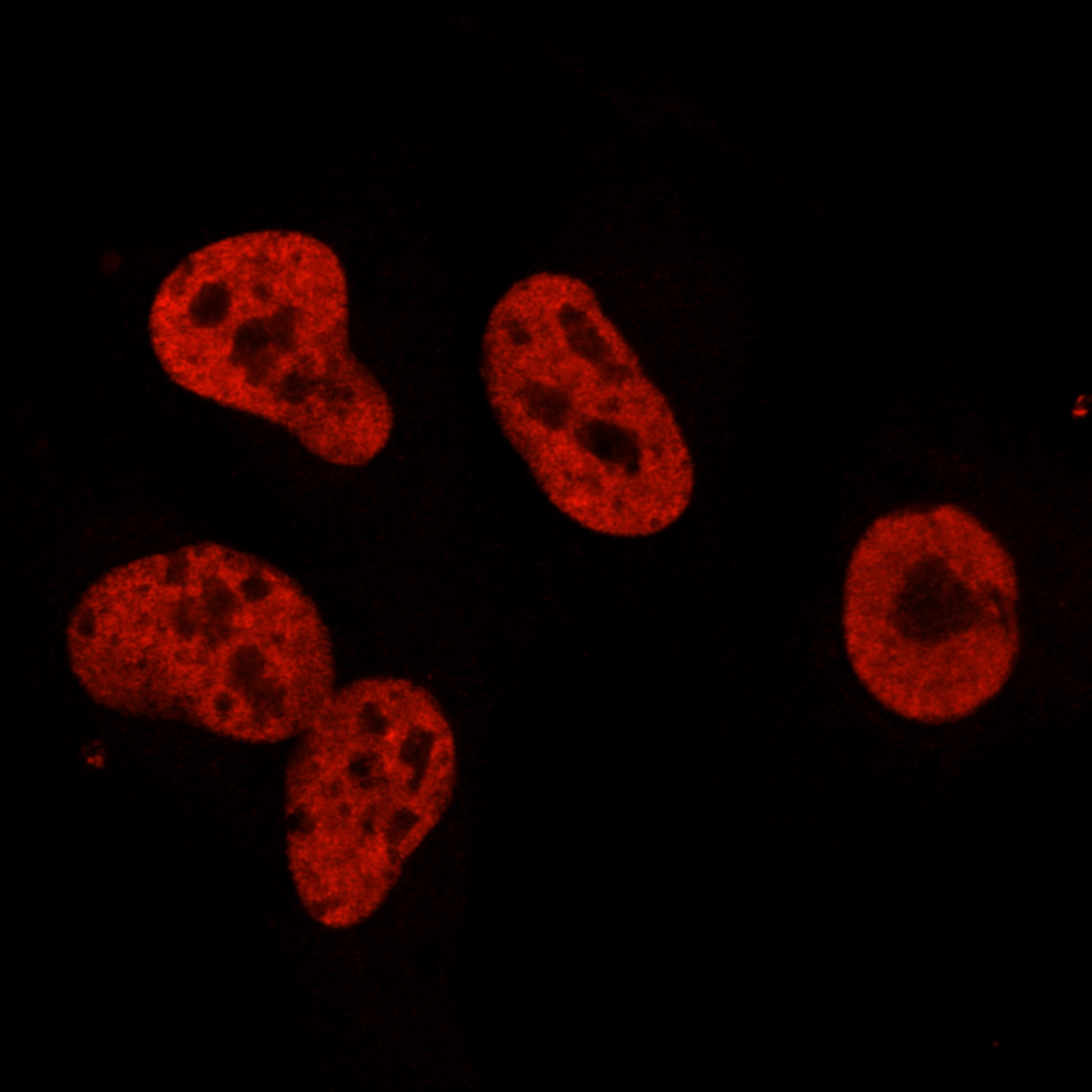

Supplement: Supplementary file 3 — Source data Fig. 2 [file 44318_2024_85_MOESM3_ESM.zip › SD Figure 2/2D high resolution/Free IPMK.jpg]

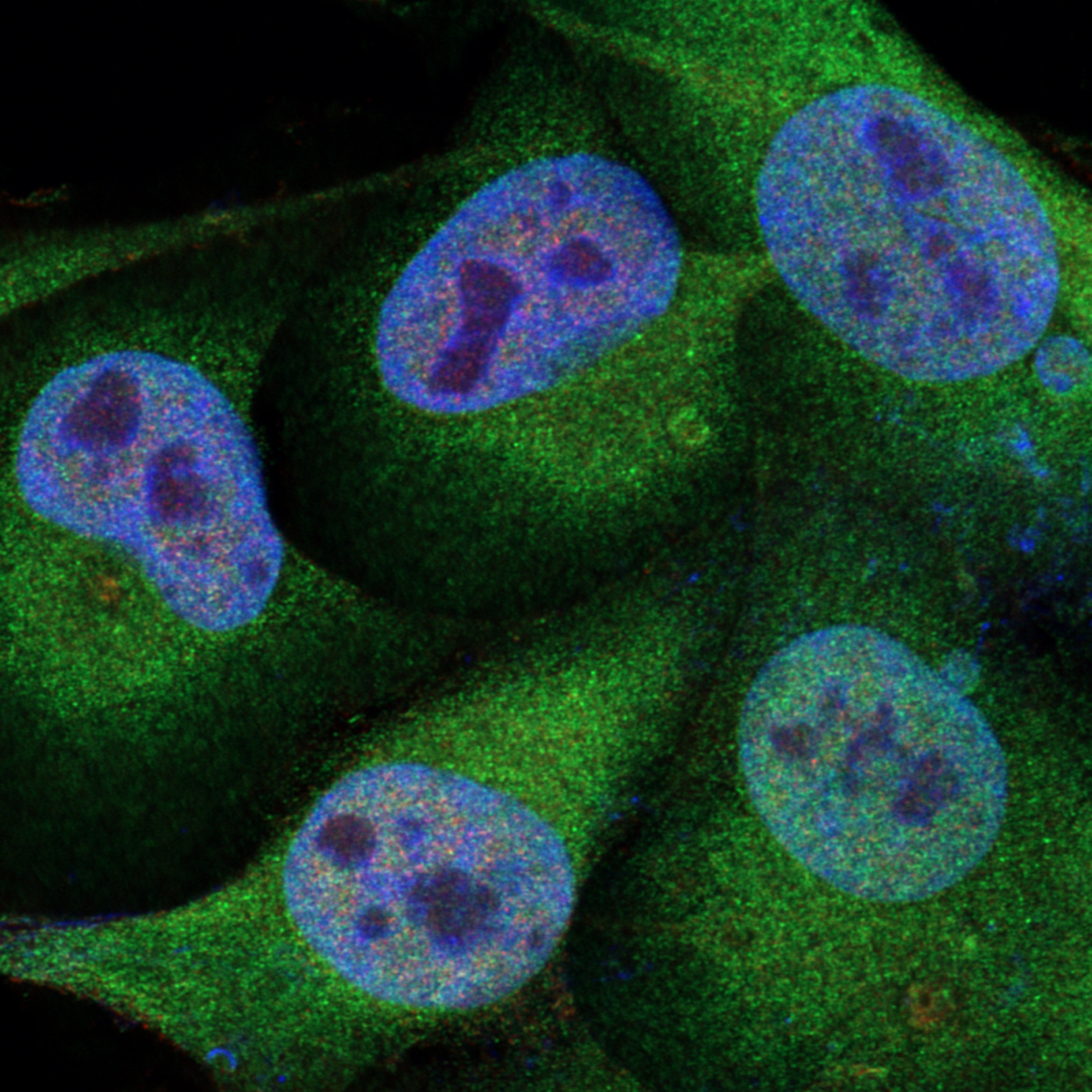

Supplement: Supplementary file 3 — Source data Fig. 2 [file 44318_2024_85_MOESM3_ESM.zip › SD Figure 2/2C high resolution/Free merge.jpg]

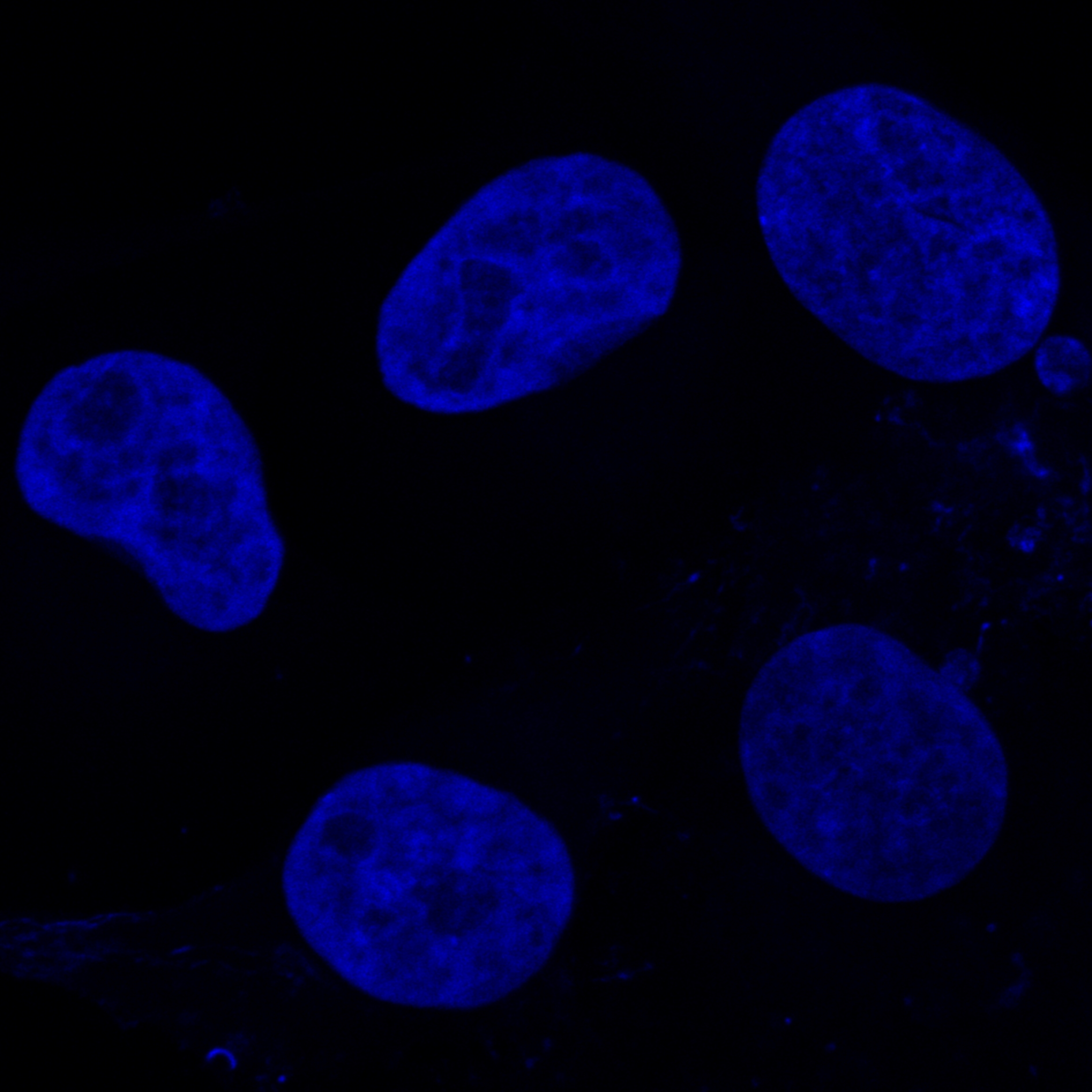

Supplement: Supplementary file 3 — Source data Fig. 2 [file 44318_2024_85_MOESM3_ESM.zip › SD Figure 2/2C high resolution/Free DAPI.jpg]

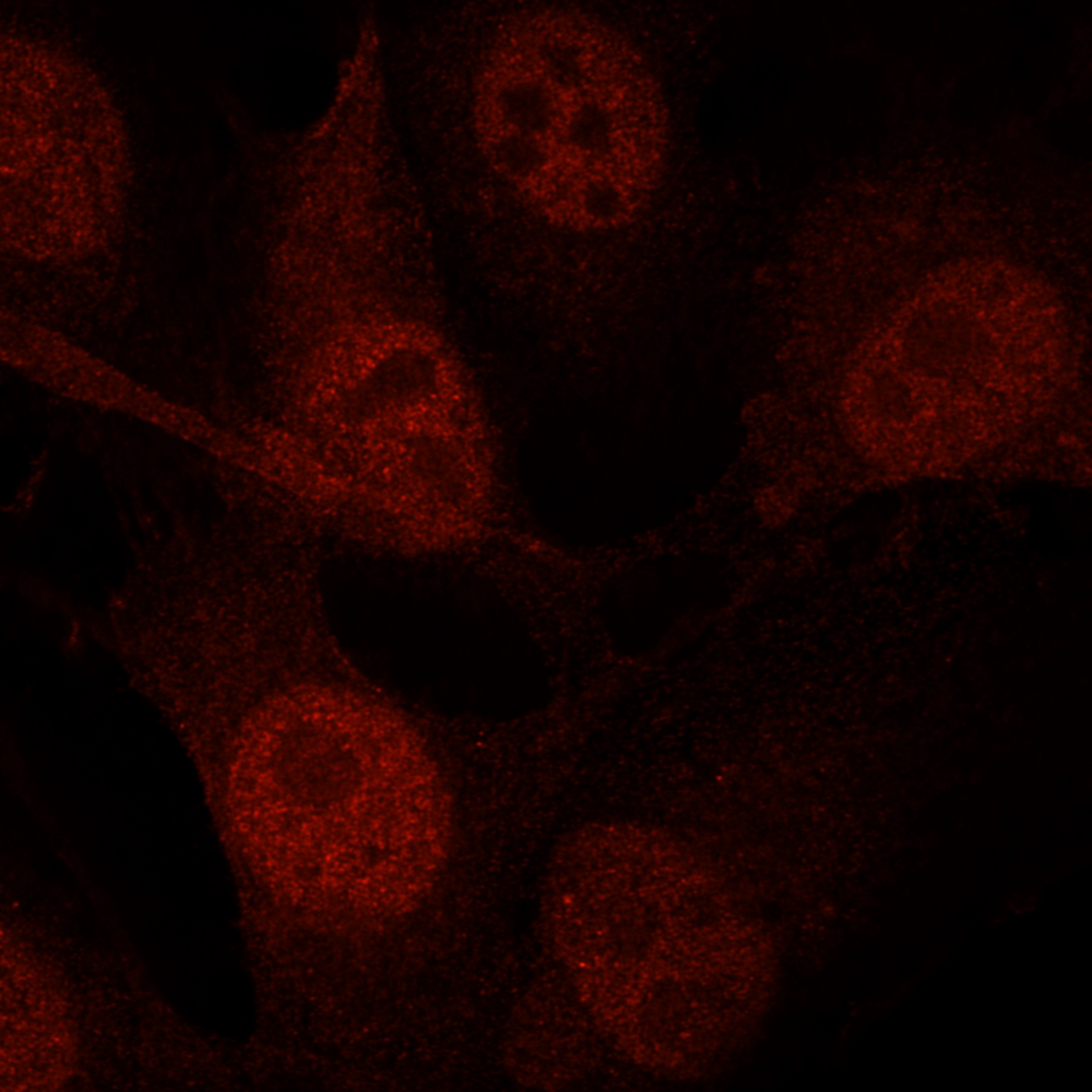

Supplement: Supplementary file 3 — Source data Fig. 2 [file 44318_2024_85_MOESM3_ESM.zip › SD Figure 2/2C high resolution/Serum IA.jpg]

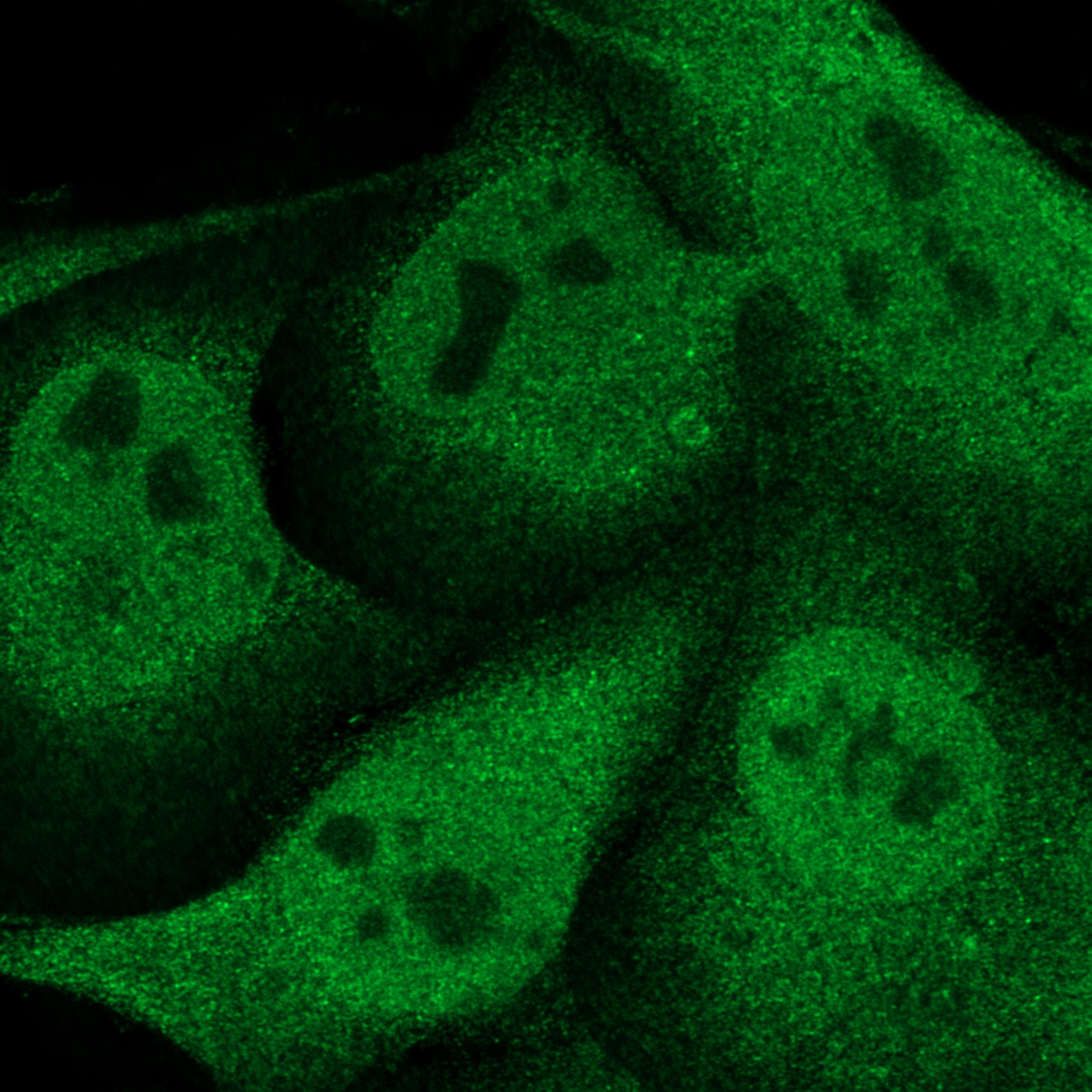

Supplement: Supplementary file 3 — Source data Fig. 2 [file 44318_2024_85_MOESM3_ESM.zip › SD Figure 2/2C high resolution/Free YAP.jpg]

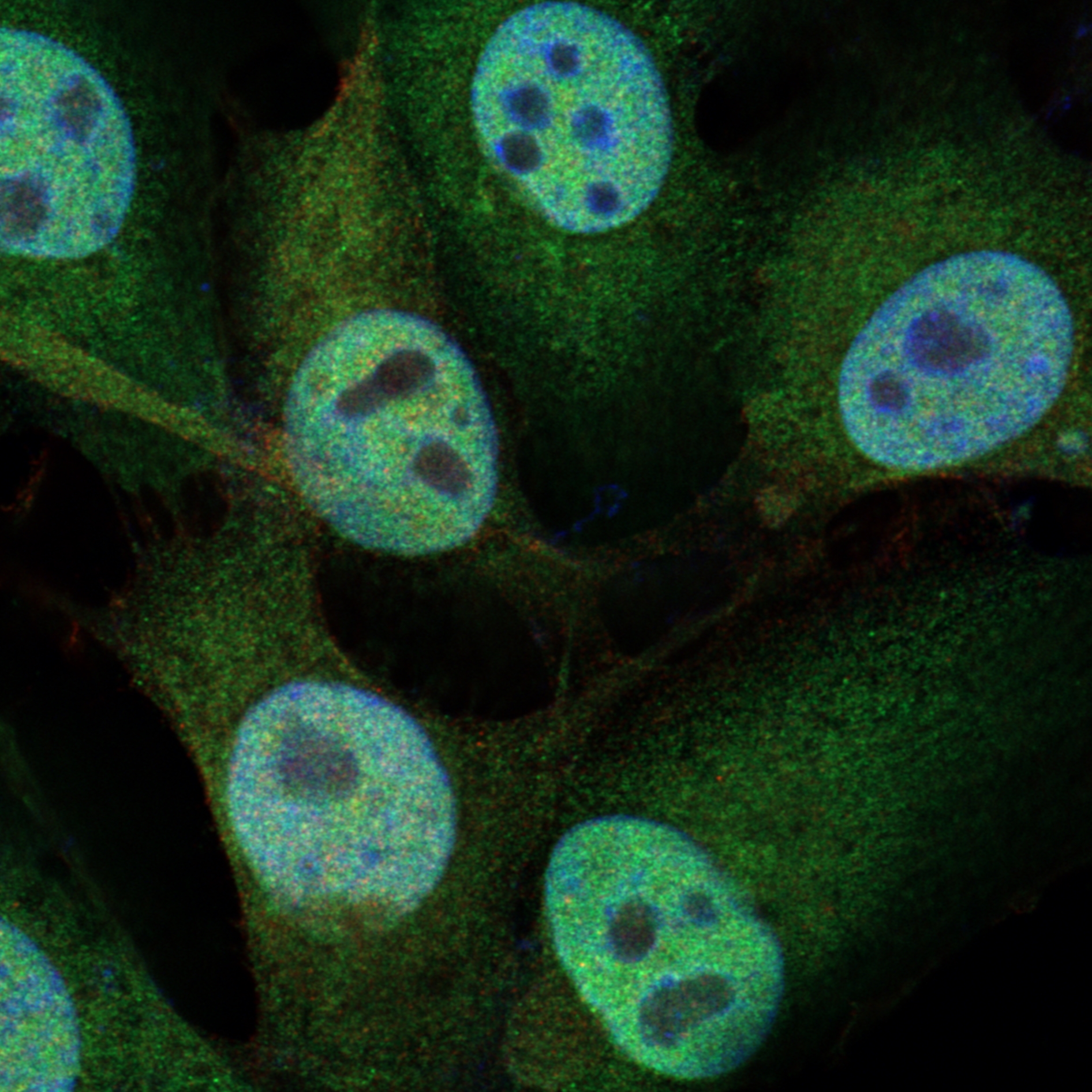

Supplement: Supplementary file 3 — Source data Fig. 2 [file 44318_2024_85_MOESM3_ESM.zip › SD Figure 2/2C high resolution/Serum merge.jpg]

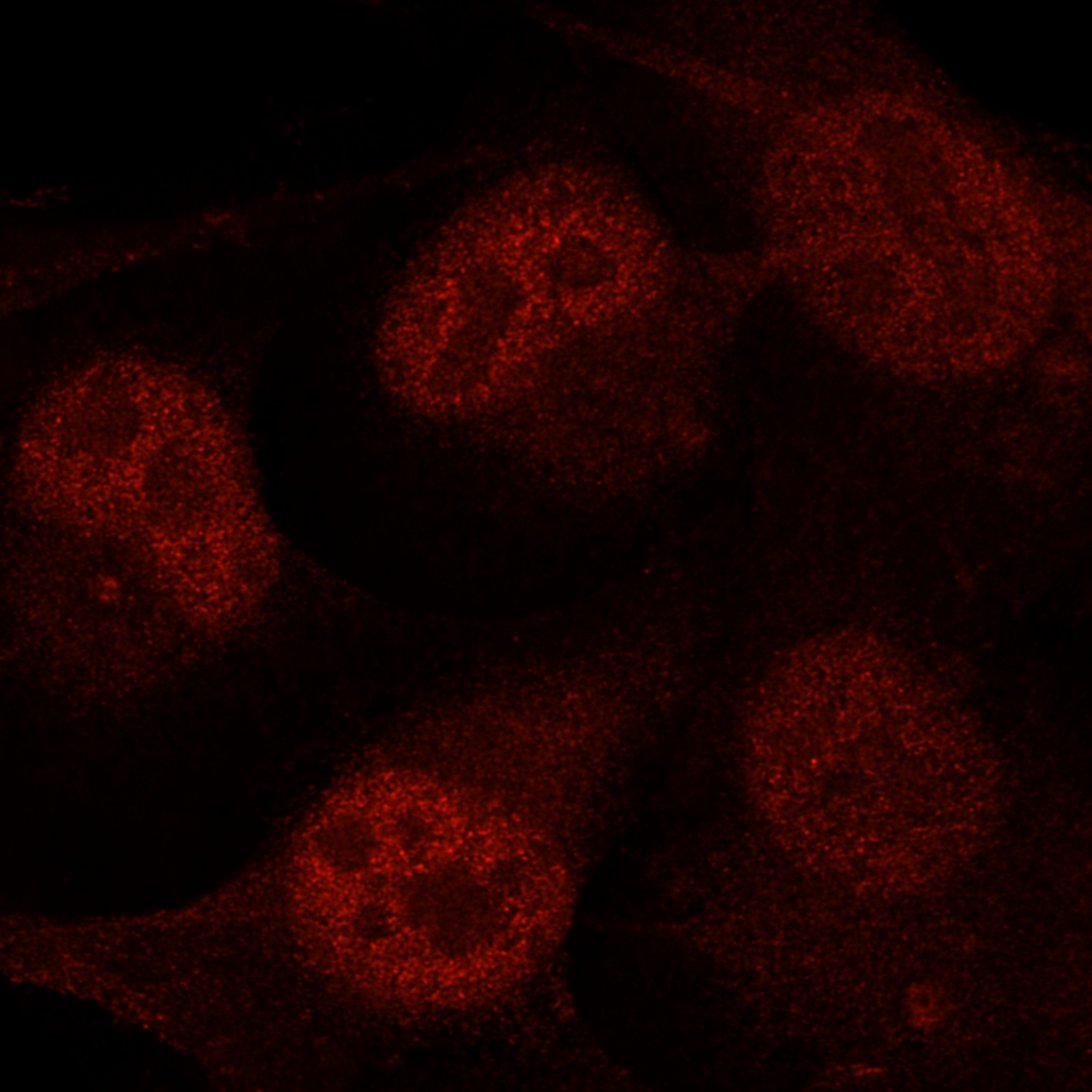

Supplement: Supplementary file 3 — Source data Fig. 2 [file 44318_2024_85_MOESM3_ESM.zip › SD Figure 2/2C high resolution/Free IA.jpg]

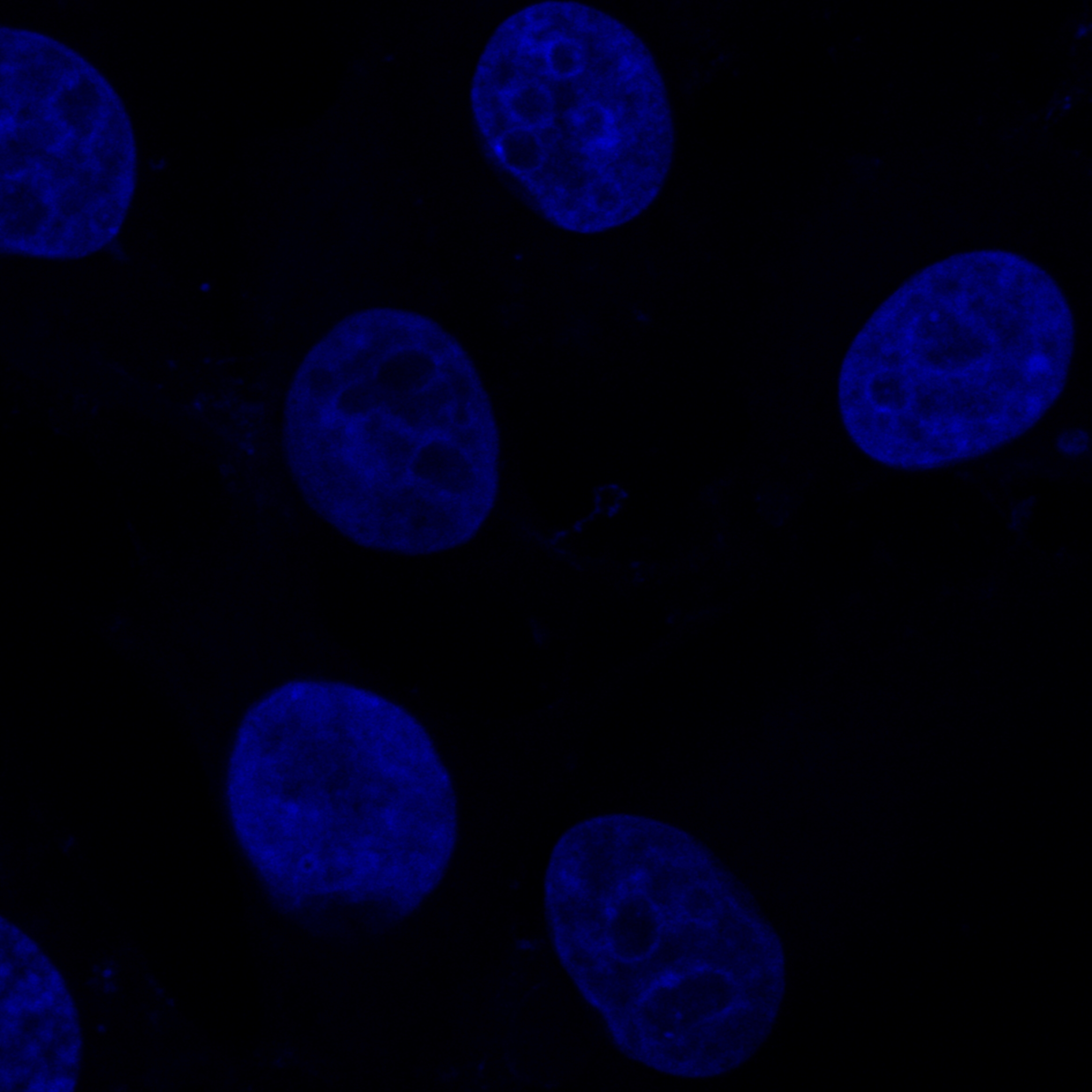

Supplement: Supplementary file 3 — Source data Fig. 2 [file 44318_2024_85_MOESM3_ESM.zip › SD Figure 2/2C high resolution/Serum DAPI.jpg]

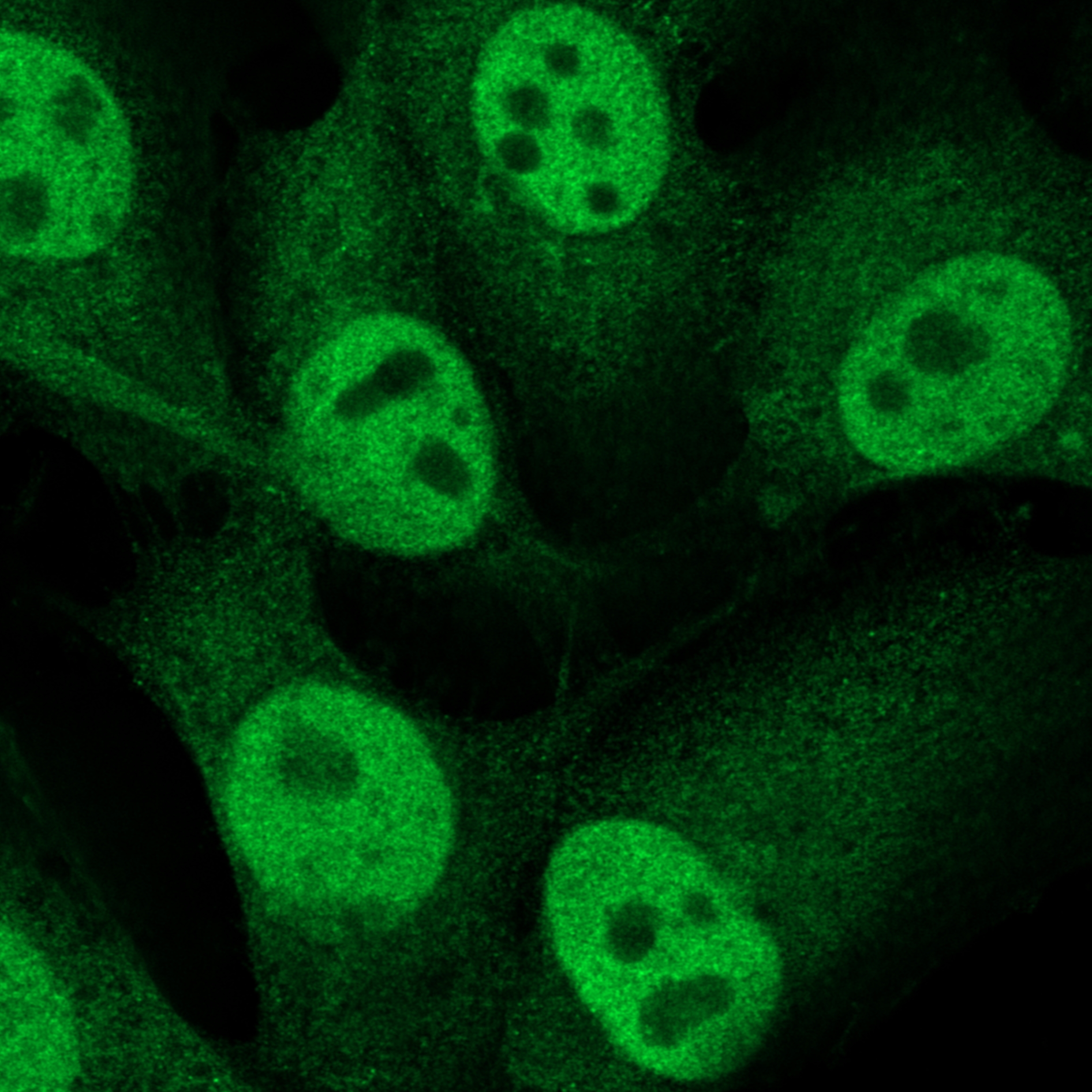

Supplement: Supplementary file 3 — Source data Fig. 2 [file 44318_2024_85_MOESM3_ESM.zip › SD Figure 2/2C high resolution/Serum YAP.jpg]

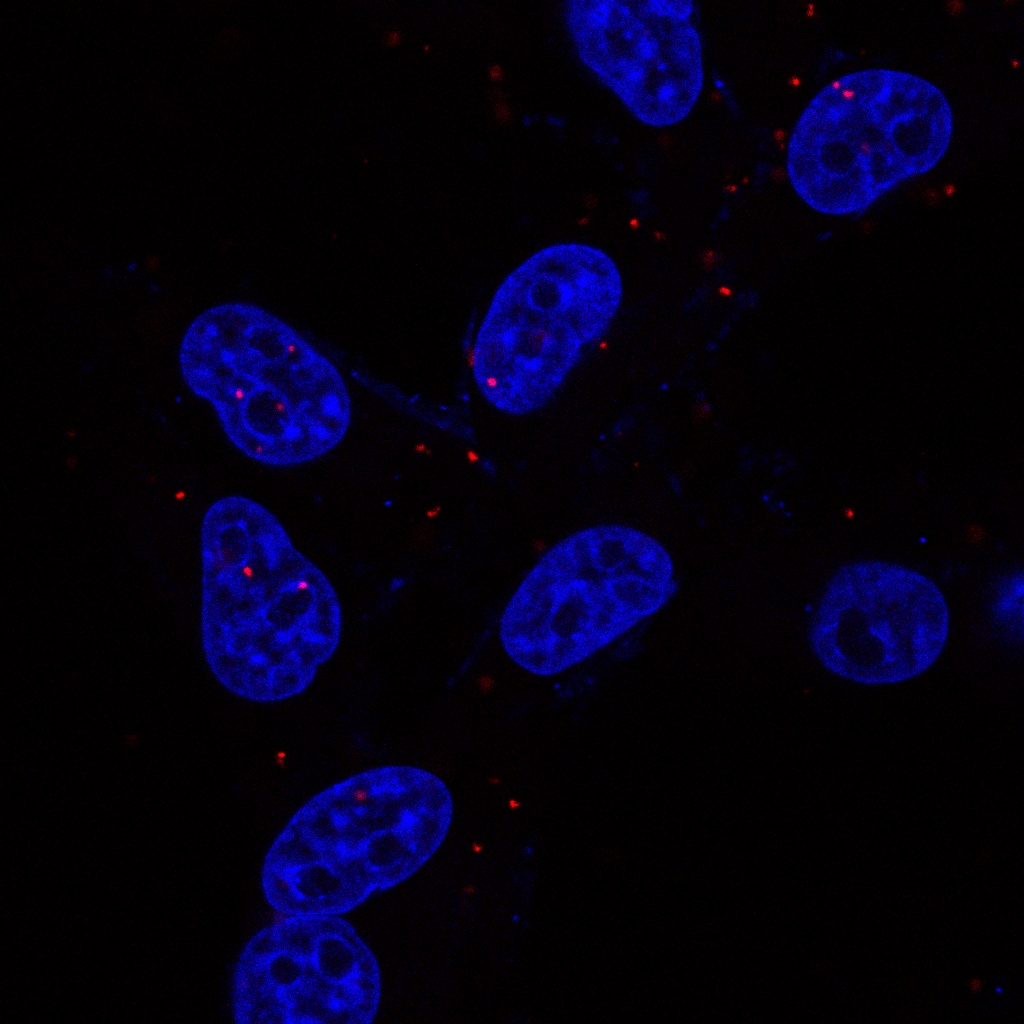

Supplement: Supplementary file 3 — Source data Fig. 2 [file 44318_2024_85_MOESM3_ESM.zip › SD Figure 2/2A high resolution/Free merge.jpg]

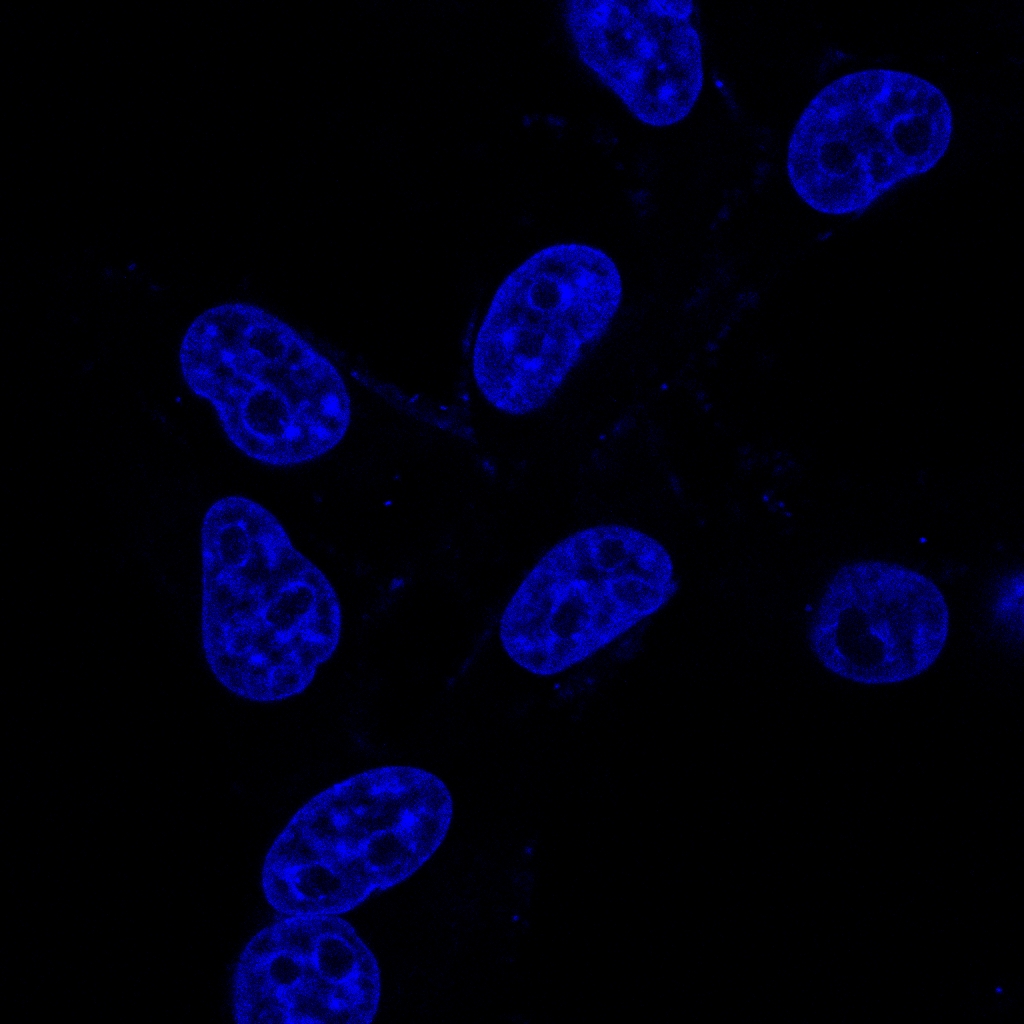

Supplement: Supplementary file 3 — Source data Fig. 2 [file 44318_2024_85_MOESM3_ESM.zip › SD Figure 2/2A high resolution/Free DAPI.jpg]

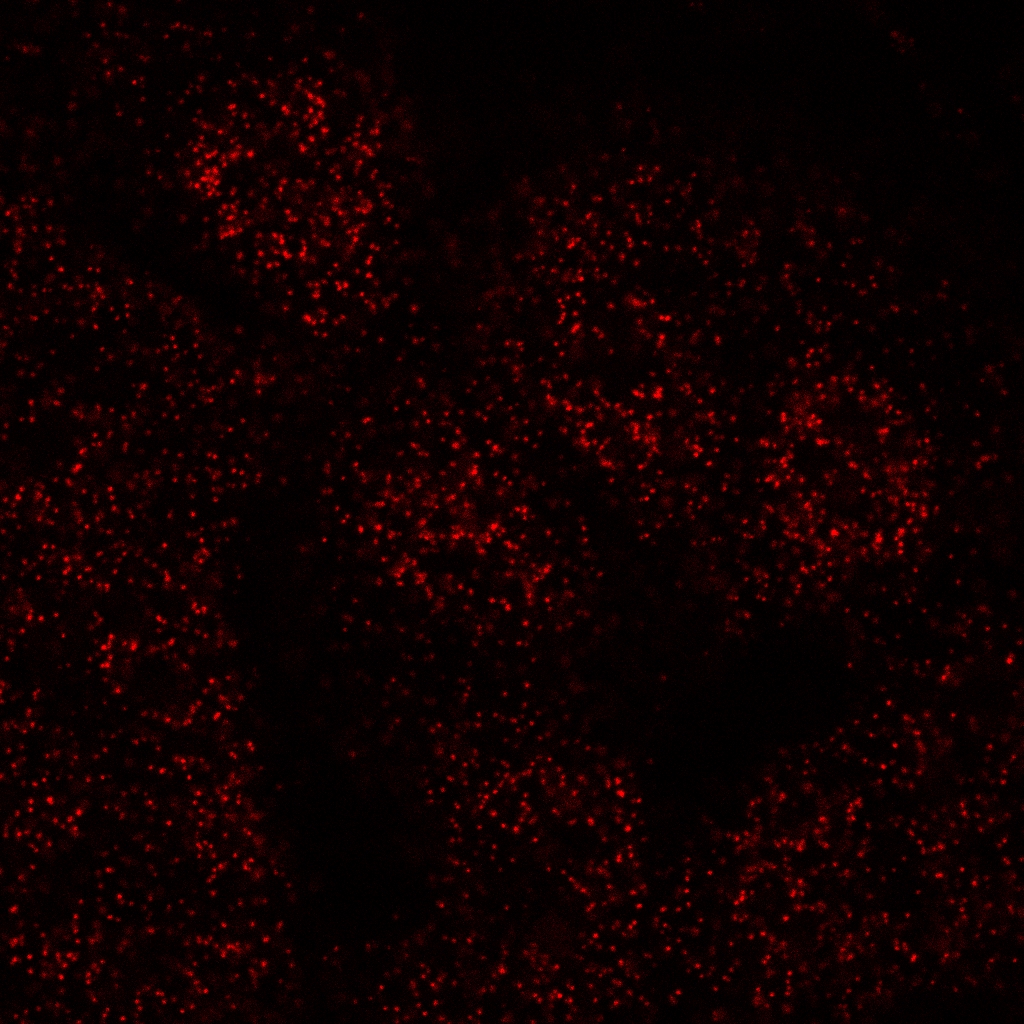

Supplement: Supplementary file 3 — Source data Fig. 2 [file 44318_2024_85_MOESM3_ESM.zip › SD Figure 2/2A high resolution/Serum PLA.jpg]

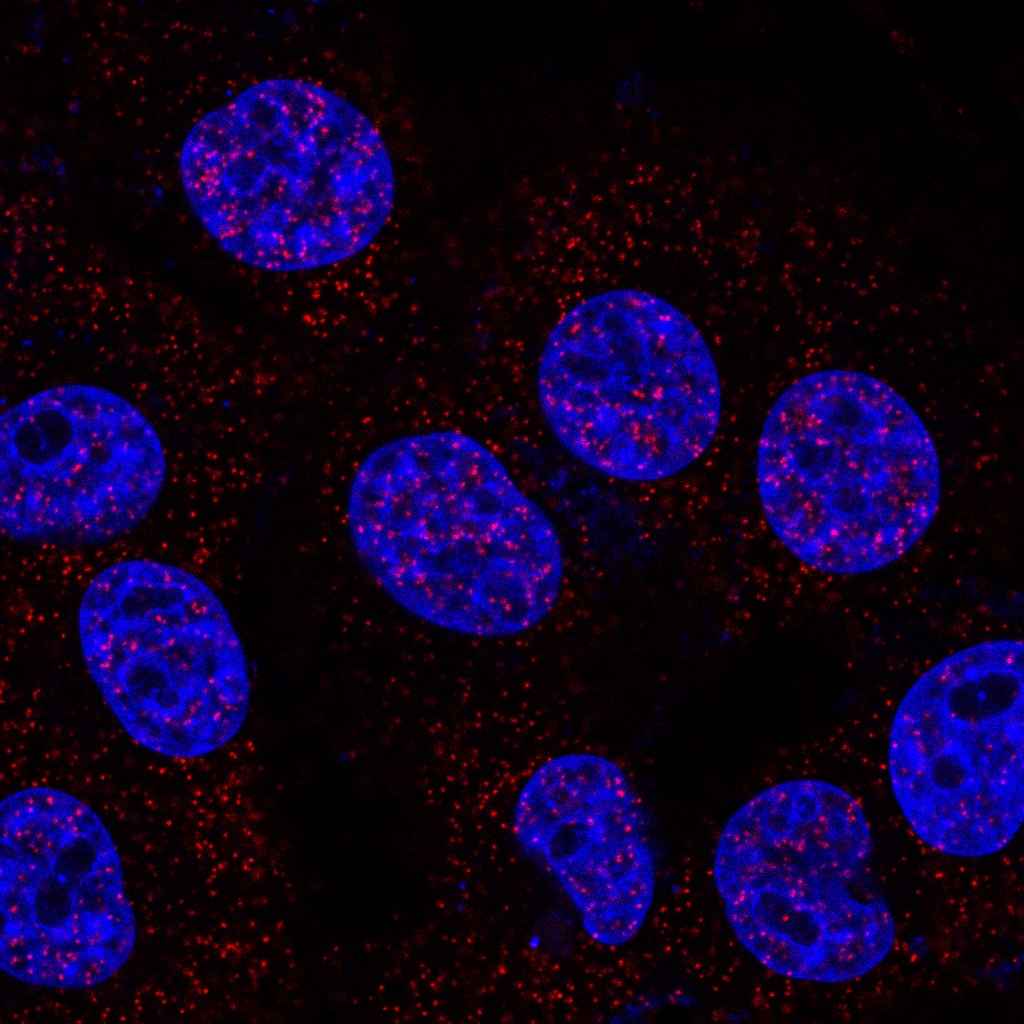

Supplement: Supplementary file 3 — Source data Fig. 2 [file 44318_2024_85_MOESM3_ESM.zip › SD Figure 2/2A high resolution/Serum merge.jpg]

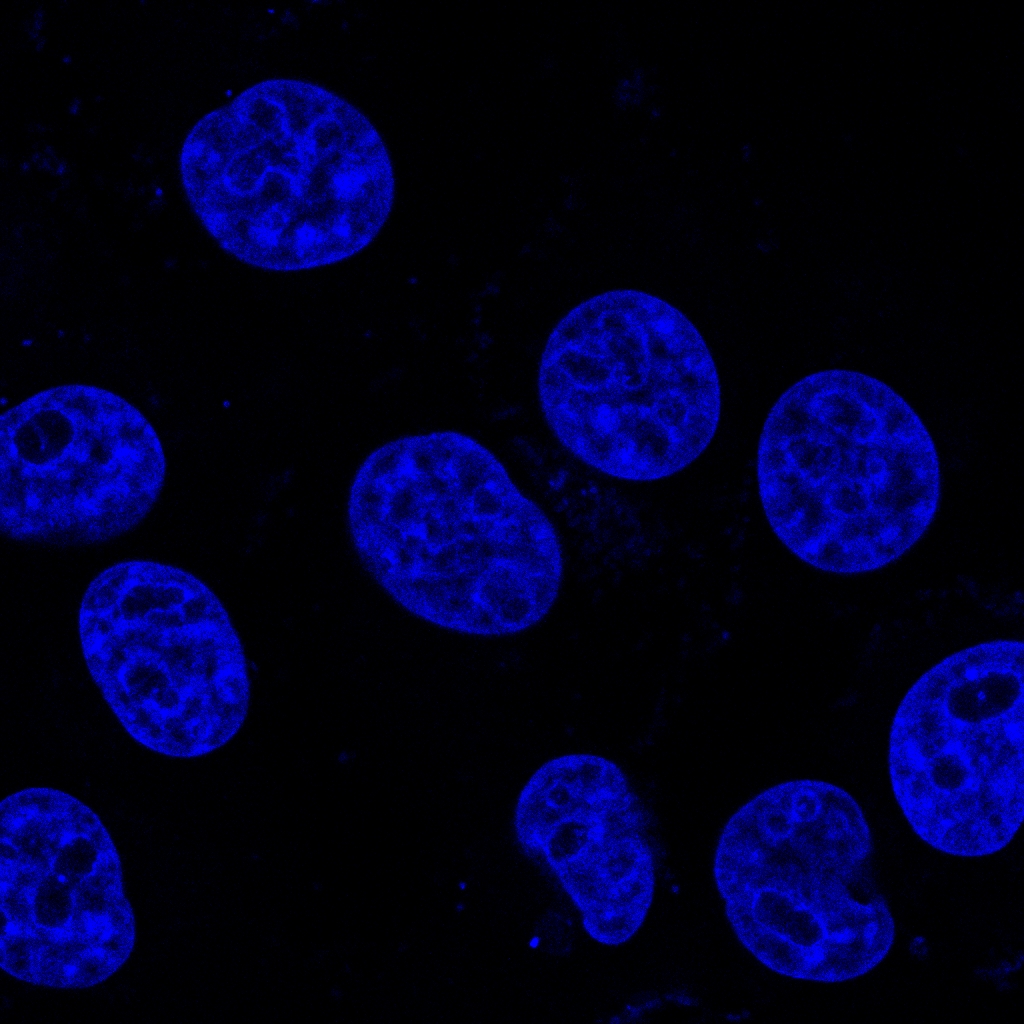

Supplement: Supplementary file 3 — Source data Fig. 2 [file 44318_2024_85_MOESM3_ESM.zip › SD Figure 2/2A high resolution/Serum DAPI.jpg]

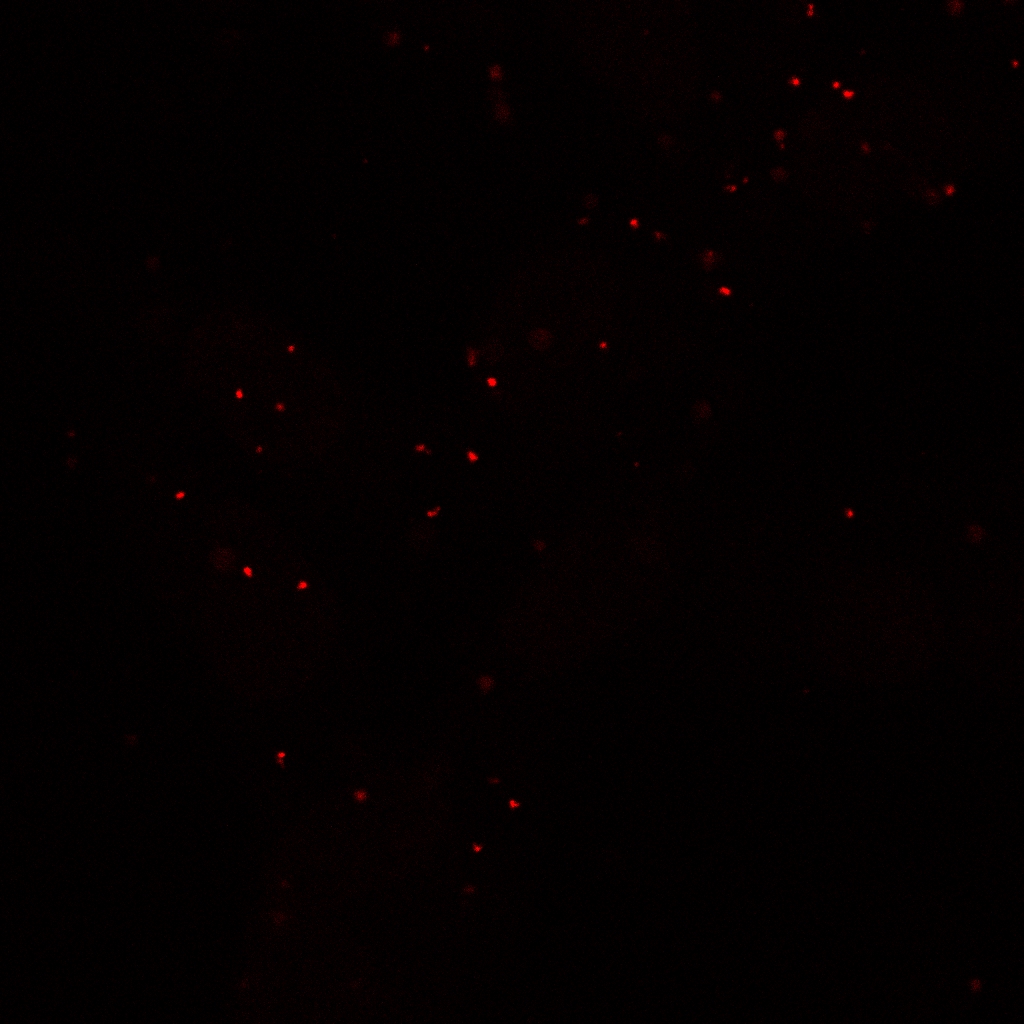

Supplement: Supplementary file 3 — Source data Fig. 2 [file 44318_2024_85_MOESM3_ESM.zip › SD Figure 2/2A high resolution/Free PLA.jpg]

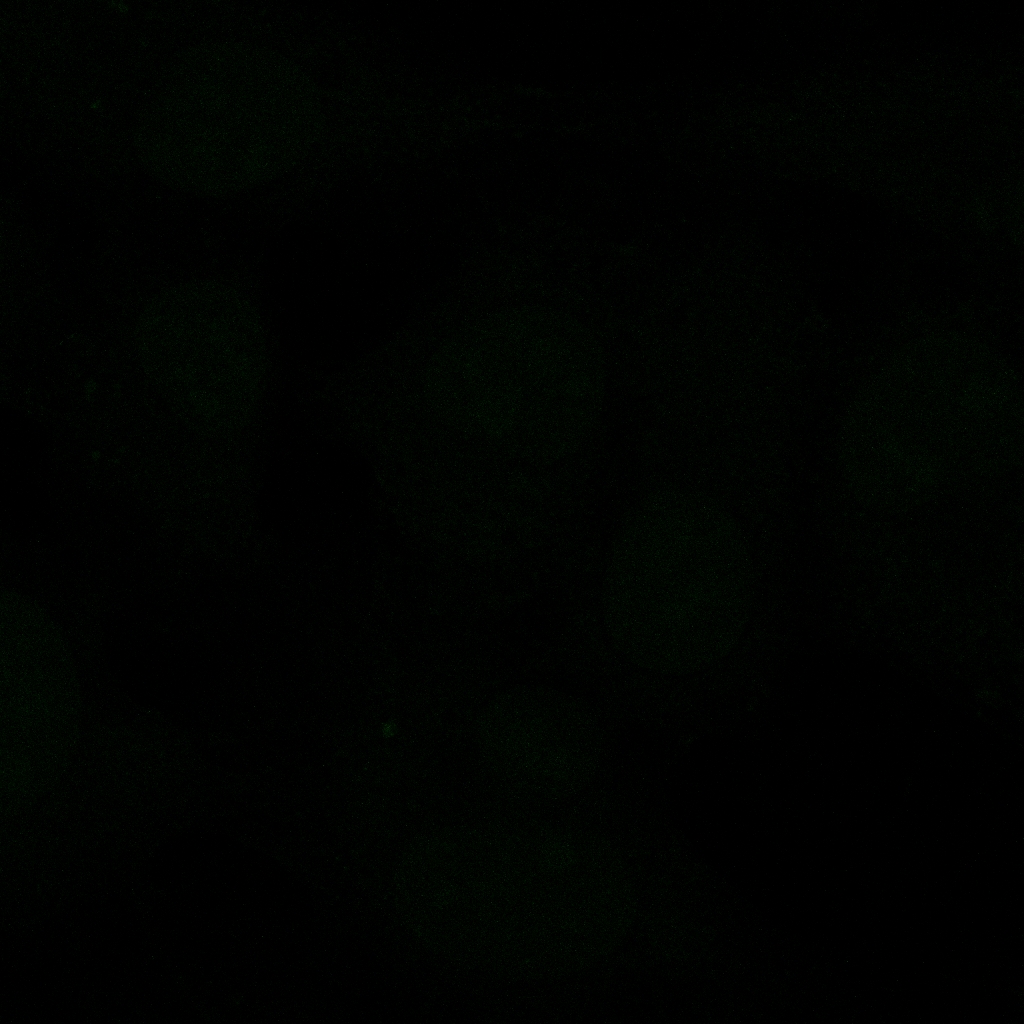

Supplement: Supplementary file 4 — Source data Fig. 3 [file 44318_2024_85_MOESM4_ESM.zip › SD Figure 3/3C high resolution/IA KO PLA.jpg]

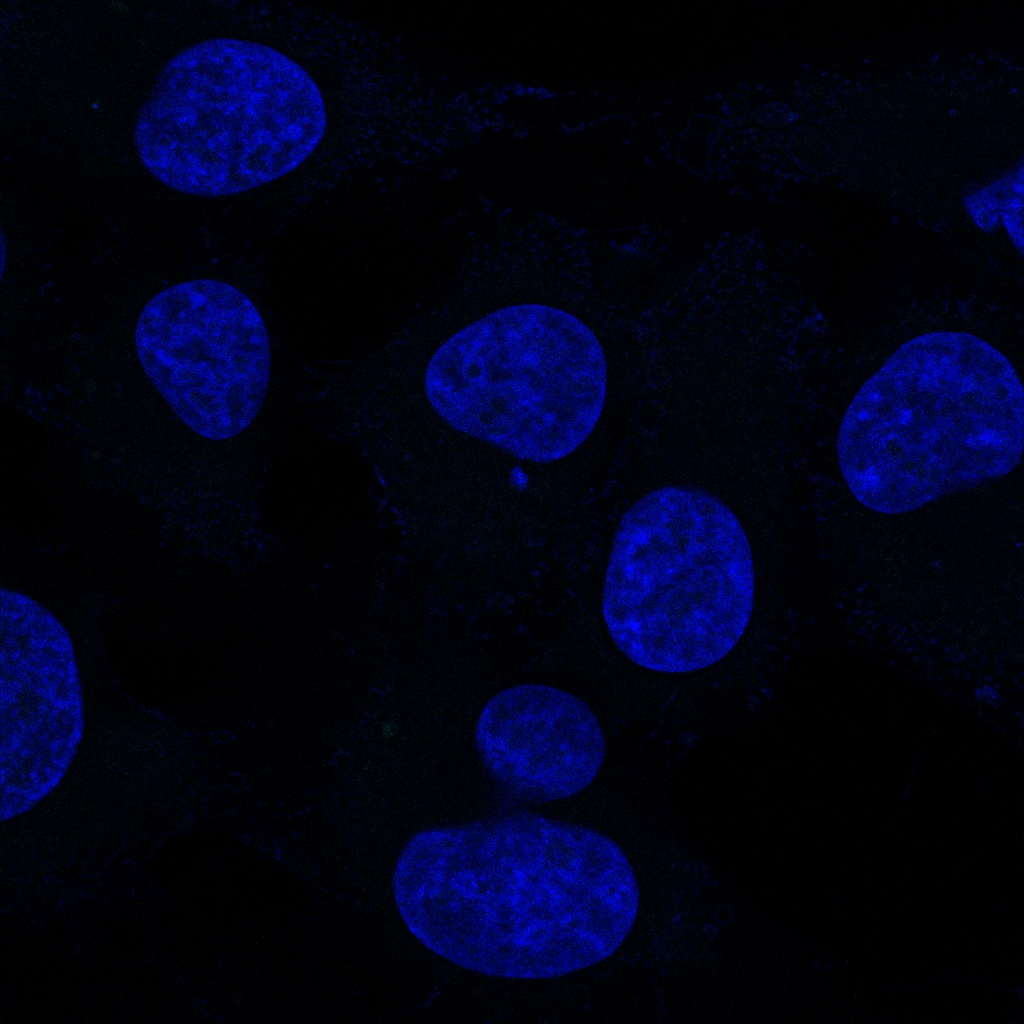

Supplement: Supplementary file 4 — Source data Fig. 3 [file 44318_2024_85_MOESM4_ESM.zip › SD Figure 3/3C high resolution/IA KO merge.jpg]

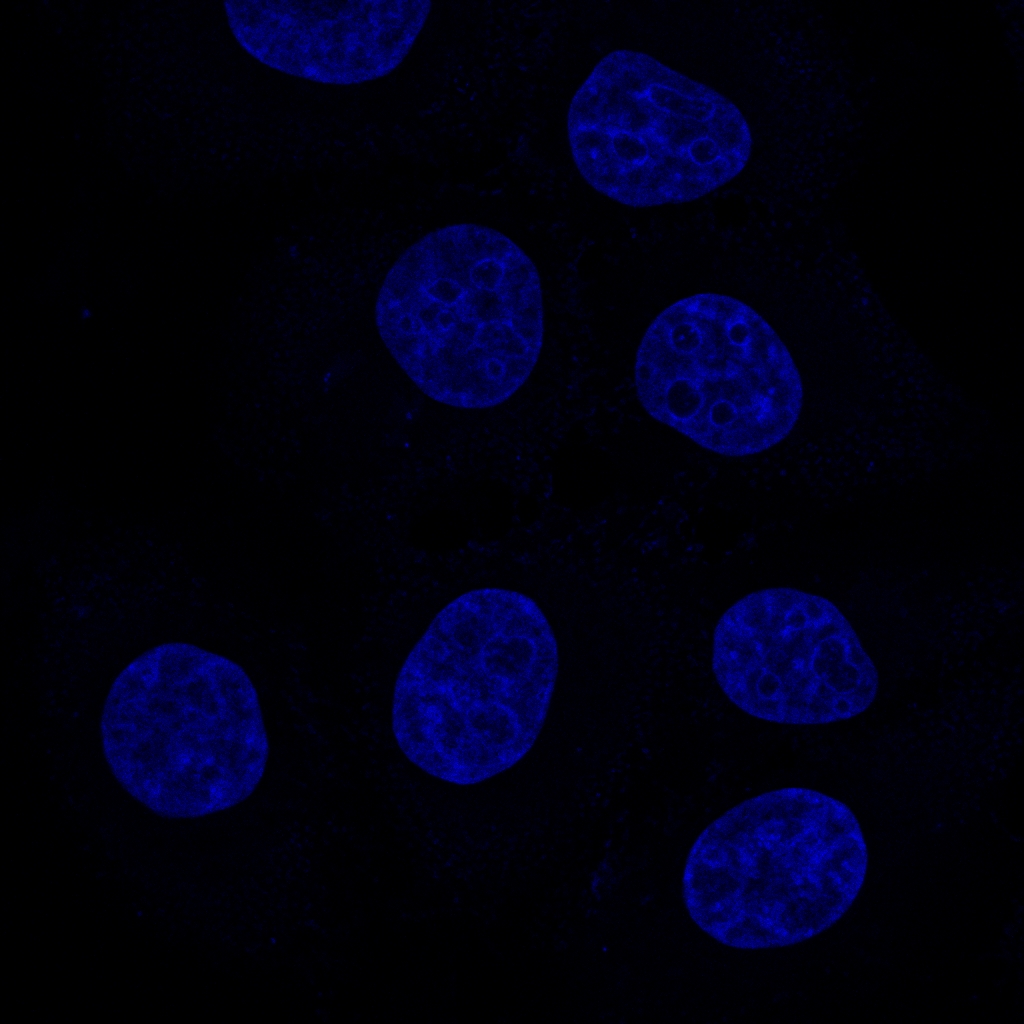

Supplement: Supplementary file 4 — Source data Fig. 3 [file 44318_2024_85_MOESM4_ESM.zip › SD Figure 3/3C high resolution/IPMK KO DAPI.jpg]

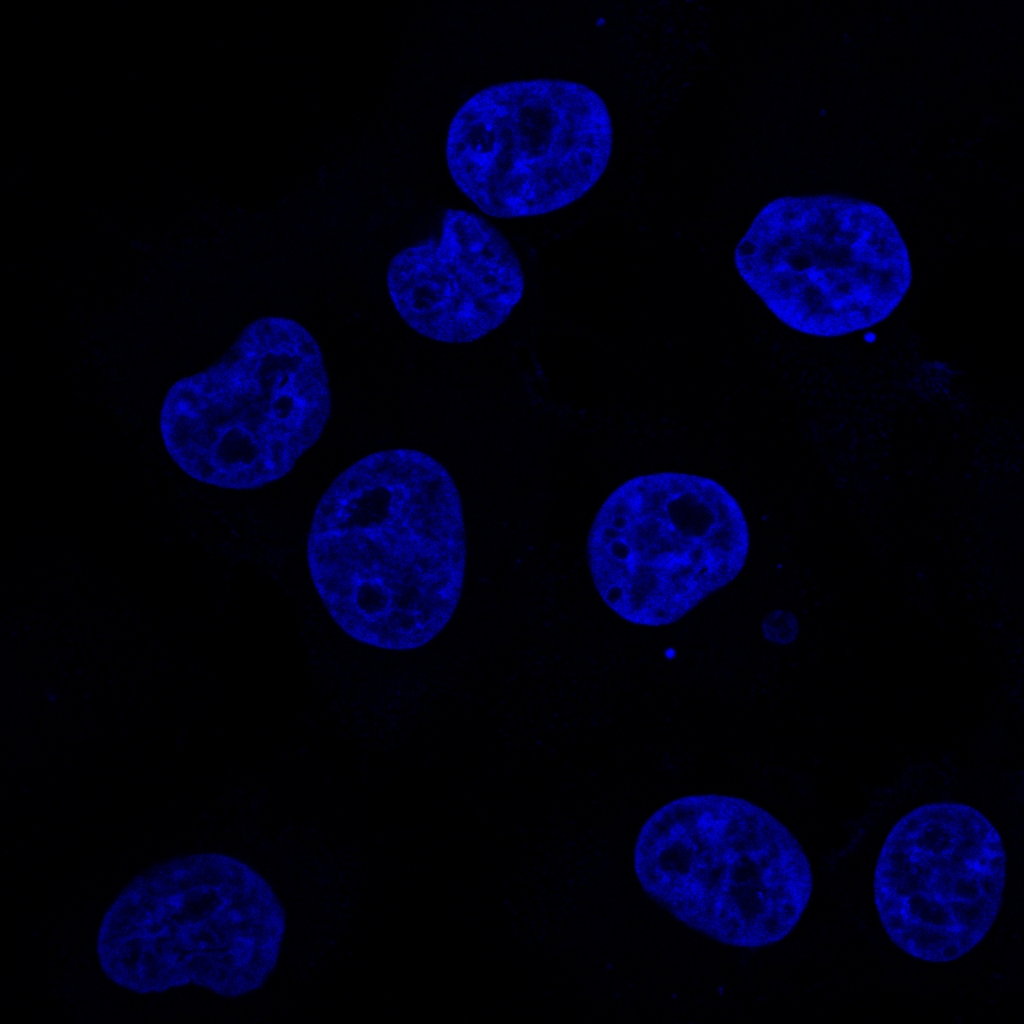

Supplement: Supplementary file 4 — Source data Fig. 3 [file 44318_2024_85_MOESM4_ESM.zip › SD Figure 3/3C high resolution/WT DAPI.jpg]

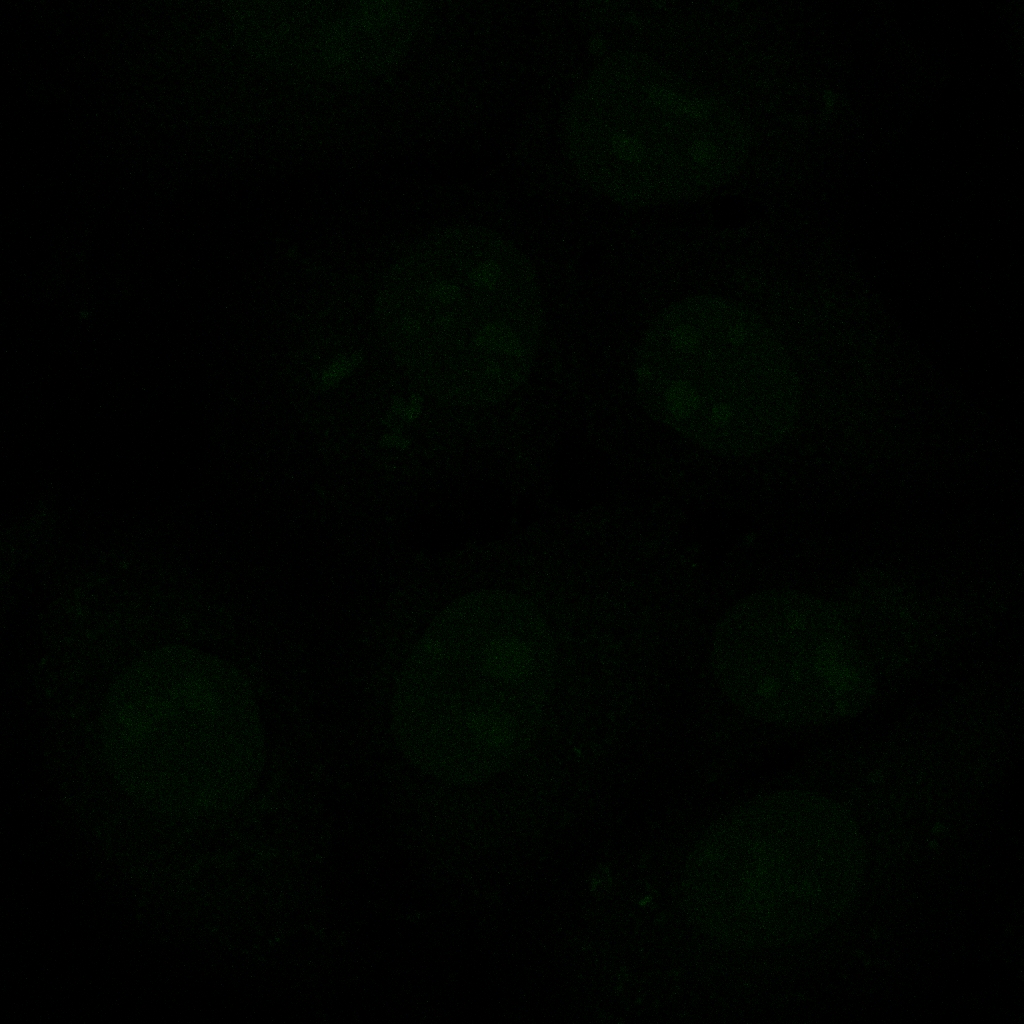

Supplement: Supplementary file 4 — Source data Fig. 3 [file 44318_2024_85_MOESM4_ESM.zip › SD Figure 3/3C high resolution/IPMK KO PLA.jpg]

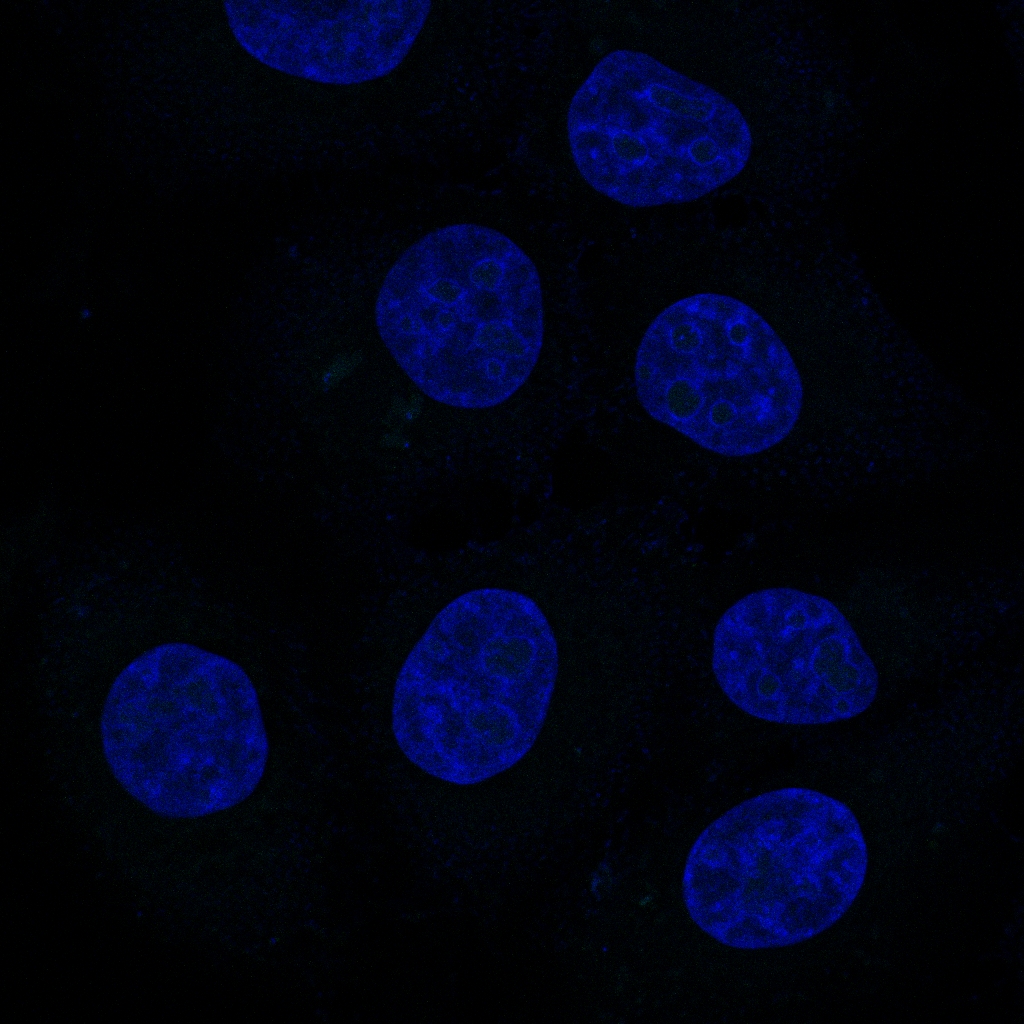

Supplement: Supplementary file 4 — Source data Fig. 3 [file 44318_2024_85_MOESM4_ESM.zip › SD Figure 3/3C high resolution/IPMK KO merge.jpg]

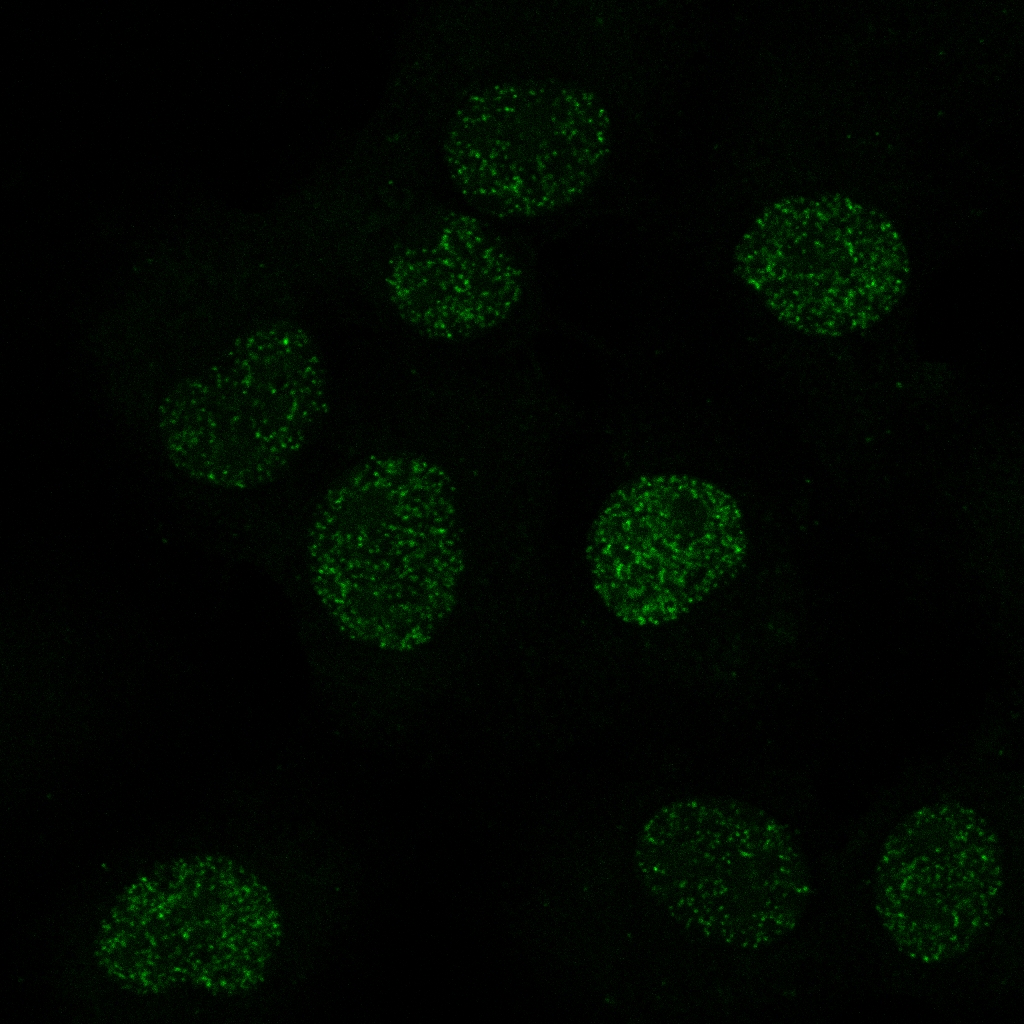

Supplement: Supplementary file 4 — Source data Fig. 3 [file 44318_2024_85_MOESM4_ESM.zip › SD Figure 3/3C high resolution/WT PLA.jpg]

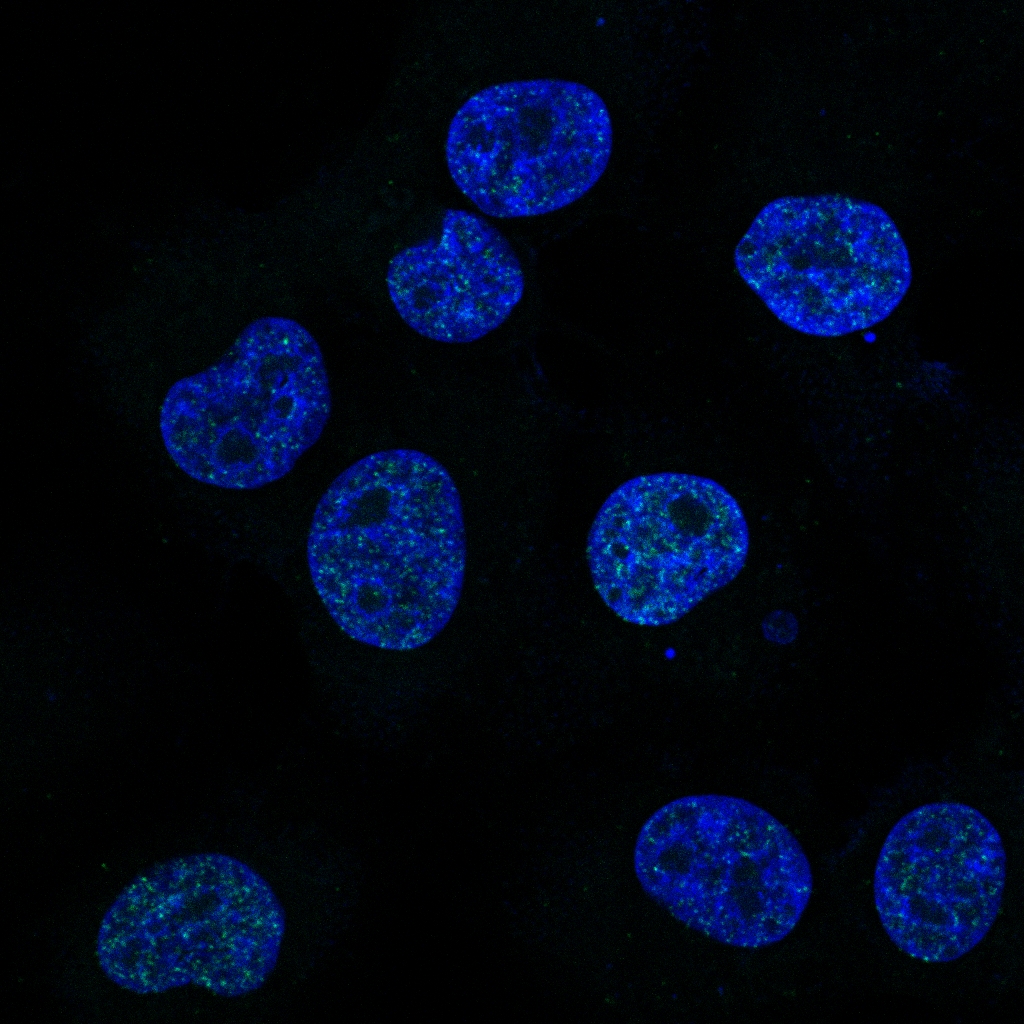

Supplement: Supplementary file 4 — Source data Fig. 3 [file 44318_2024_85_MOESM4_ESM.zip › SD Figure 3/3C high resolution/WT merge.jpg]

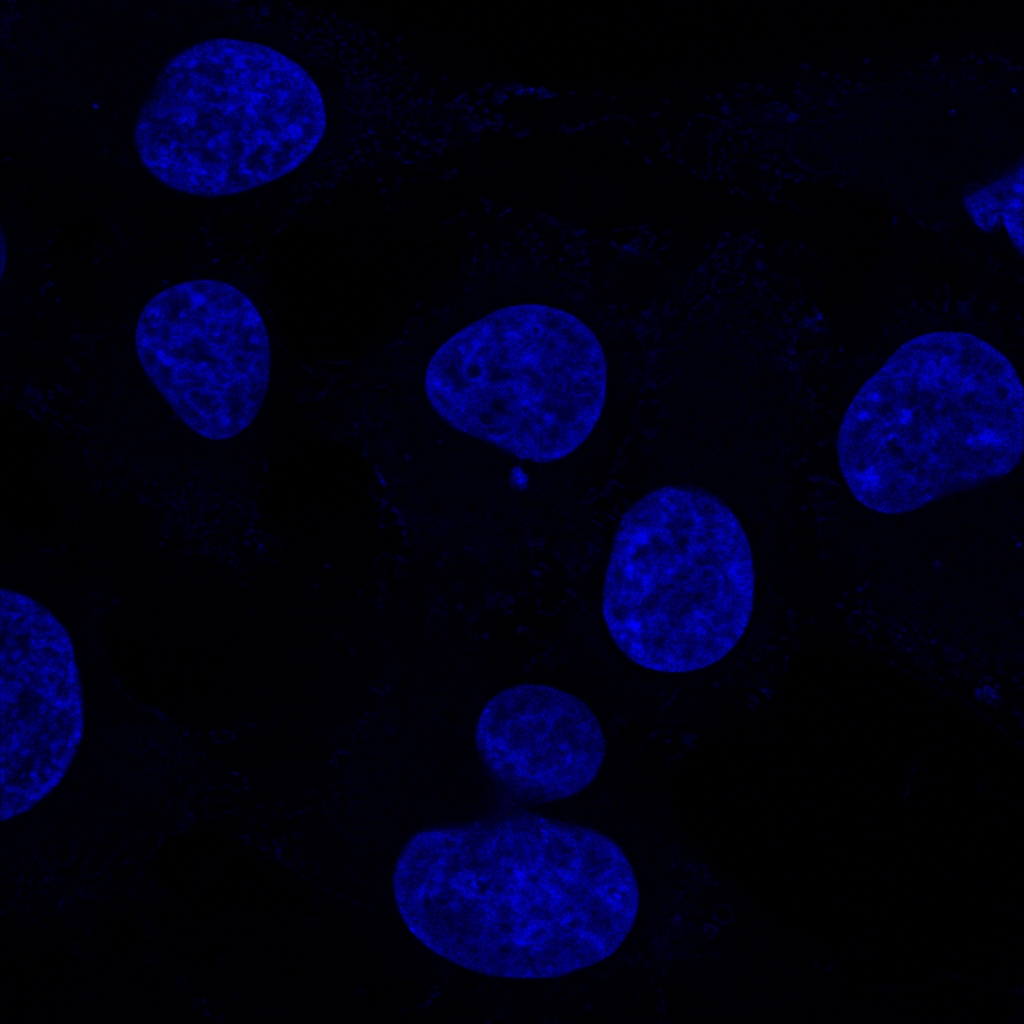

Supplement: Supplementary file 4 — Source data Fig. 3 [file 44318_2024_85_MOESM4_ESM.zip › SD Figure 3/3C high resolution/IA KO DAPI.jpg]

## Slide 1
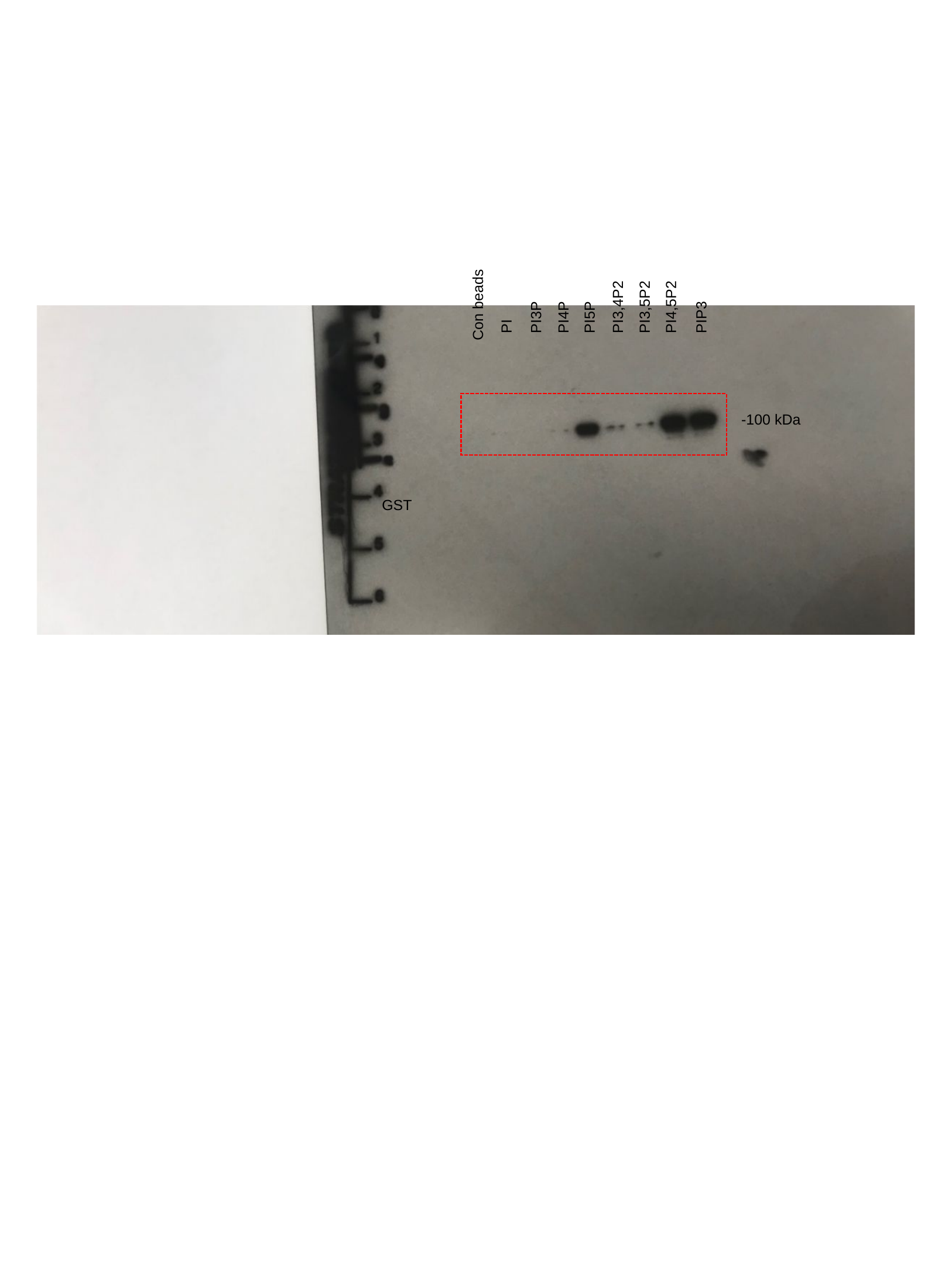

Con beads
PI3,4P2
PI3,5P2
PI4,5P2
PI3P
PI4P
PI5P
PIP3
PI
-100 kDa
-55 kDa
GST

Supplement: Supplementary file 5 — Source data Fig. 4 [file 44318_2024_85_MOESM5_ESM.zip › SD Figure 4/4A.pptx]

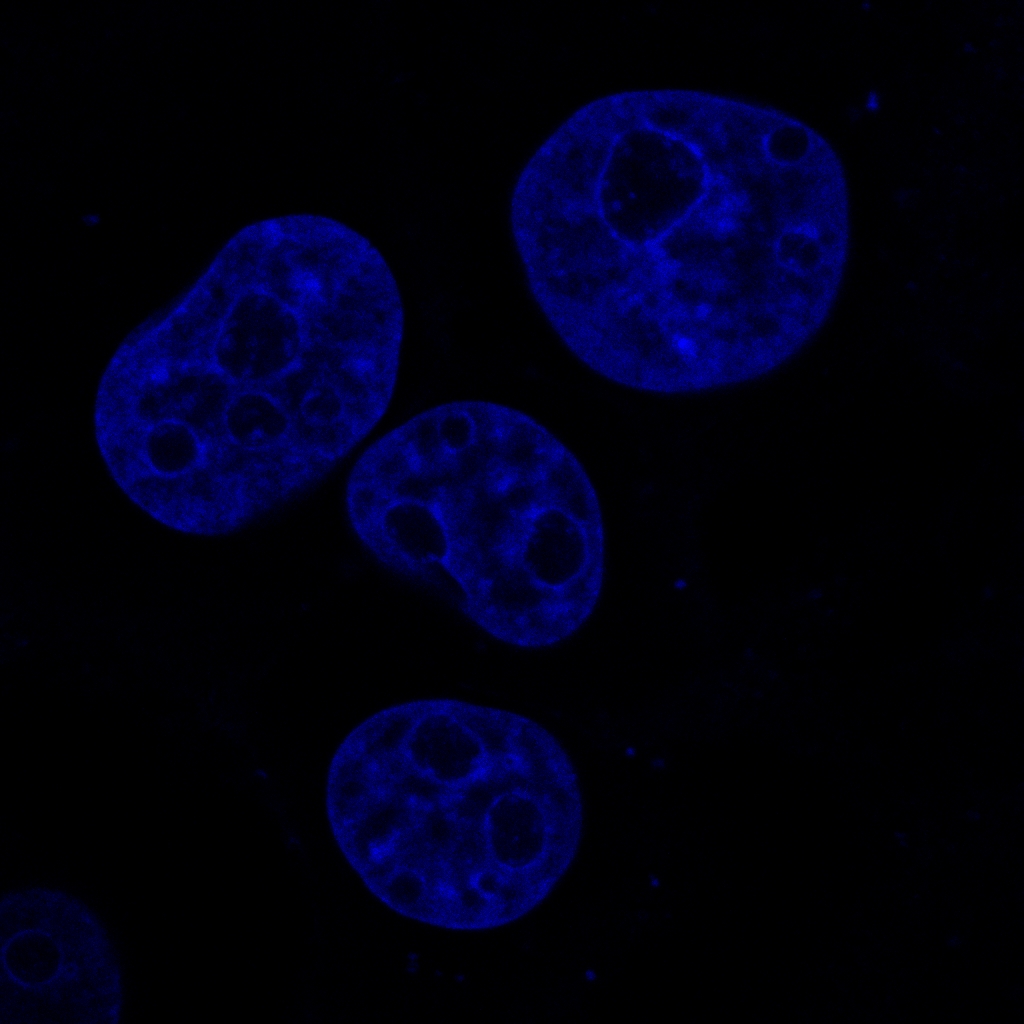

Supplement: Supplementary file 5 — Source data Fig. 4 [file 44318_2024_85_MOESM5_ESM.zip › SD Figure 4/4G high resolution/siIPMK DAPI.jpg]

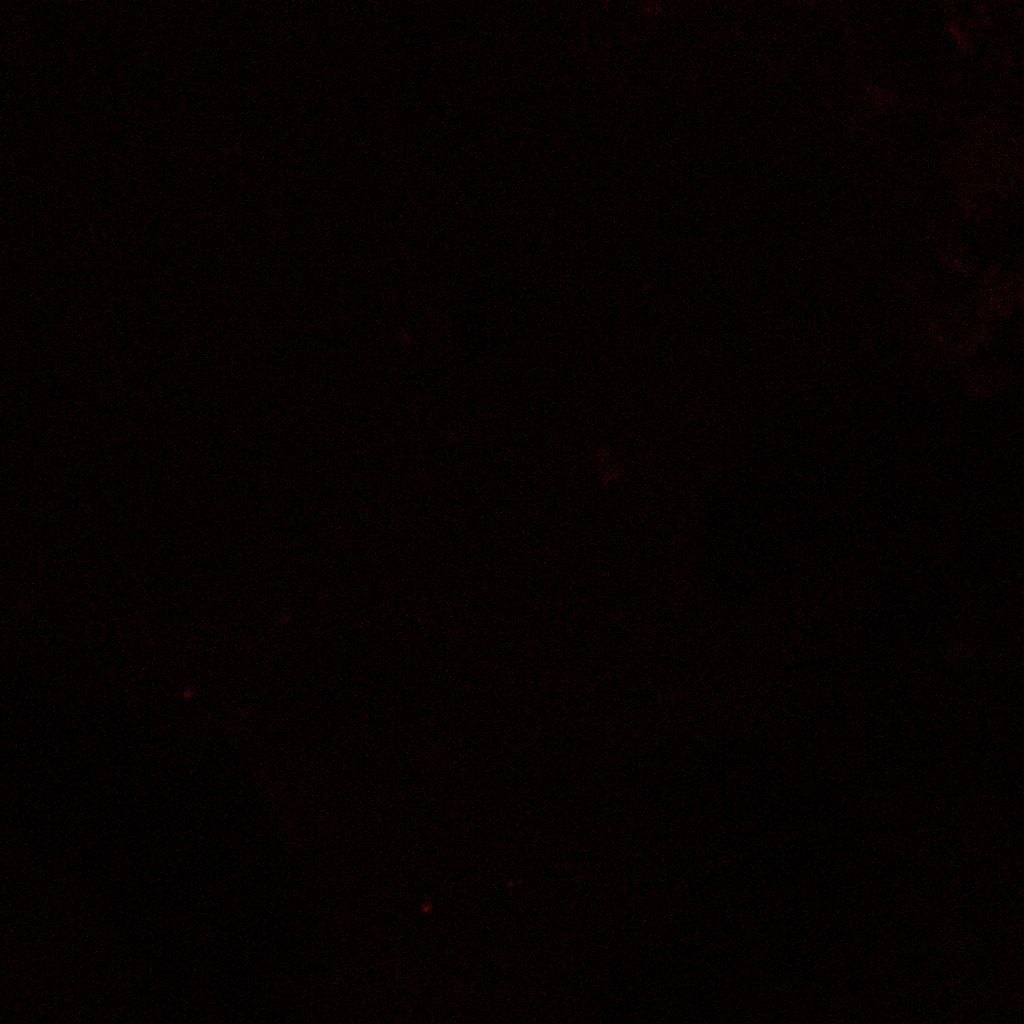

Supplement: Supplementary file 5 — Source data Fig. 4 [file 44318_2024_85_MOESM5_ESM.zip › SD Figure 4/4G high resolution/siIPMK PLA.jpg]

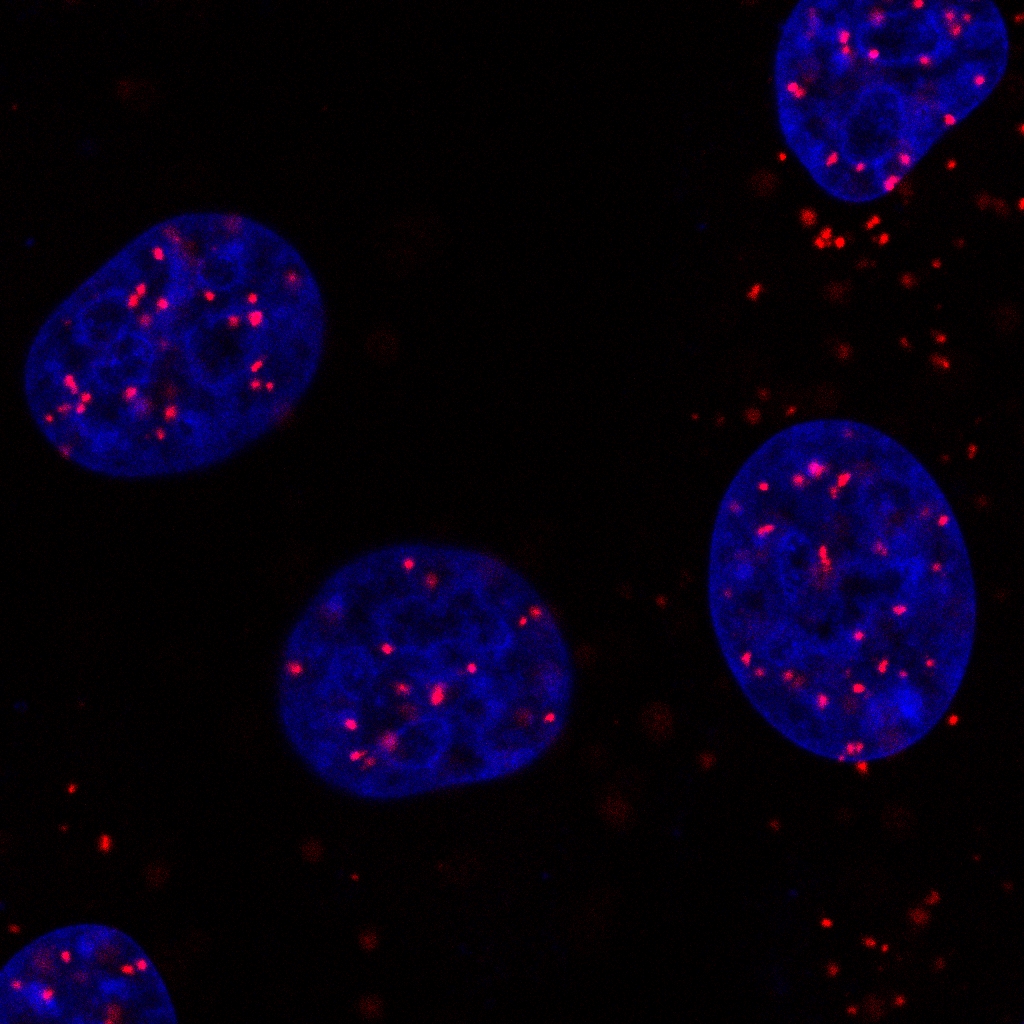

Supplement: Supplementary file 5 — Source data Fig. 4 [file 44318_2024_85_MOESM5_ESM.zip › SD Figure 4/4G high resolution/sicon merge.jpg]

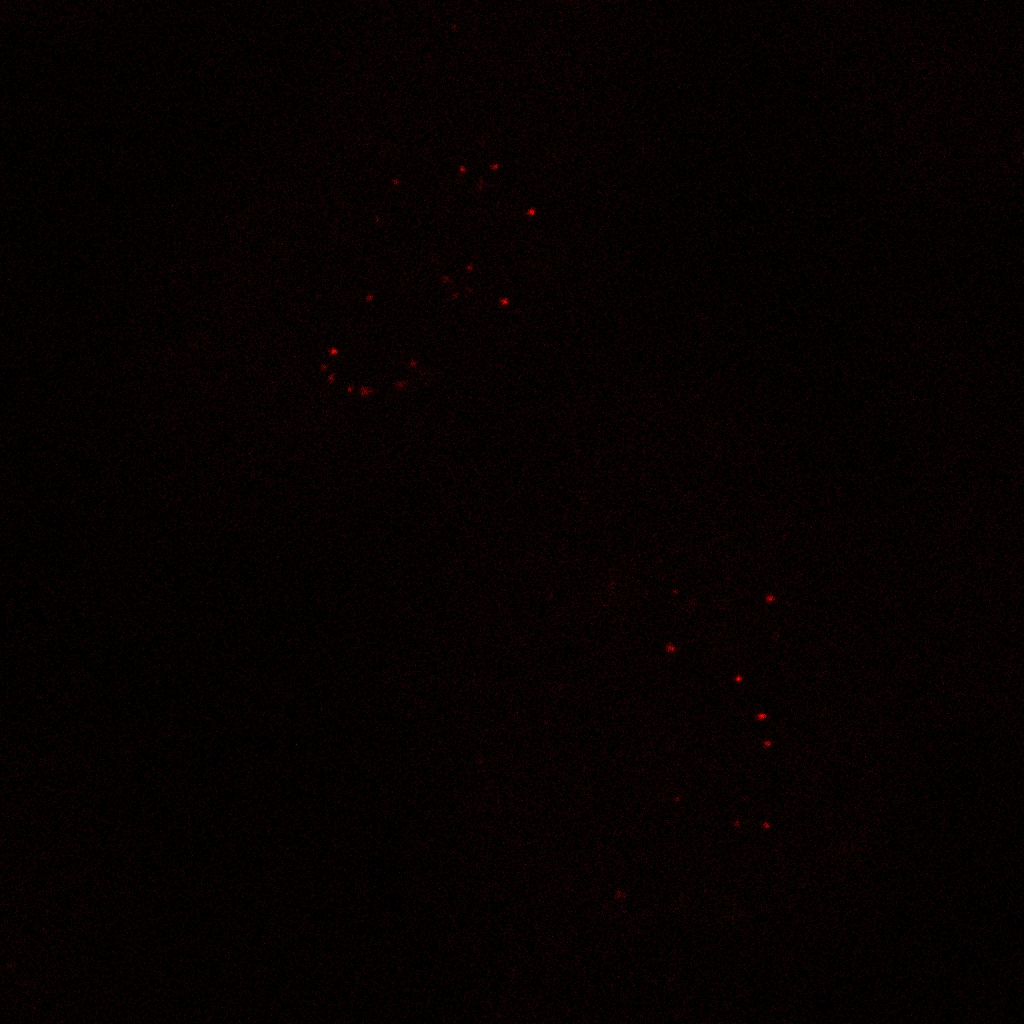

Supplement: Supplementary file 5 — Source data Fig. 4 [file 44318_2024_85_MOESM5_ESM.zip › SD Figure 4/4G high resolution/siIA PLA.jpg]

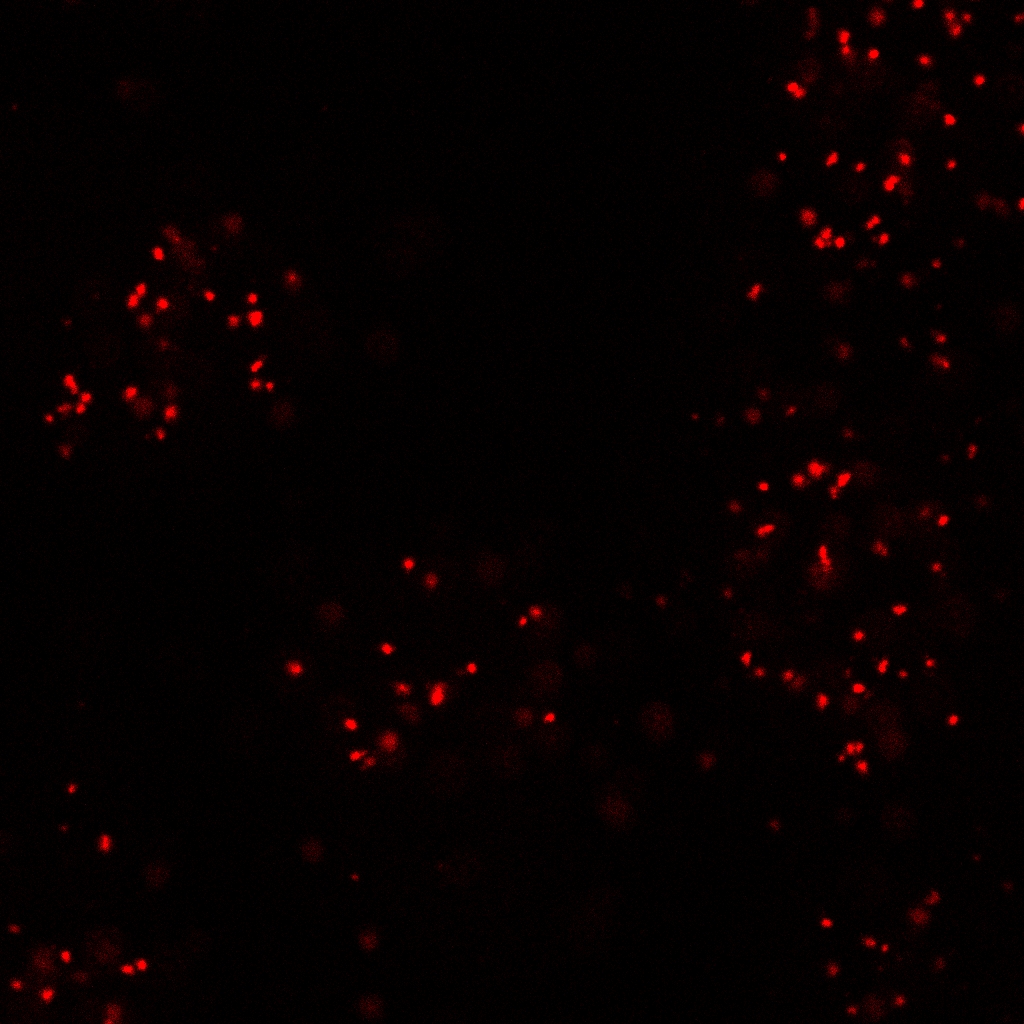

Supplement: Supplementary file 5 — Source data Fig. 4 [file 44318_2024_85_MOESM5_ESM.zip › SD Figure 4/4G high resolution/sicon PLA.jpg]

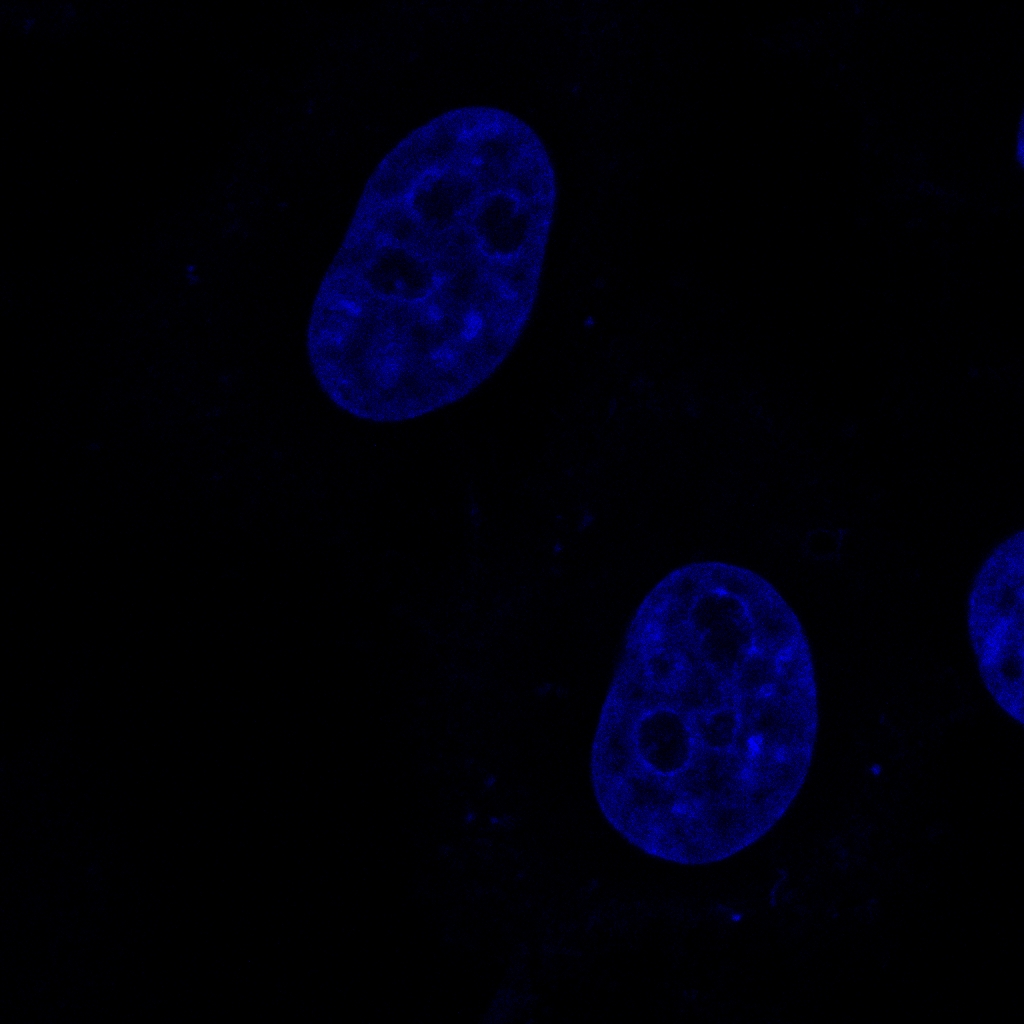

Supplement: Supplementary file 5 — Source data Fig. 4 [file 44318_2024_85_MOESM5_ESM.zip › SD Figure 4/4G high resolution/siIA DAPI.jpg]

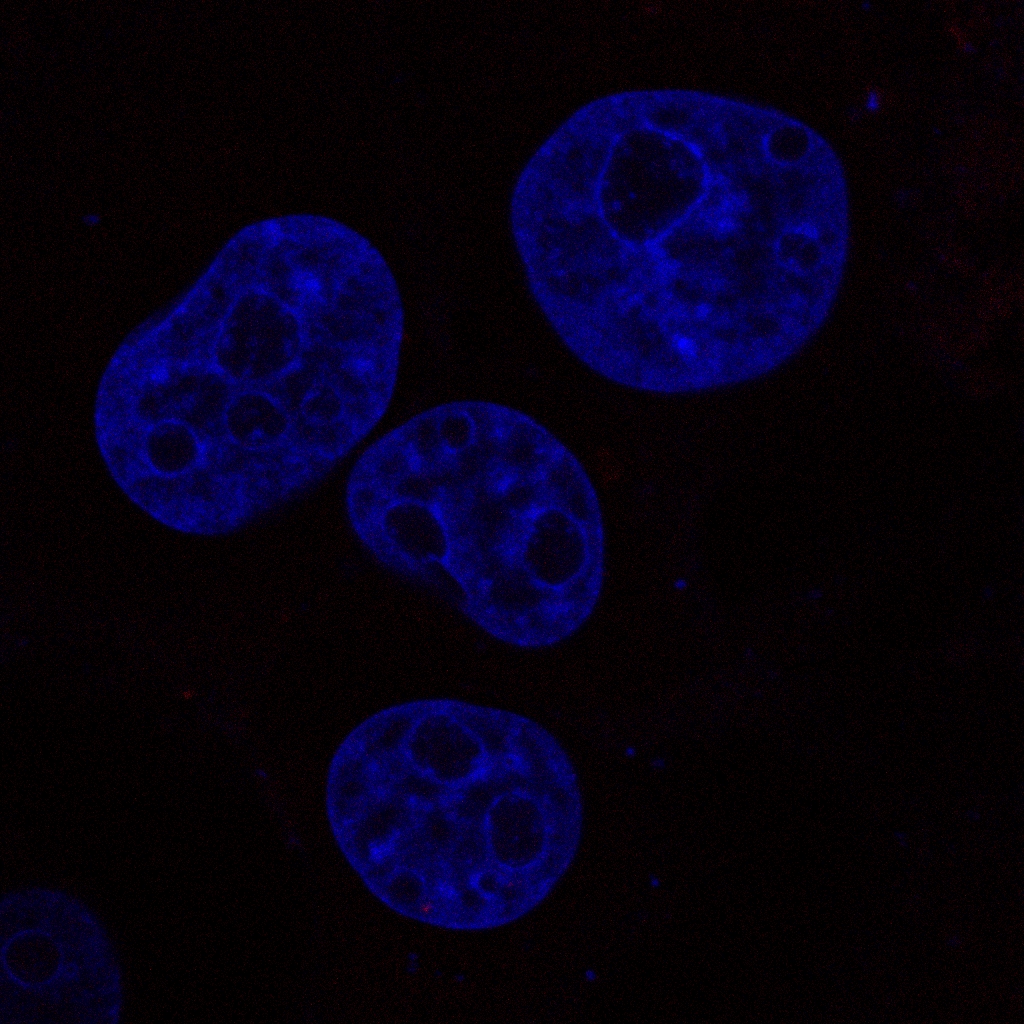

Supplement: Supplementary file 5 — Source data Fig. 4 [file 44318_2024_85_MOESM5_ESM.zip › SD Figure 4/4G high resolution/siIPMK merge.jpg]

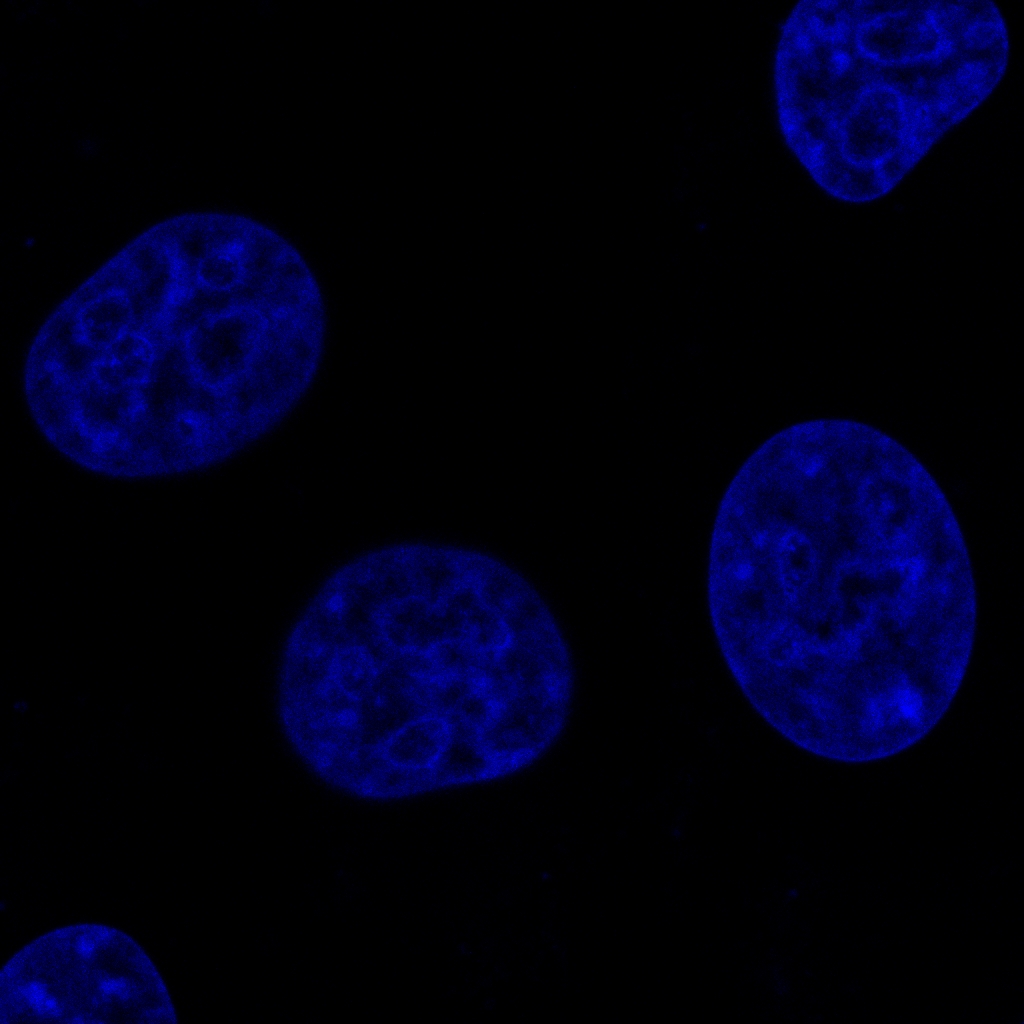

Supplement: Supplementary file 5 — Source data Fig. 4 [file 44318_2024_85_MOESM5_ESM.zip › SD Figure 4/4G high resolution/sicon DAPI.jpg]

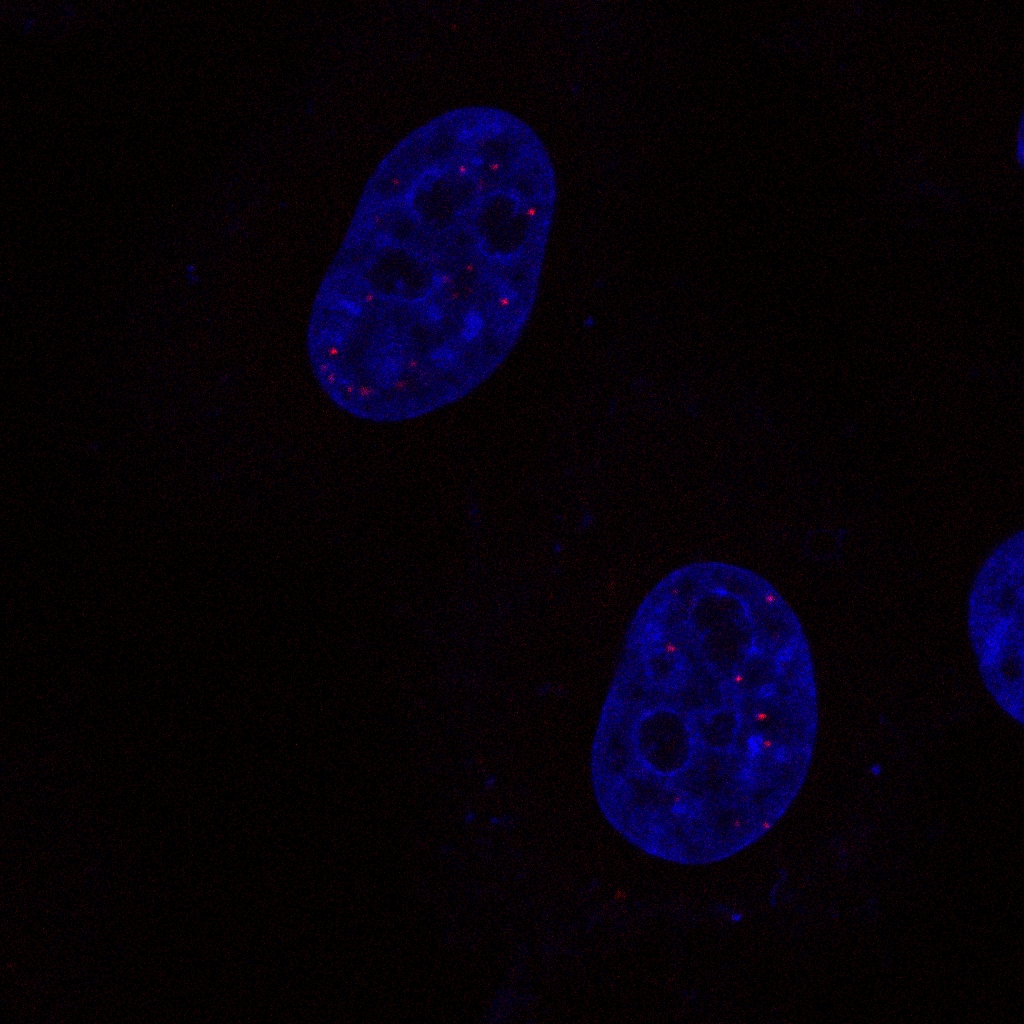

Supplement: Supplementary file 5 — Source data Fig. 4 [file 44318_2024_85_MOESM5_ESM.zip › SD Figure 4/4G high resolution/siIA merge.jpg]

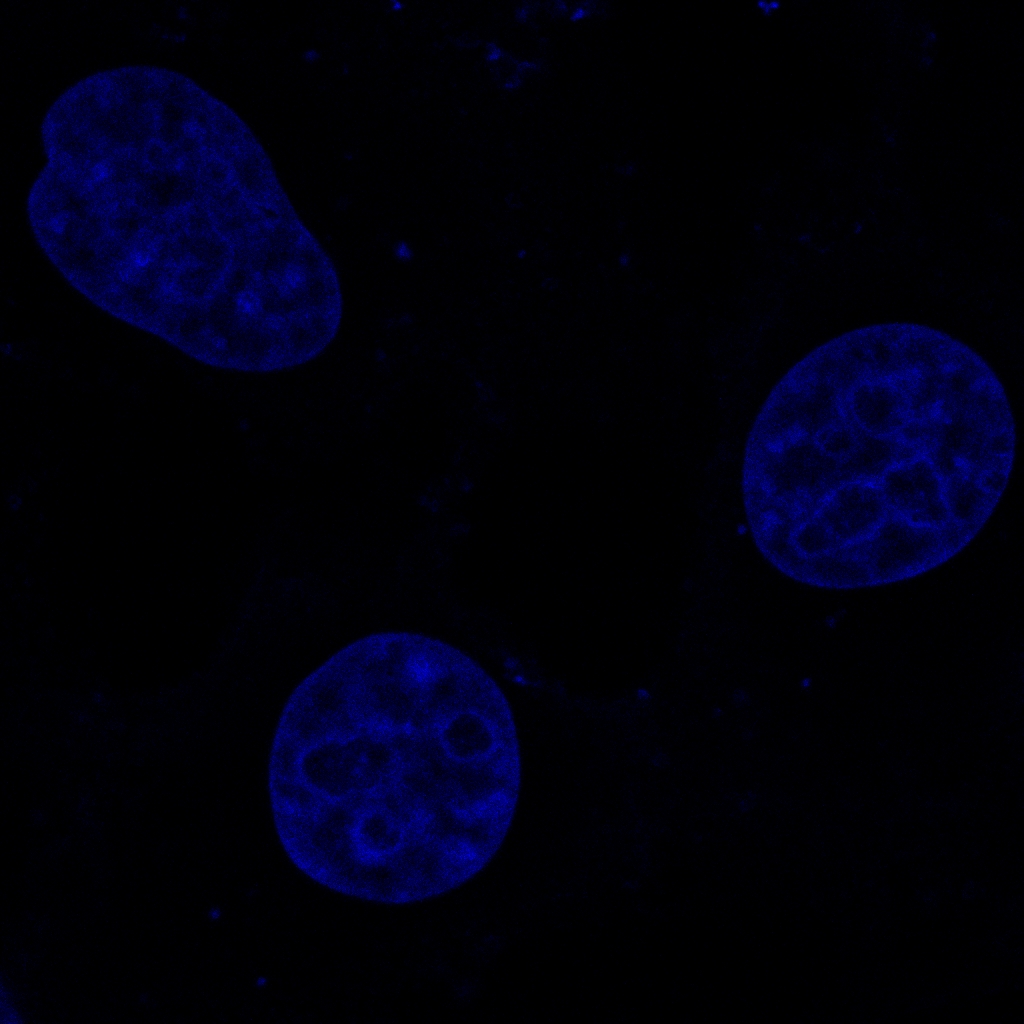

Supplement: Supplementary file 5 — Source data Fig. 4 [file 44318_2024_85_MOESM5_ESM.zip › SD Figure 4/4E high resolution/siIPMK DAPI.jpg]

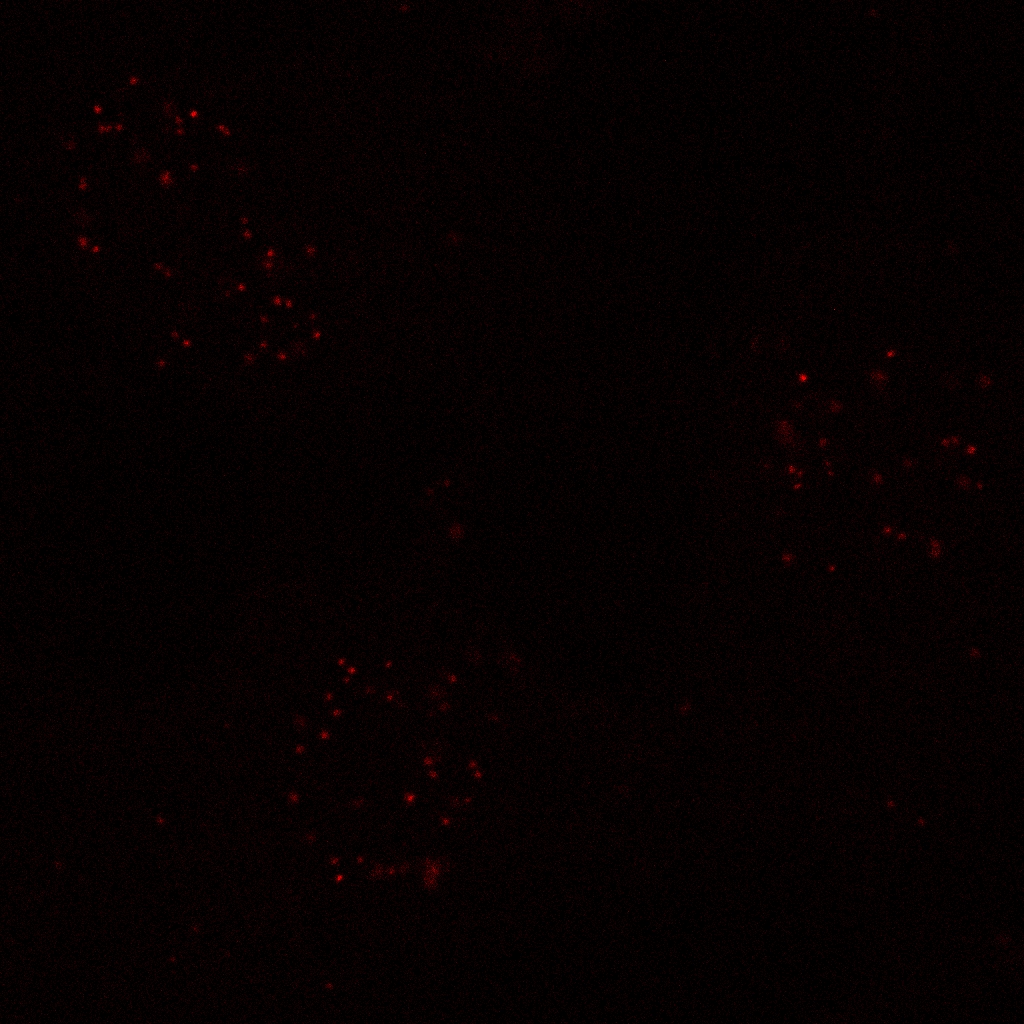

Supplement: Supplementary file 5 — Source data Fig. 4 [file 44318_2024_85_MOESM5_ESM.zip › SD Figure 4/4E high resolution/siIPMK PLA.jpg]

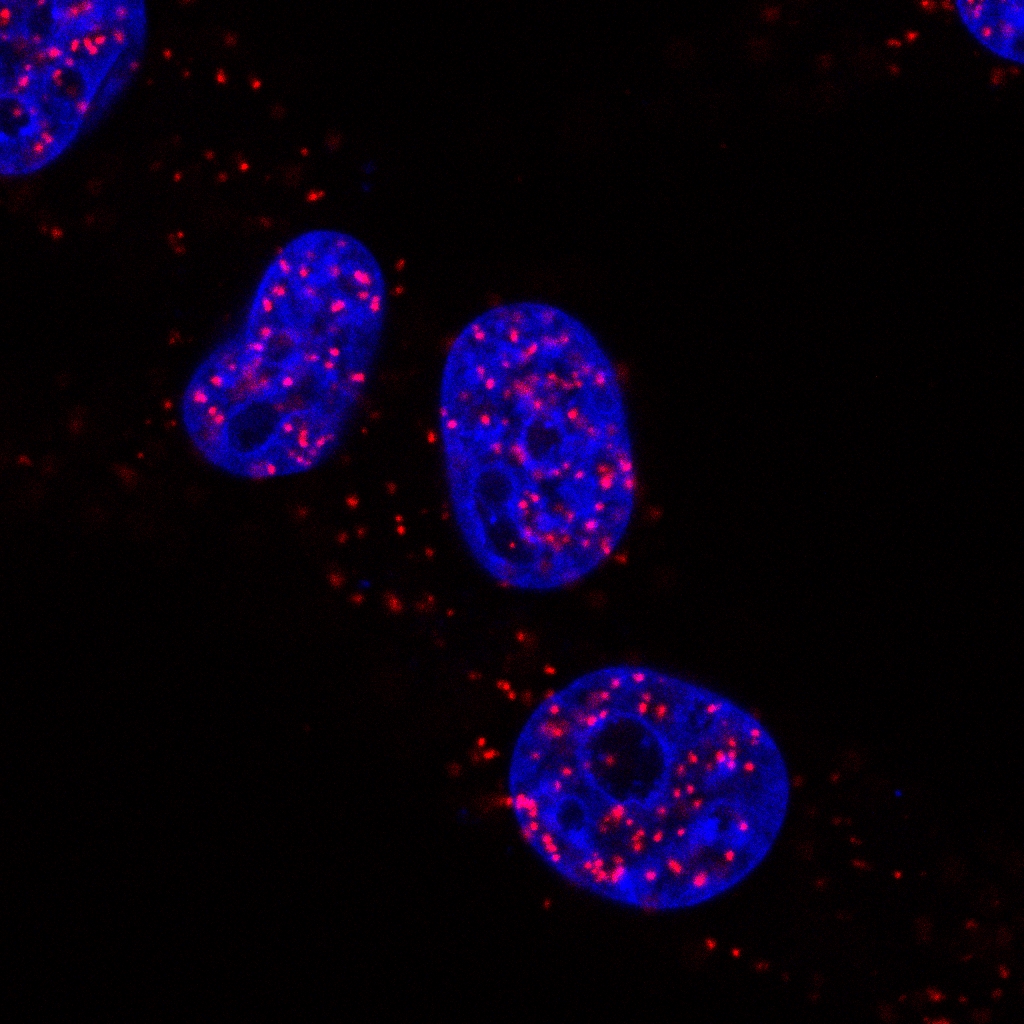

Supplement: Supplementary file 5 — Source data Fig. 4 [file 44318_2024_85_MOESM5_ESM.zip › SD Figure 4/4E high resolution/sicon merge.jpg]

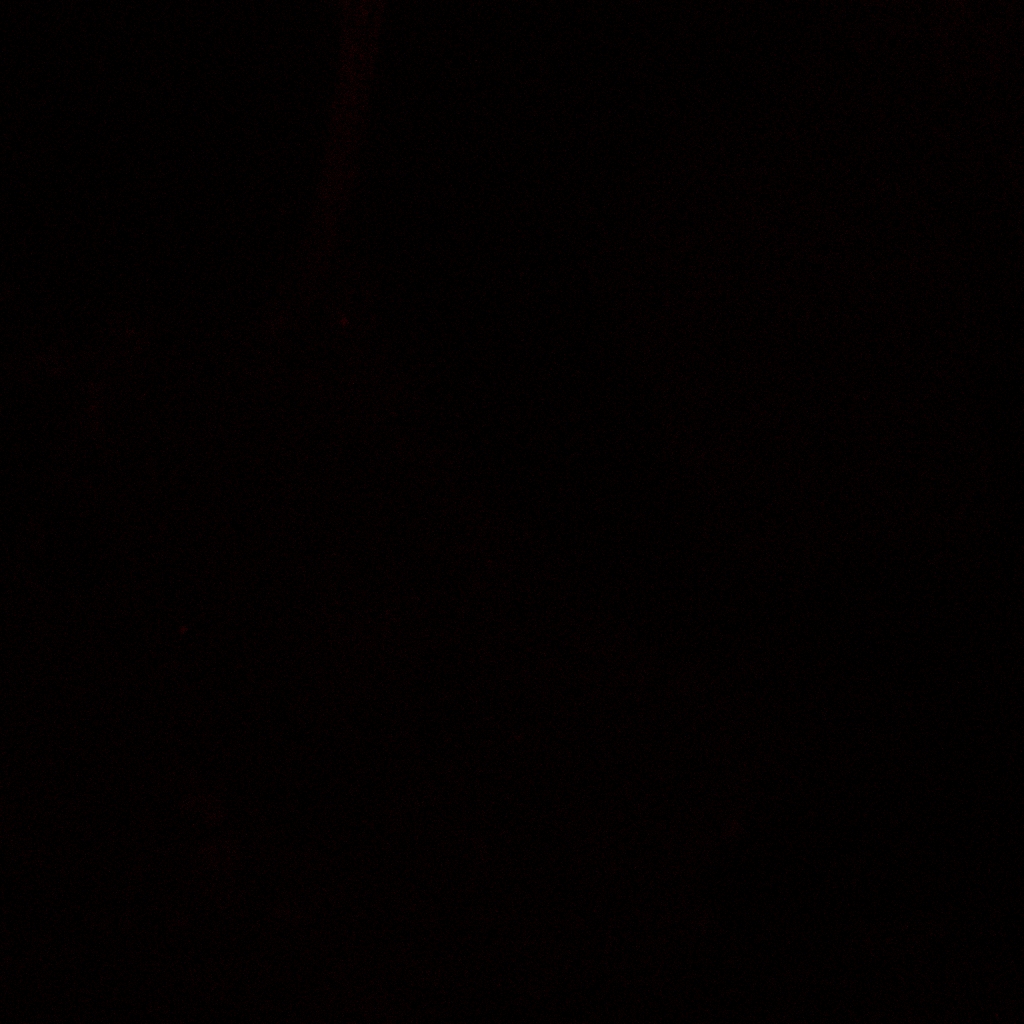

Supplement: Supplementary file 5 — Source data Fig. 4 [file 44318_2024_85_MOESM5_ESM.zip › SD Figure 4/4E high resolution/siIA PLA.jpg]

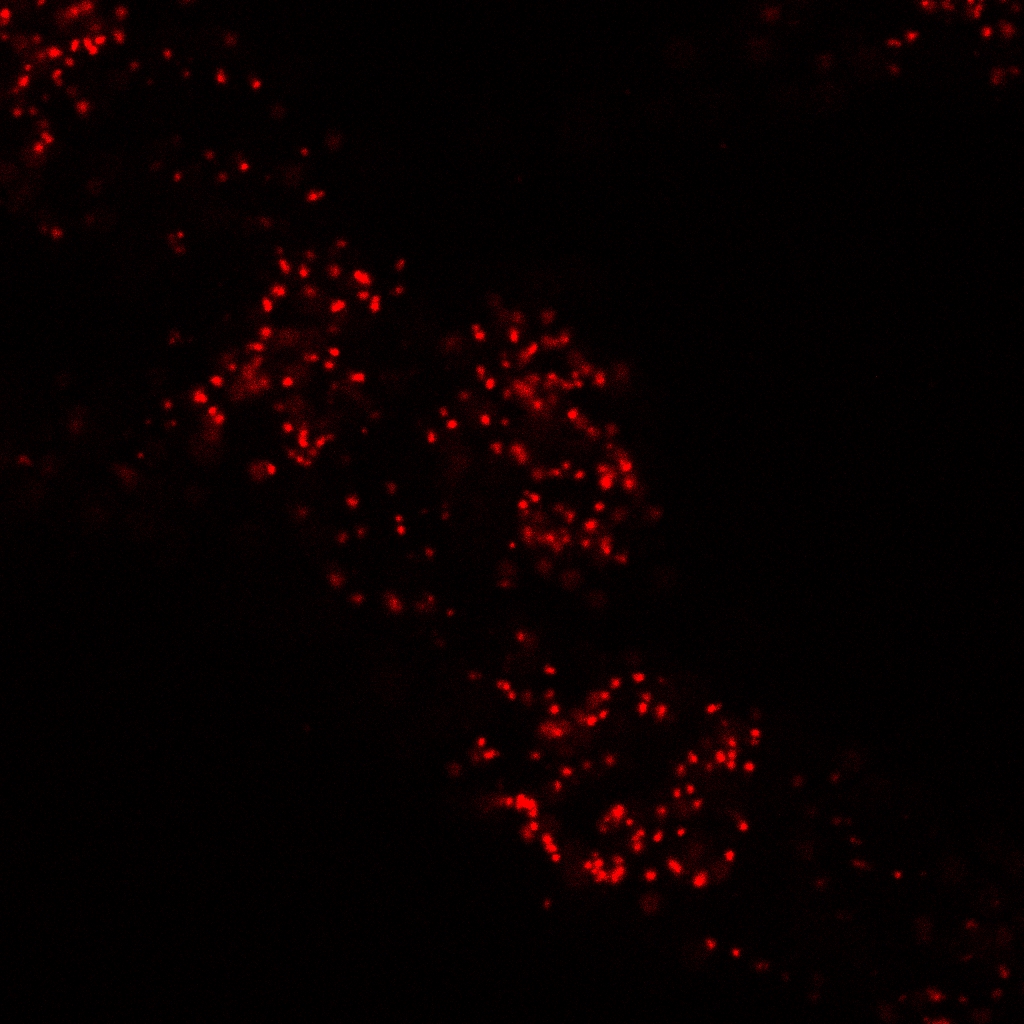

Supplement: Supplementary file 5 — Source data Fig. 4 [file 44318_2024_85_MOESM5_ESM.zip › SD Figure 4/4E high resolution/sicon PLA.jpg]

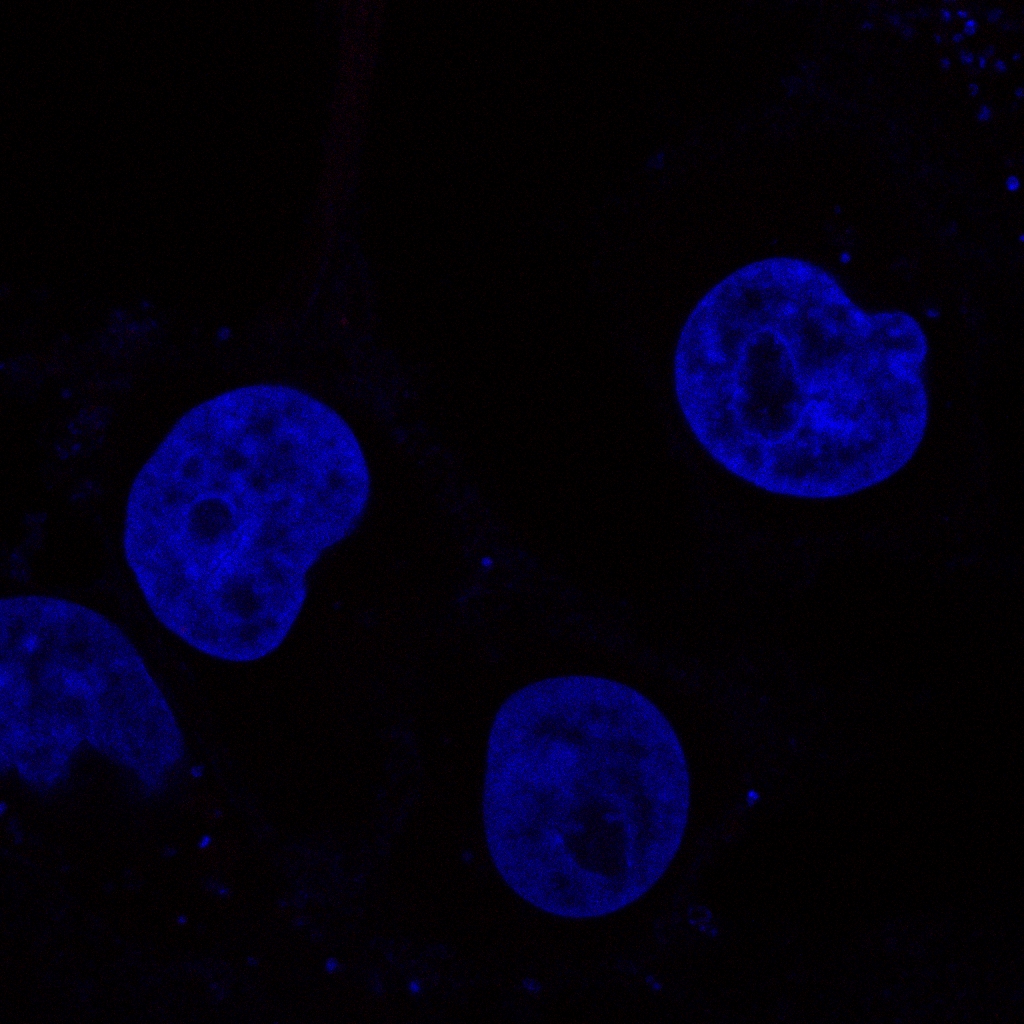

Supplement: Supplementary file 5 — Source data Fig. 4 [file 44318_2024_85_MOESM5_ESM.zip › SD Figure 4/4E high resolution/siIA DAPI.jpg]

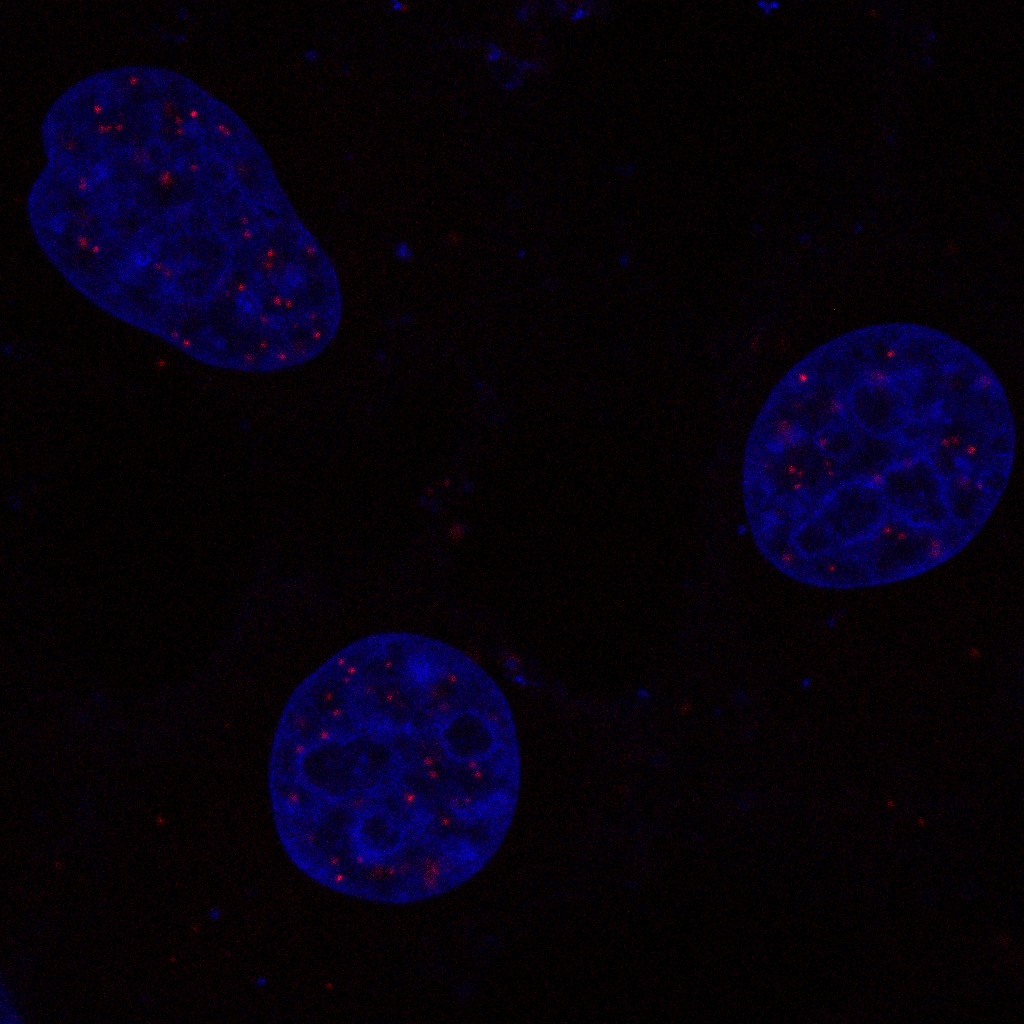

Supplement: Supplementary file 5 — Source data Fig. 4 [file 44318_2024_85_MOESM5_ESM.zip › SD Figure 4/4E high resolution/siIPMK merge.jpg]

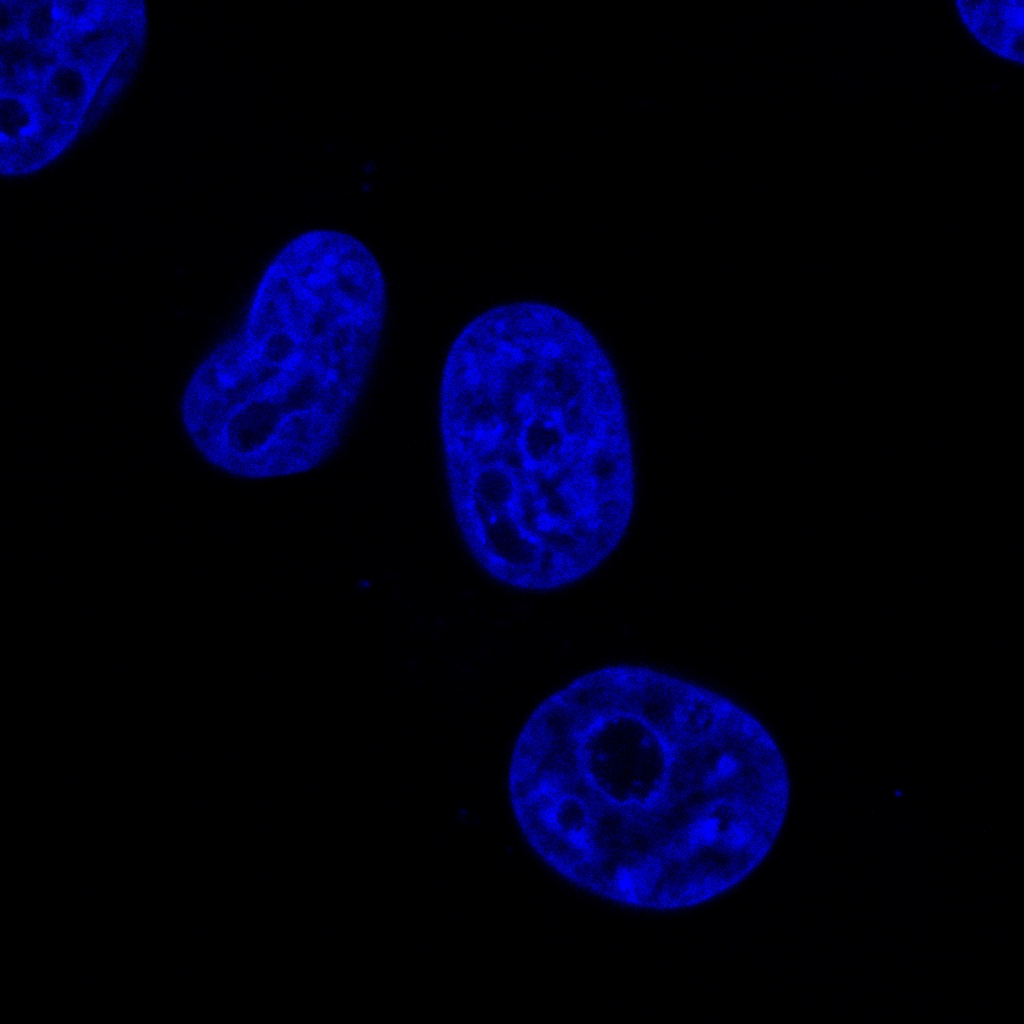

Supplement: Supplementary file 5 — Source data Fig. 4 [file 44318_2024_85_MOESM5_ESM.zip › SD Figure 4/4E high resolution/sicon DAPI.jpg]

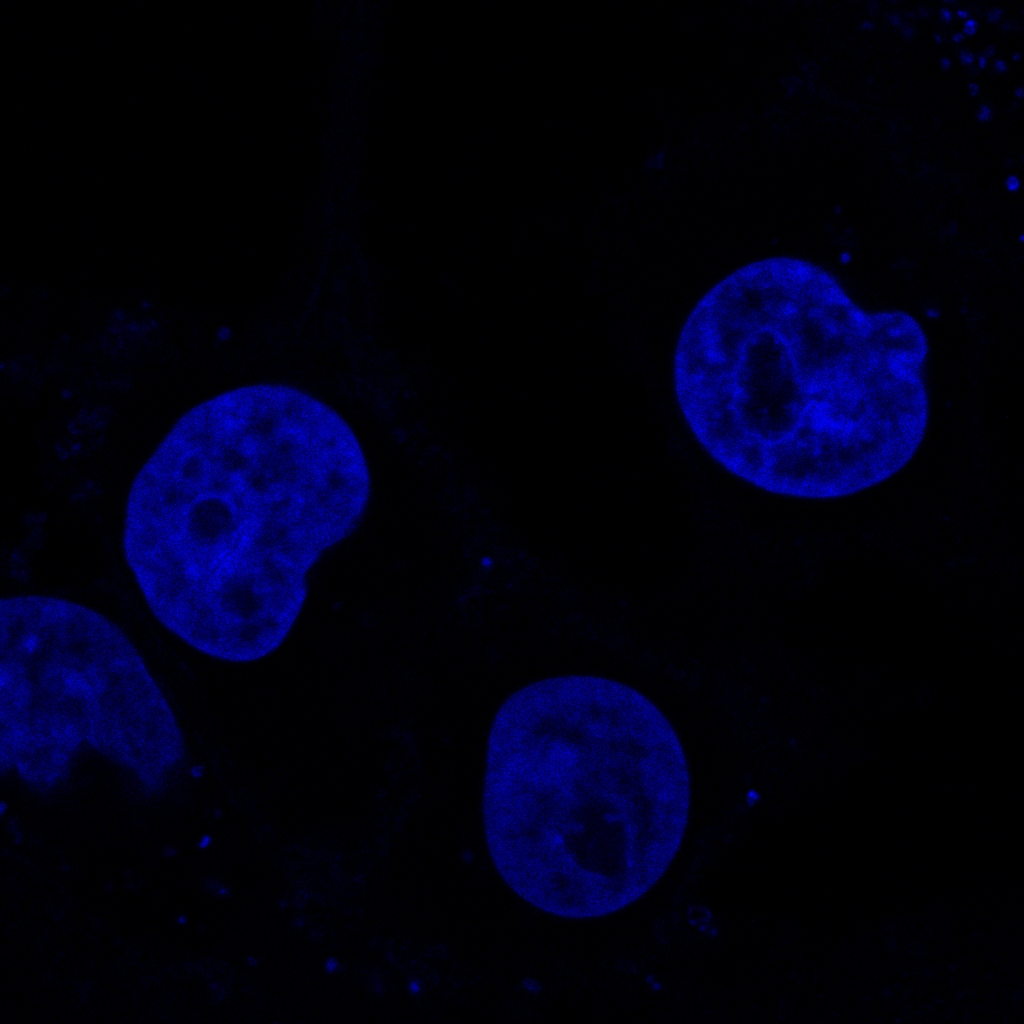

Supplement: Supplementary file 5 — Source data Fig. 4 [file 44318_2024_85_MOESM5_ESM.zip › SD Figure 4/4E high resolution/siIA merge.jpg]

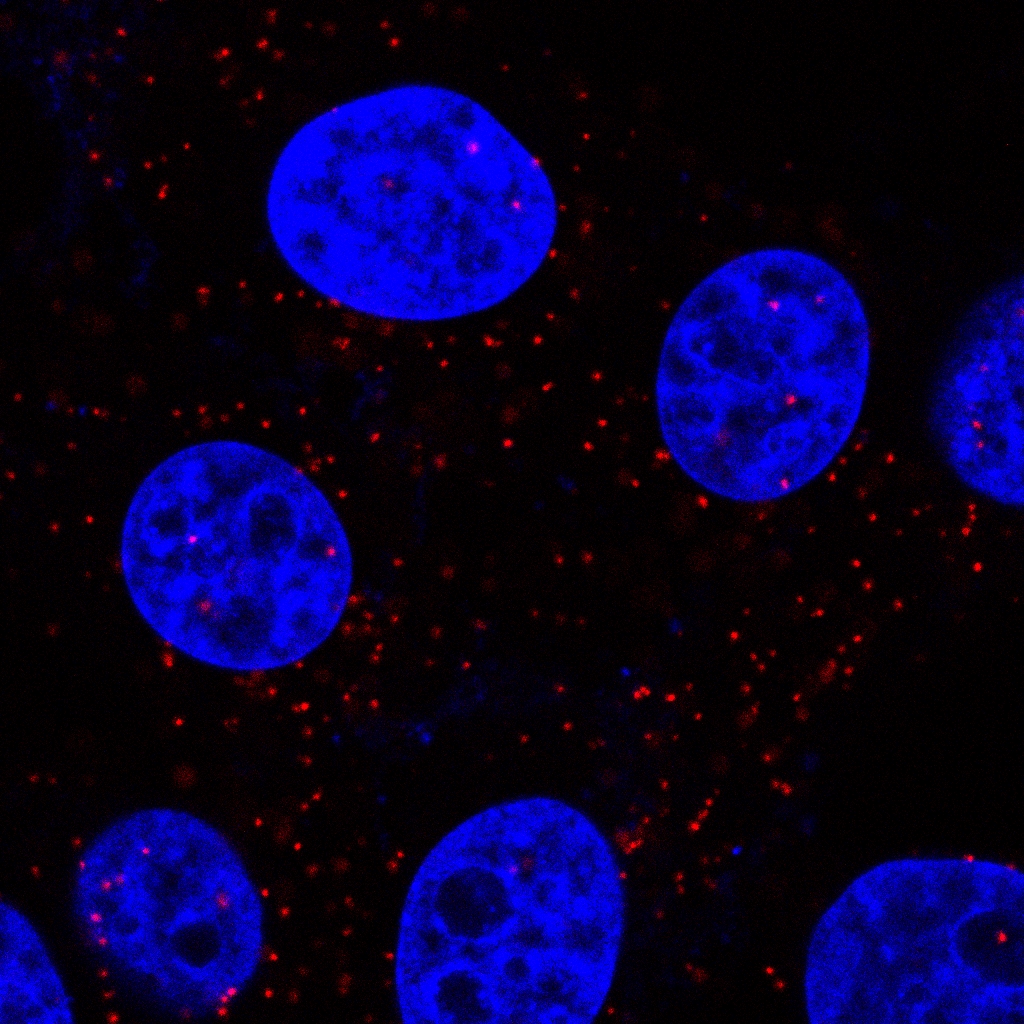

Supplement: Supplementary file 5 — Source data Fig. 4 [file 44318_2024_85_MOESM5_ESM.zip › SD Figure 4/4C high resolution/Free merge.jpg]

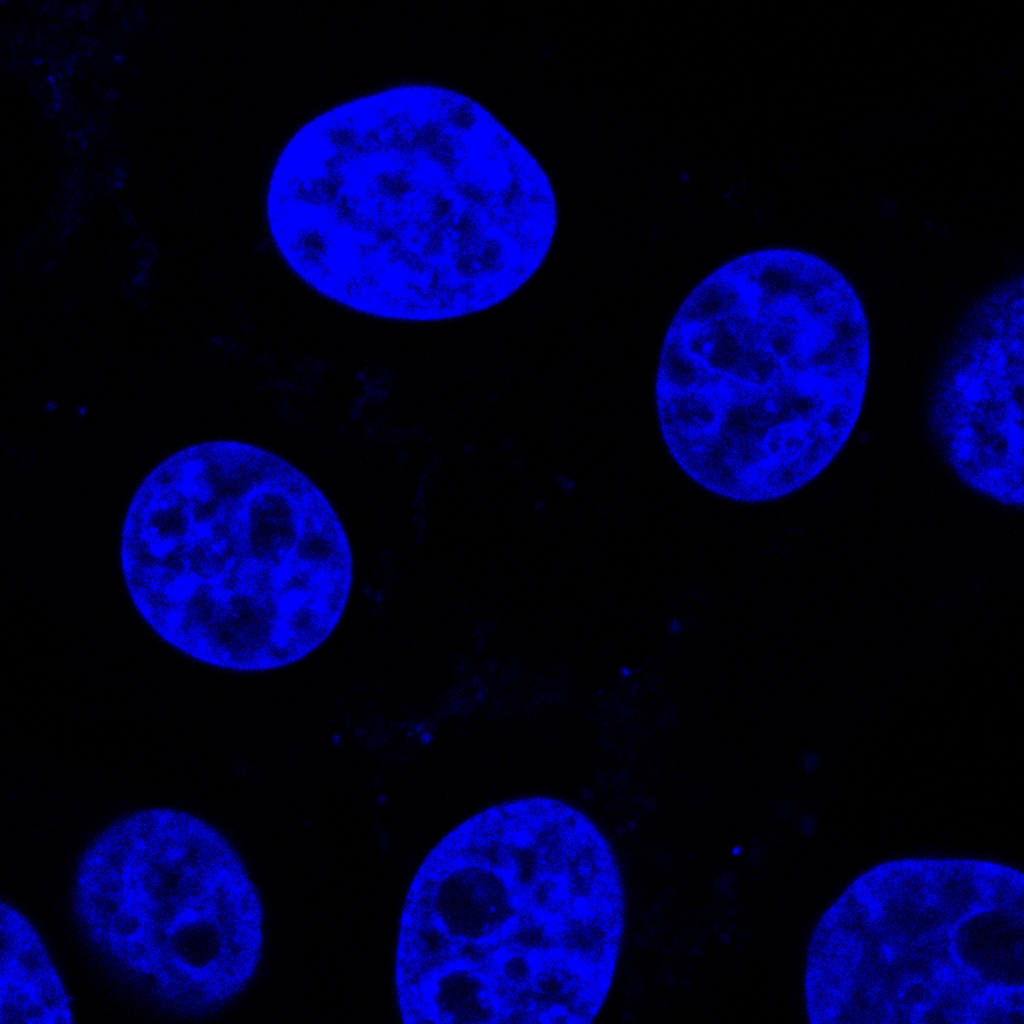

Supplement: Supplementary file 5 — Source data Fig. 4 [file 44318_2024_85_MOESM5_ESM.zip › SD Figure 4/4C high resolution/Free DAPI.jpg]

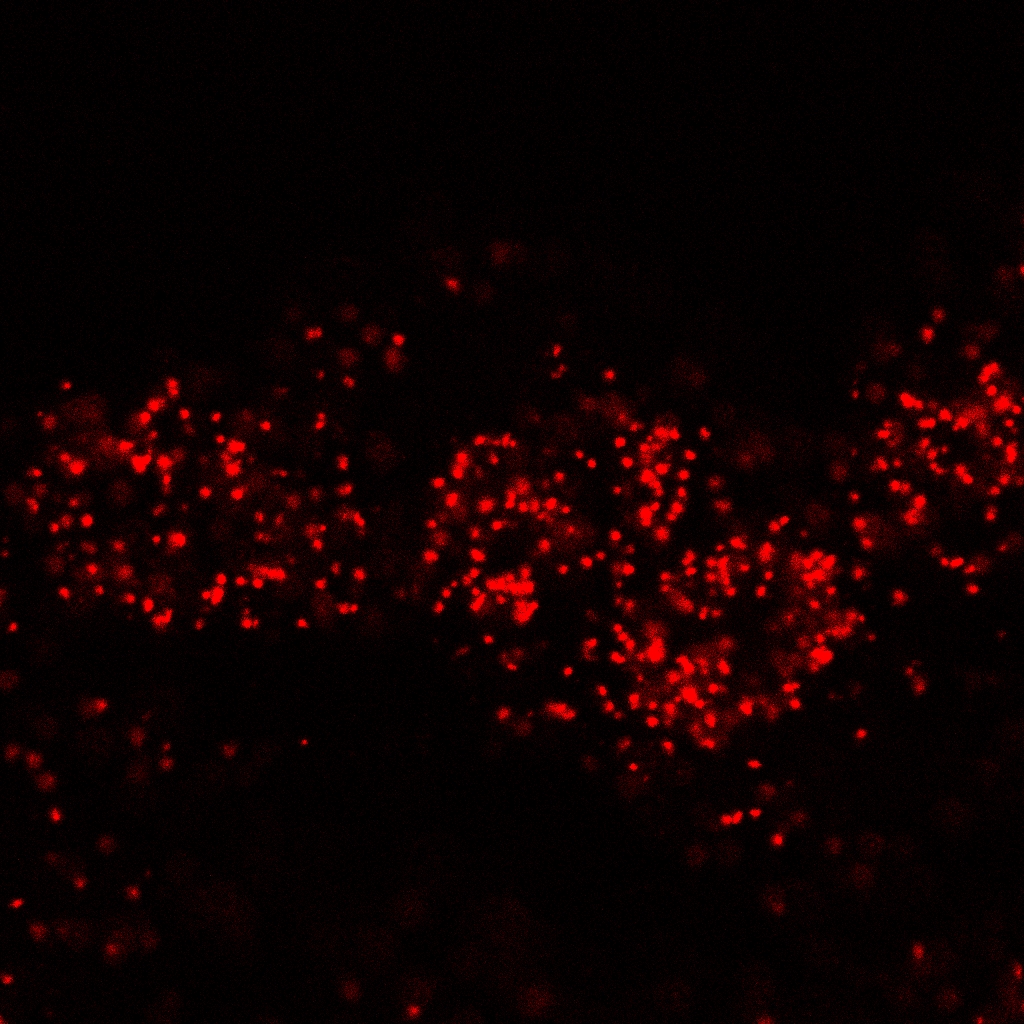

Supplement: Supplementary file 5 — Source data Fig. 4 [file 44318_2024_85_MOESM5_ESM.zip › SD Figure 4/4C high resolution/Serum PLA.jpg]

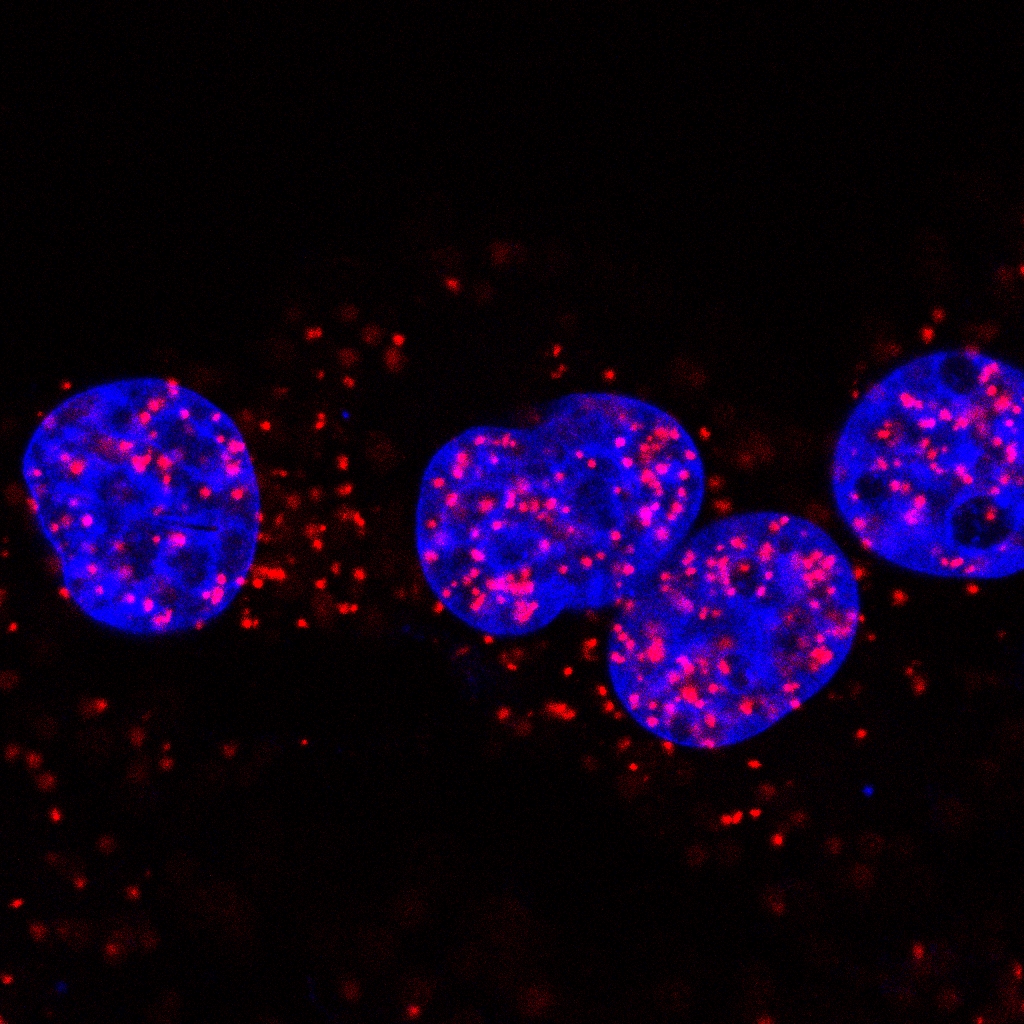

Supplement: Supplementary file 5 — Source data Fig. 4 [file 44318_2024_85_MOESM5_ESM.zip › SD Figure 4/4C high resolution/Serum merge.jpg]

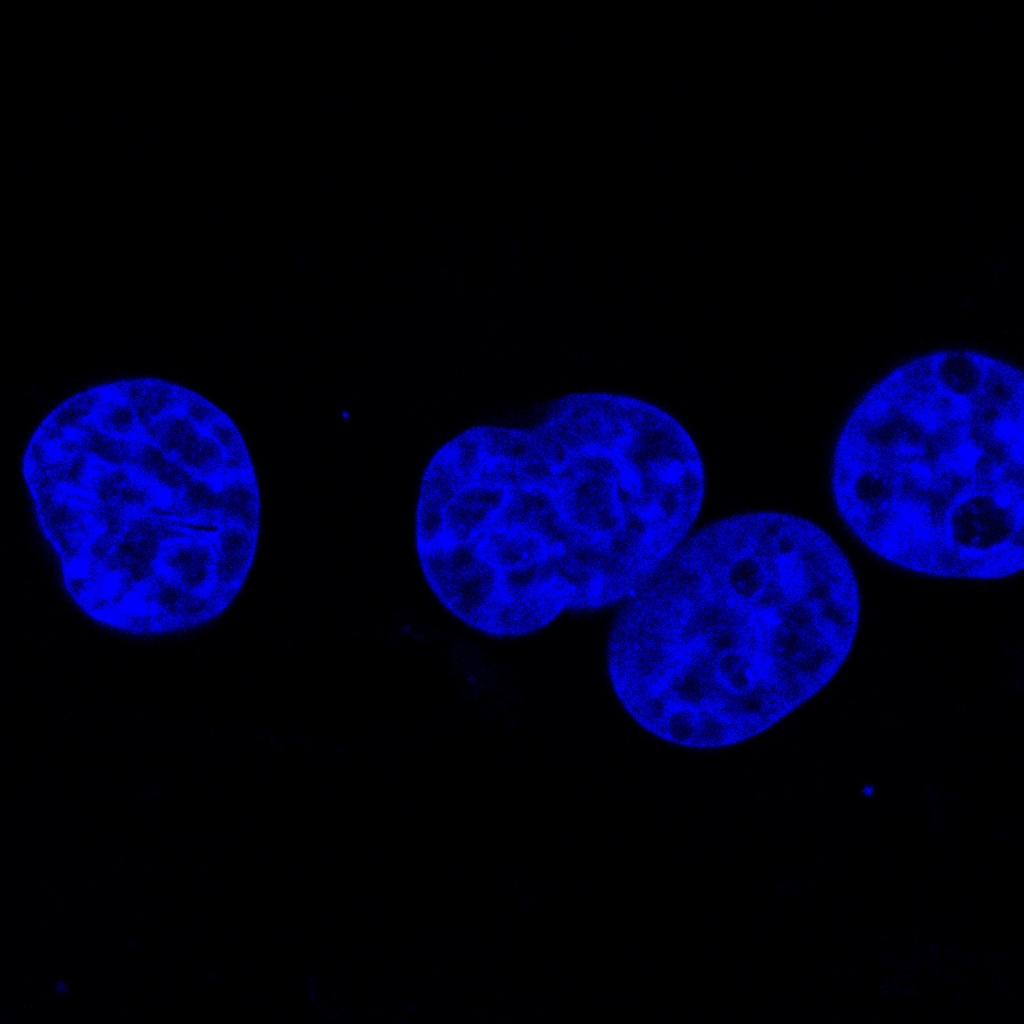

Supplement: Supplementary file 5 — Source data Fig. 4 [file 44318_2024_85_MOESM5_ESM.zip › SD Figure 4/4C high resolution/Serum DAPI.jpg]

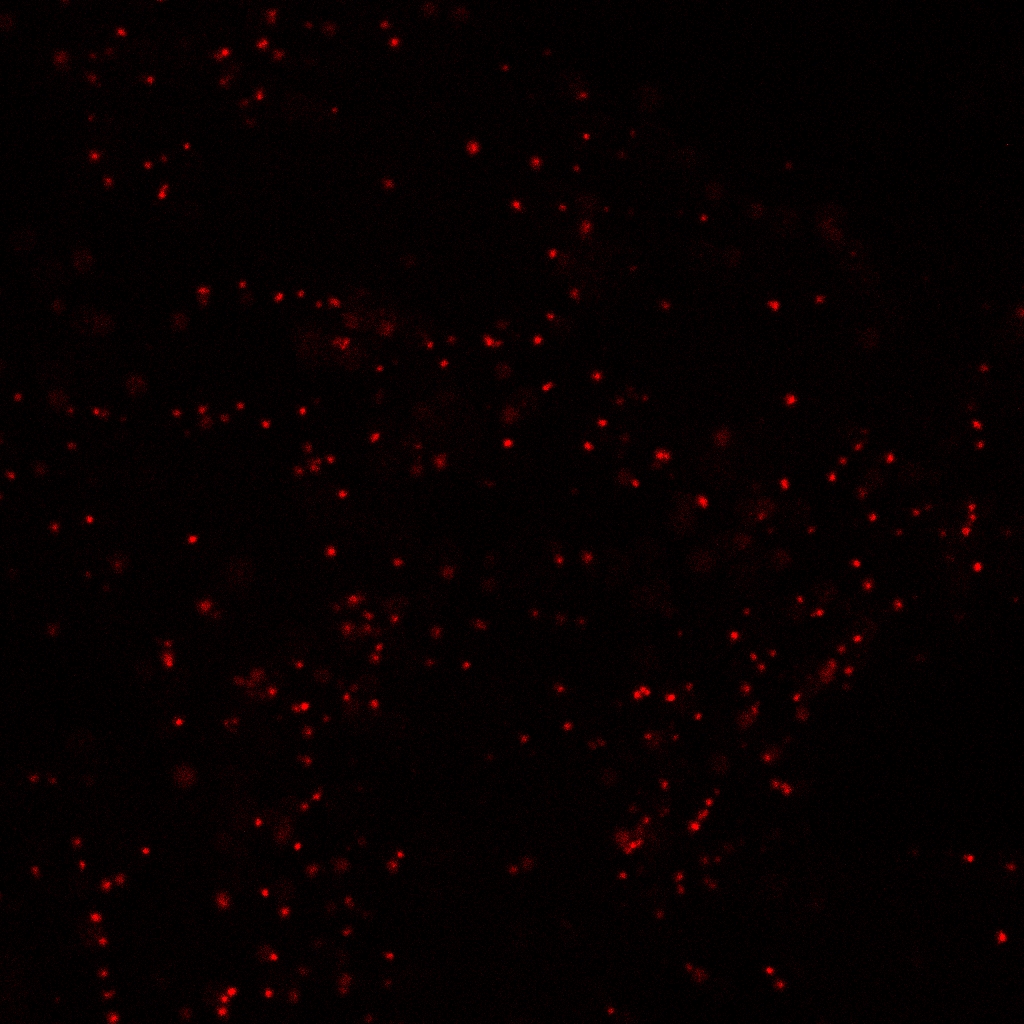

Supplement: Supplementary file 5 — Source data Fig. 4 [file 44318_2024_85_MOESM5_ESM.zip › SD Figure 4/4C high resolution/Free PLA.jpg]

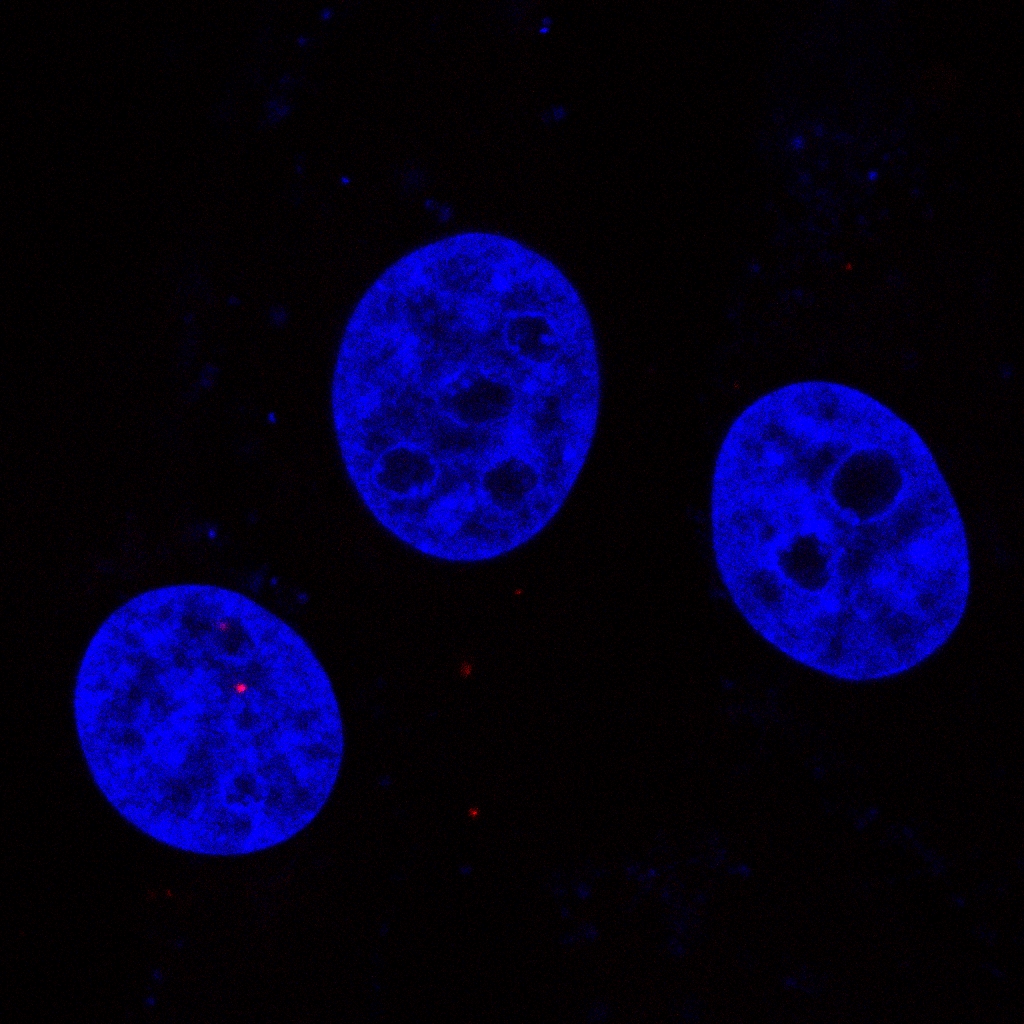

Supplement: Supplementary file 5 — Source data Fig. 4 [file 44318_2024_85_MOESM5_ESM.zip › SD Figure 4/4D high resolution/Free merge.jpg]

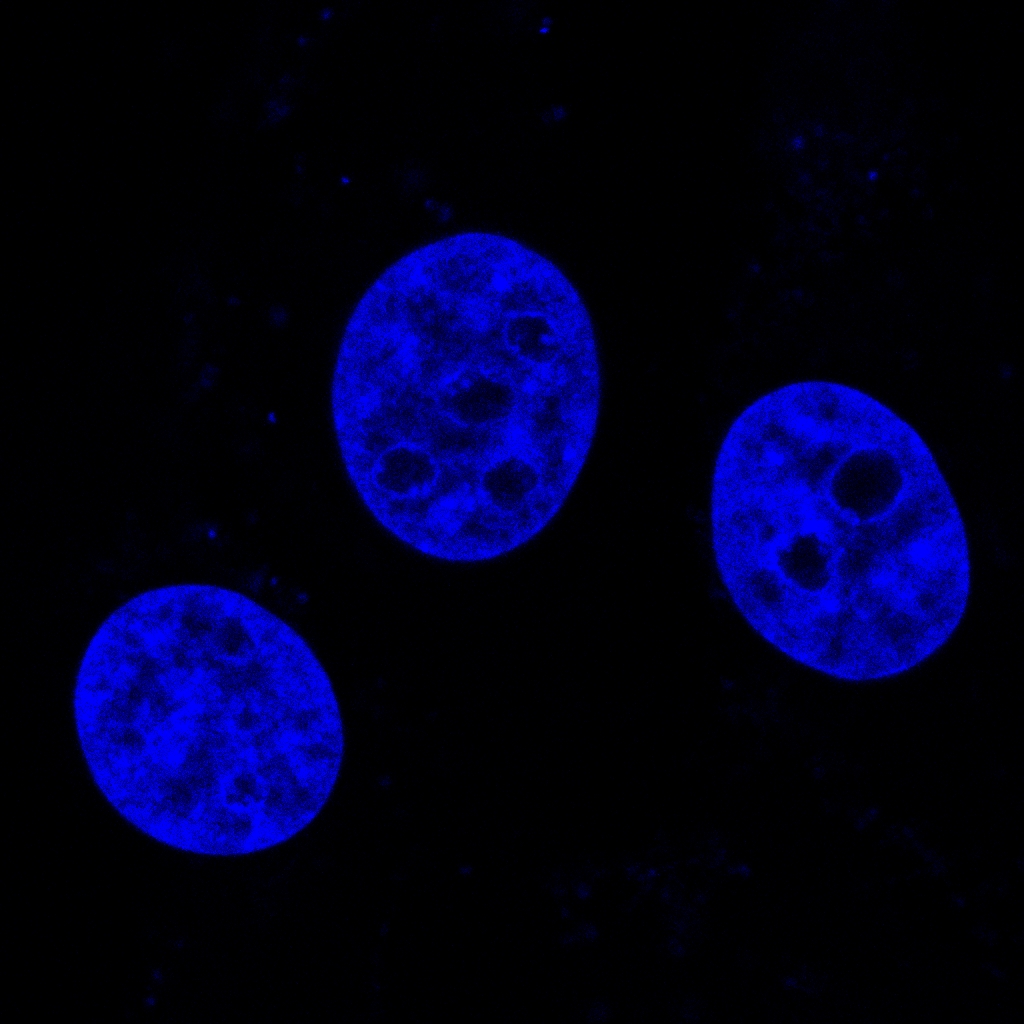

Supplement: Supplementary file 5 — Source data Fig. 4 [file 44318_2024_85_MOESM5_ESM.zip › SD Figure 4/4D high resolution/Free DAPI.jpg]

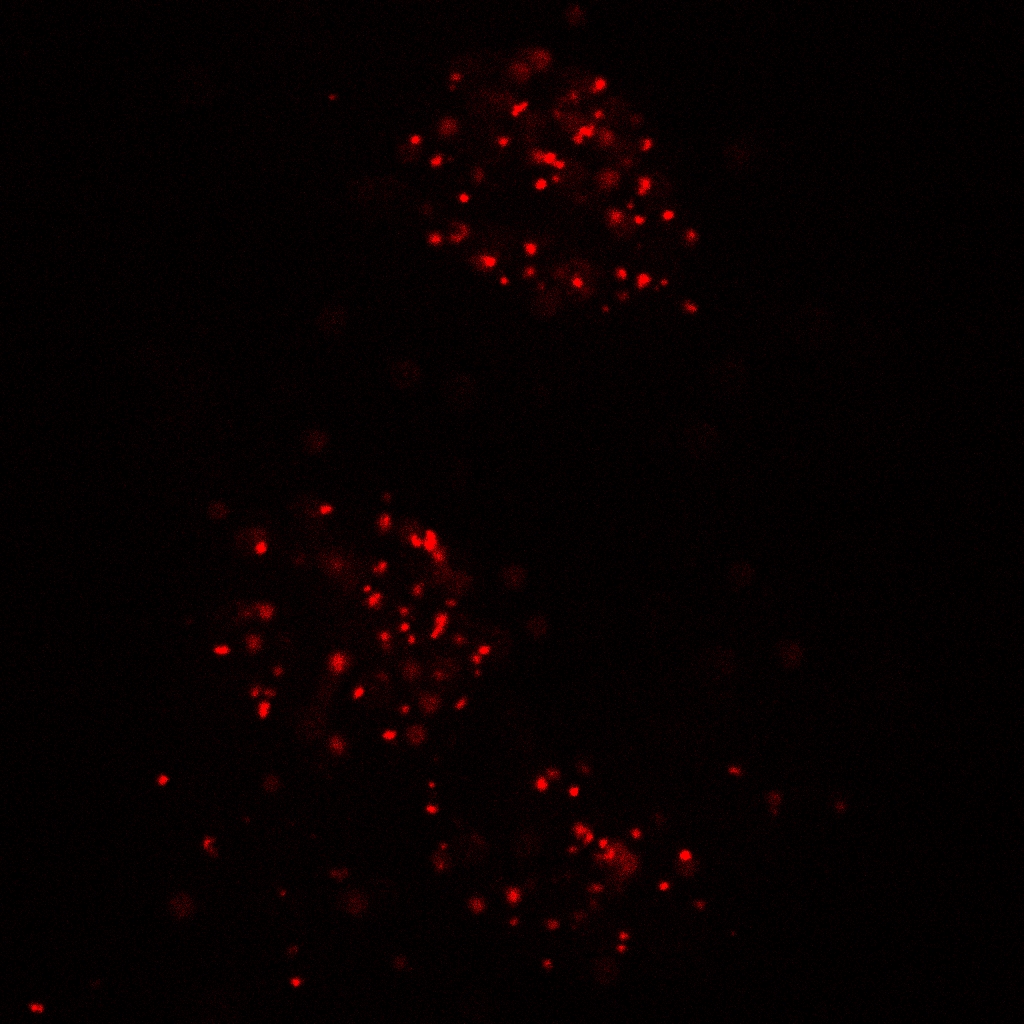

Supplement: Supplementary file 5 — Source data Fig. 4 [file 44318_2024_85_MOESM5_ESM.zip › SD Figure 4/4D high resolution/Serum PLA.jpg]

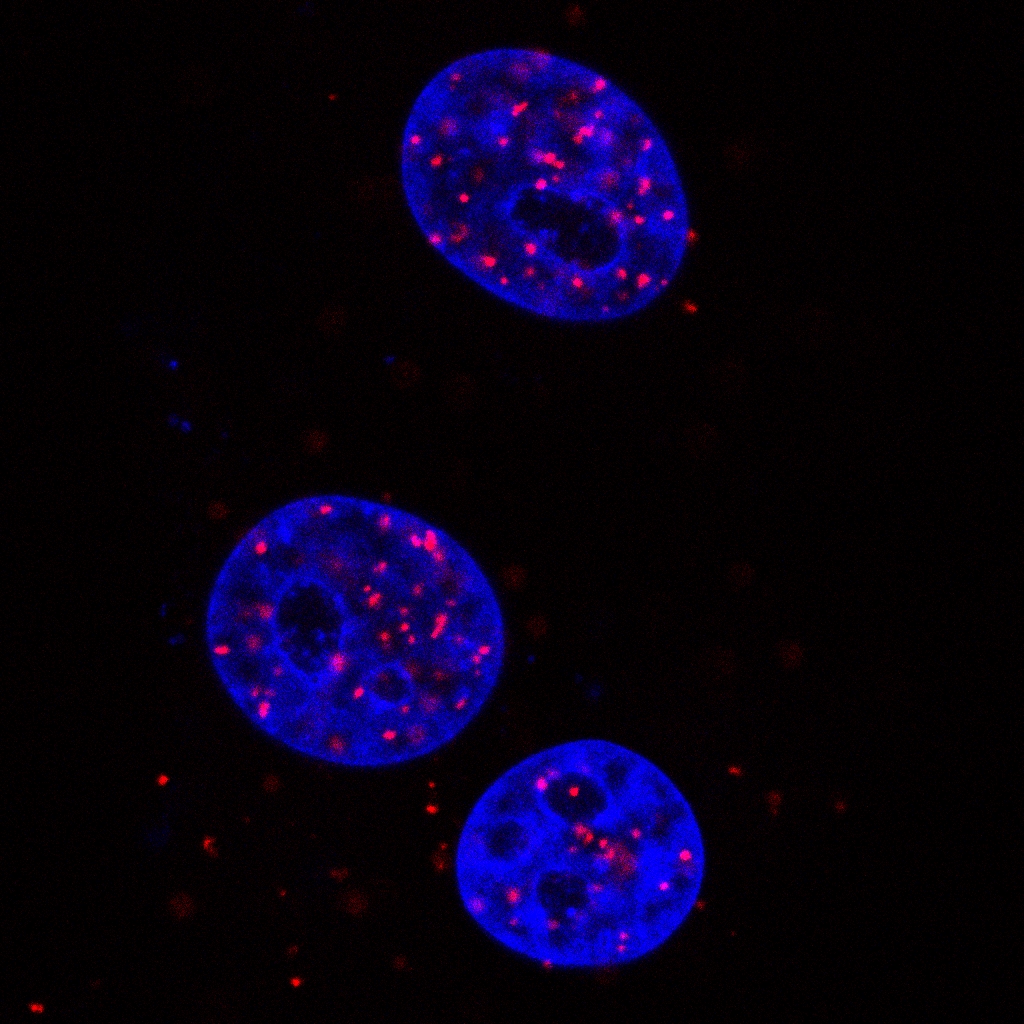

Supplement: Supplementary file 5 — Source data Fig. 4 [file 44318_2024_85_MOESM5_ESM.zip › SD Figure 4/4D high resolution/Serum merge.jpg]

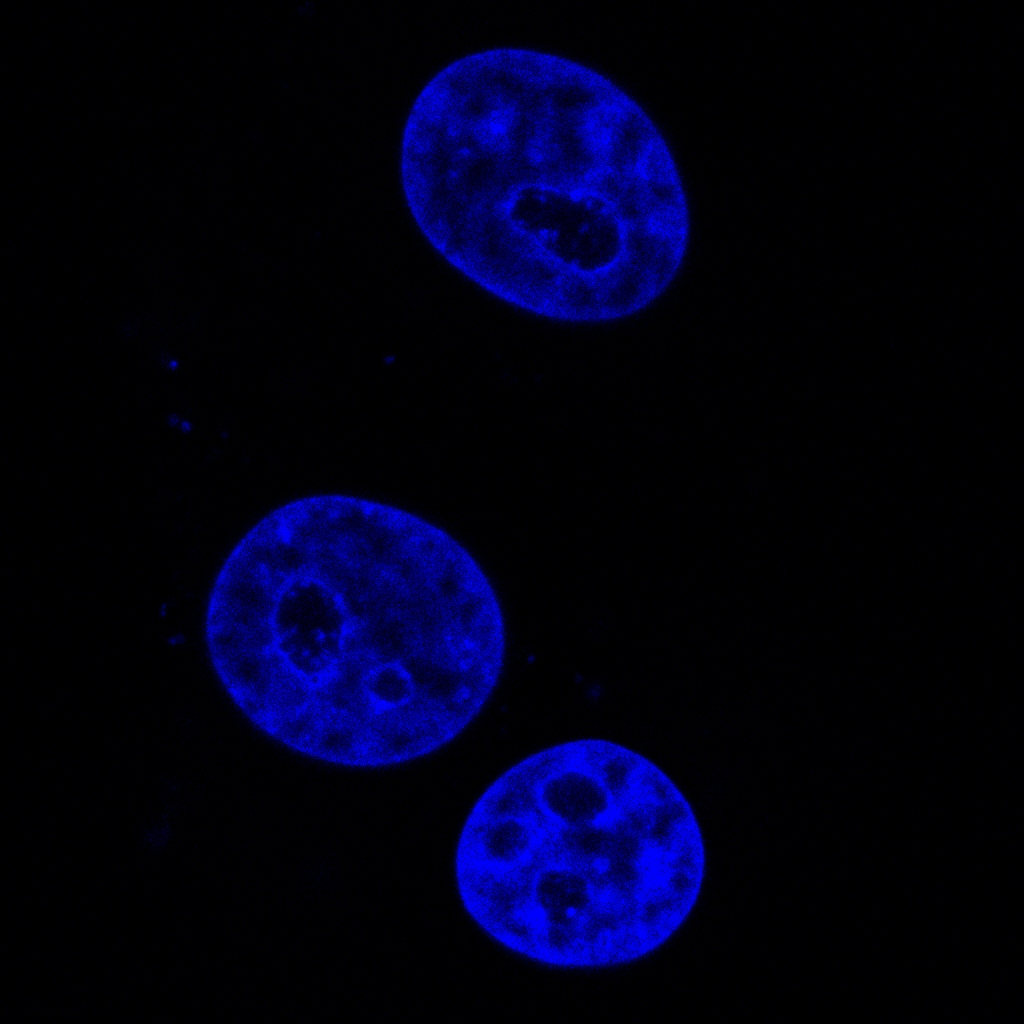

Supplement: Supplementary file 5 — Source data Fig. 4 [file 44318_2024_85_MOESM5_ESM.zip › SD Figure 4/4D high resolution/Serum DAPI.jpg]

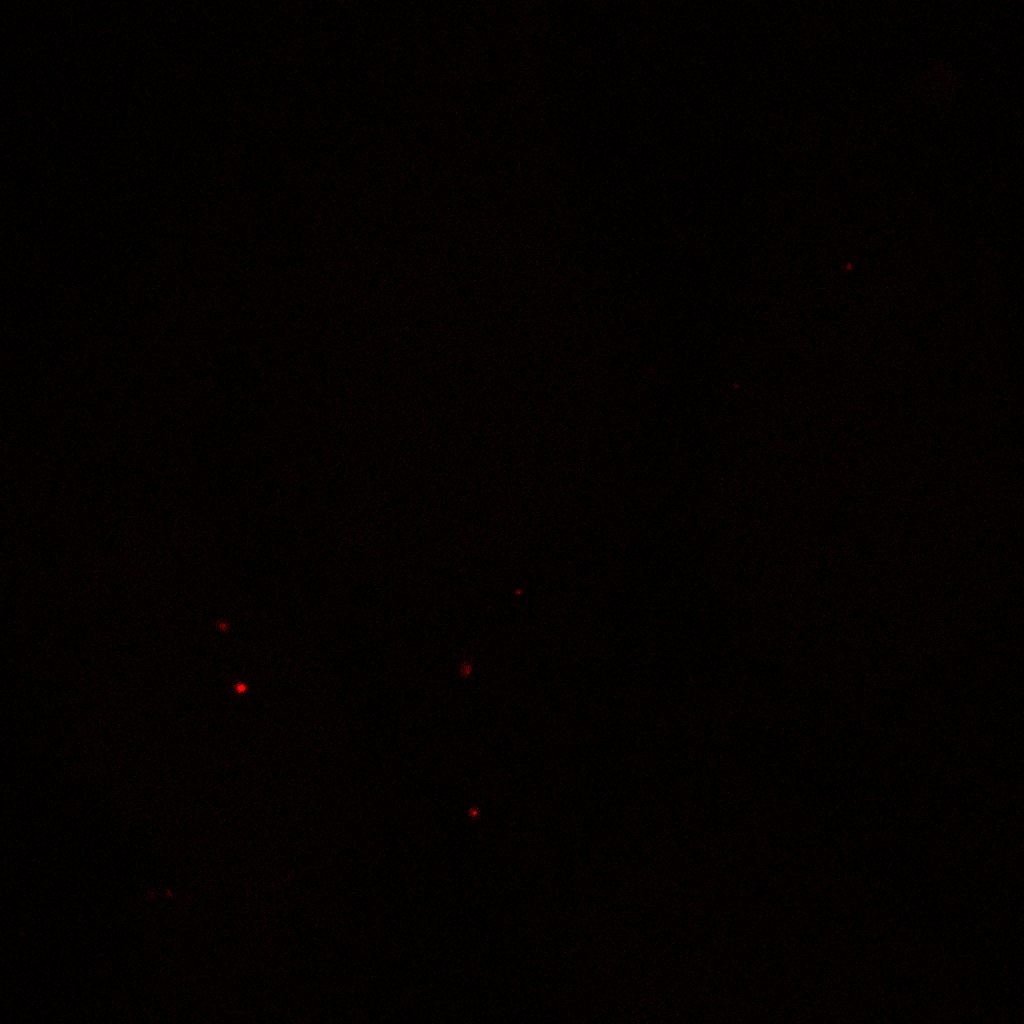

Supplement: Supplementary file 5 — Source data Fig. 4 [file 44318_2024_85_MOESM5_ESM.zip › SD Figure 4/4D high resolution/Free PLA.jpg]

## Slide 1
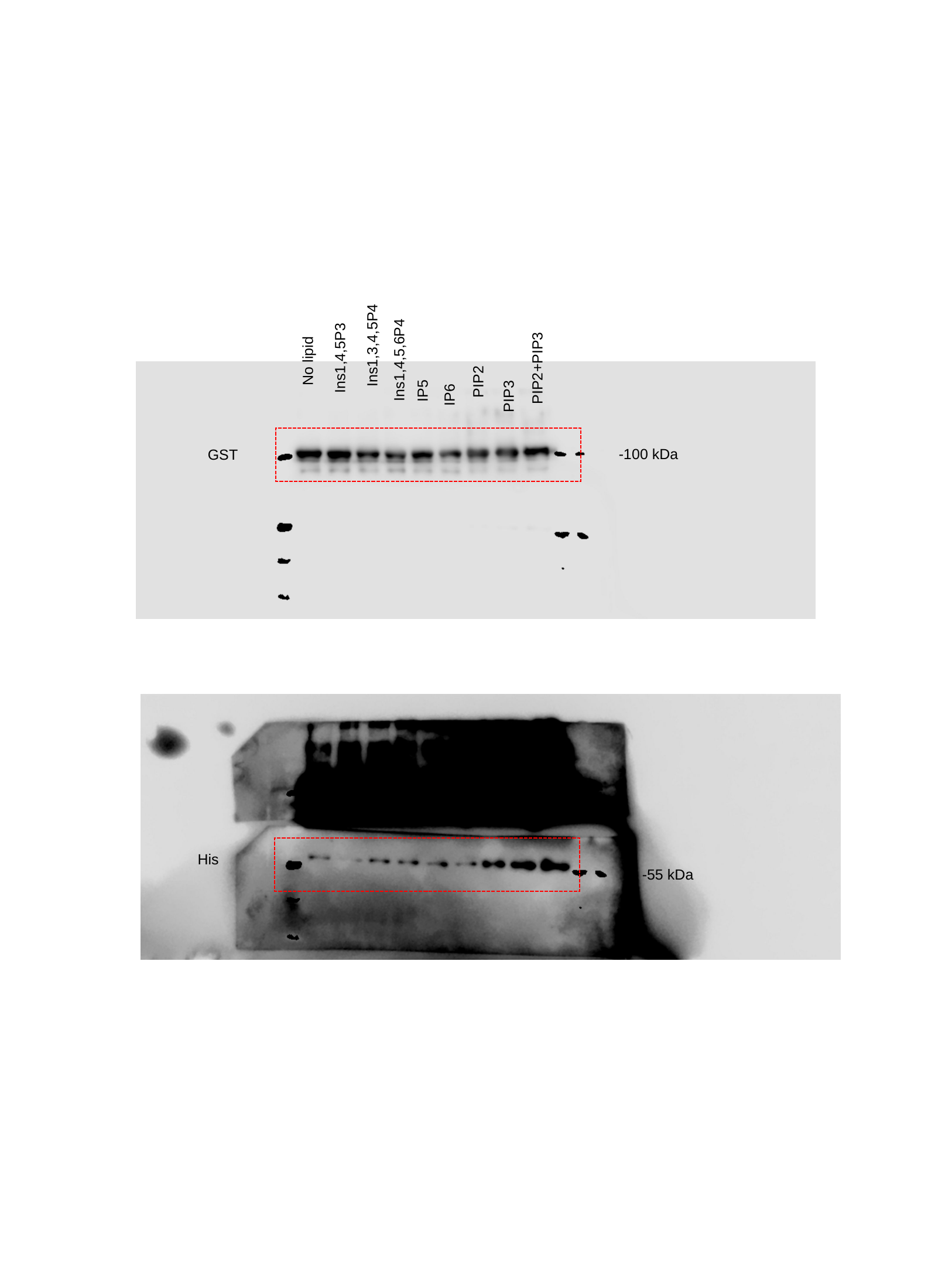

No lipid
Ins1,3,4,5P4
Ins1,4,5P3
PIP2
Ins1,4,5,6P4
IP5
PIP2+PIP3
IP6
PIP3
-100 kDa
GST
His
-55 kDa

Supplement: Supplementary file 6 — Source data Fig. 5 [file 44318_2024_85_MOESM6_ESM.zip › SD Figure 5/5F.pptx]

## Slide 1
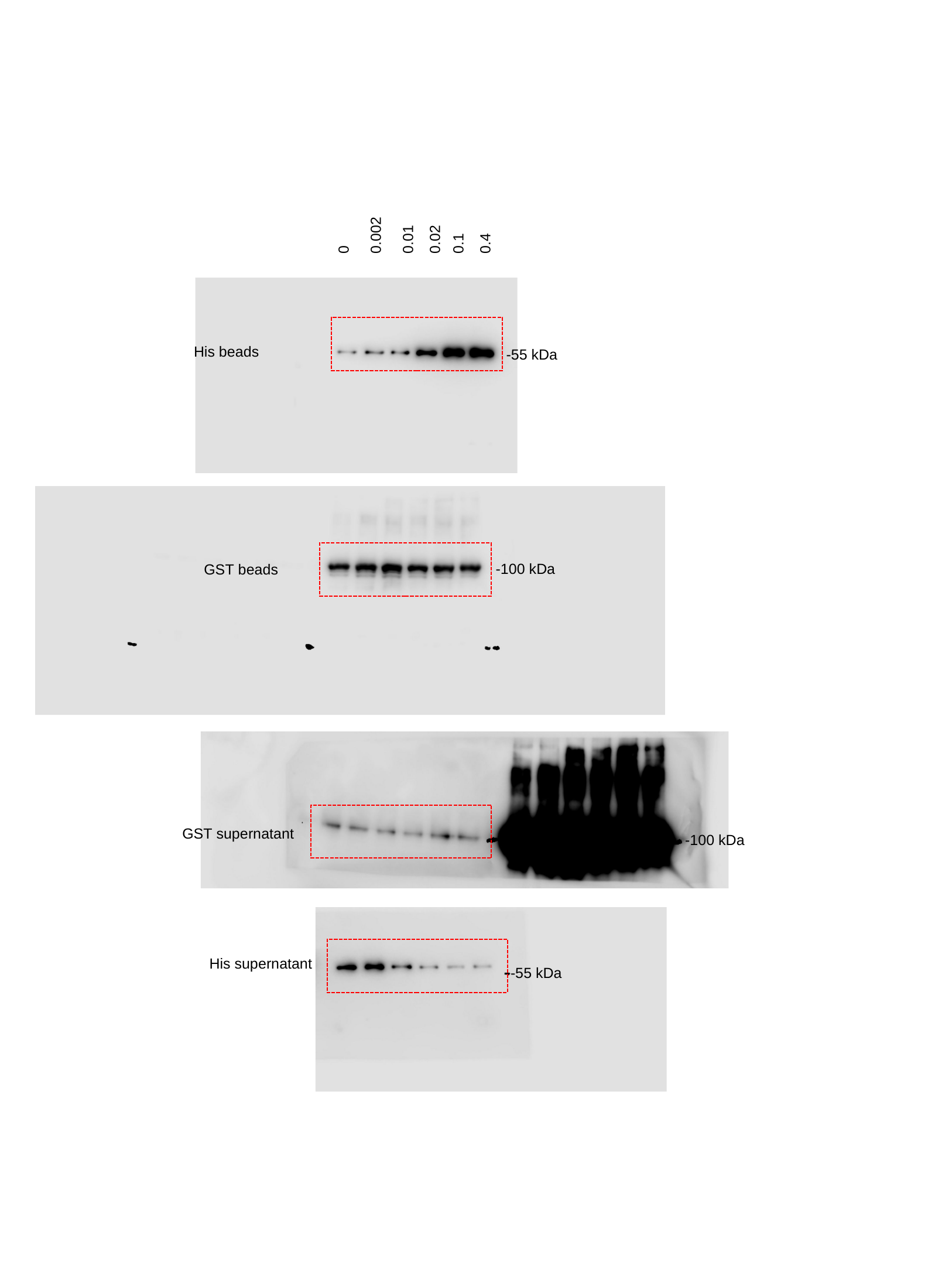

0
0.002
0.01
0.02
0.1
0.4
His beads
-55 kDa
-100 kDa
GST beads
GST supernatant
-100 kDa
His supernatant
-55 kDa

Supplement: Supplementary file 6 — Source data Fig. 5 [file 44318_2024_85_MOESM6_ESM.zip › SD Figure 5/5E.pptx]

## Slide 1
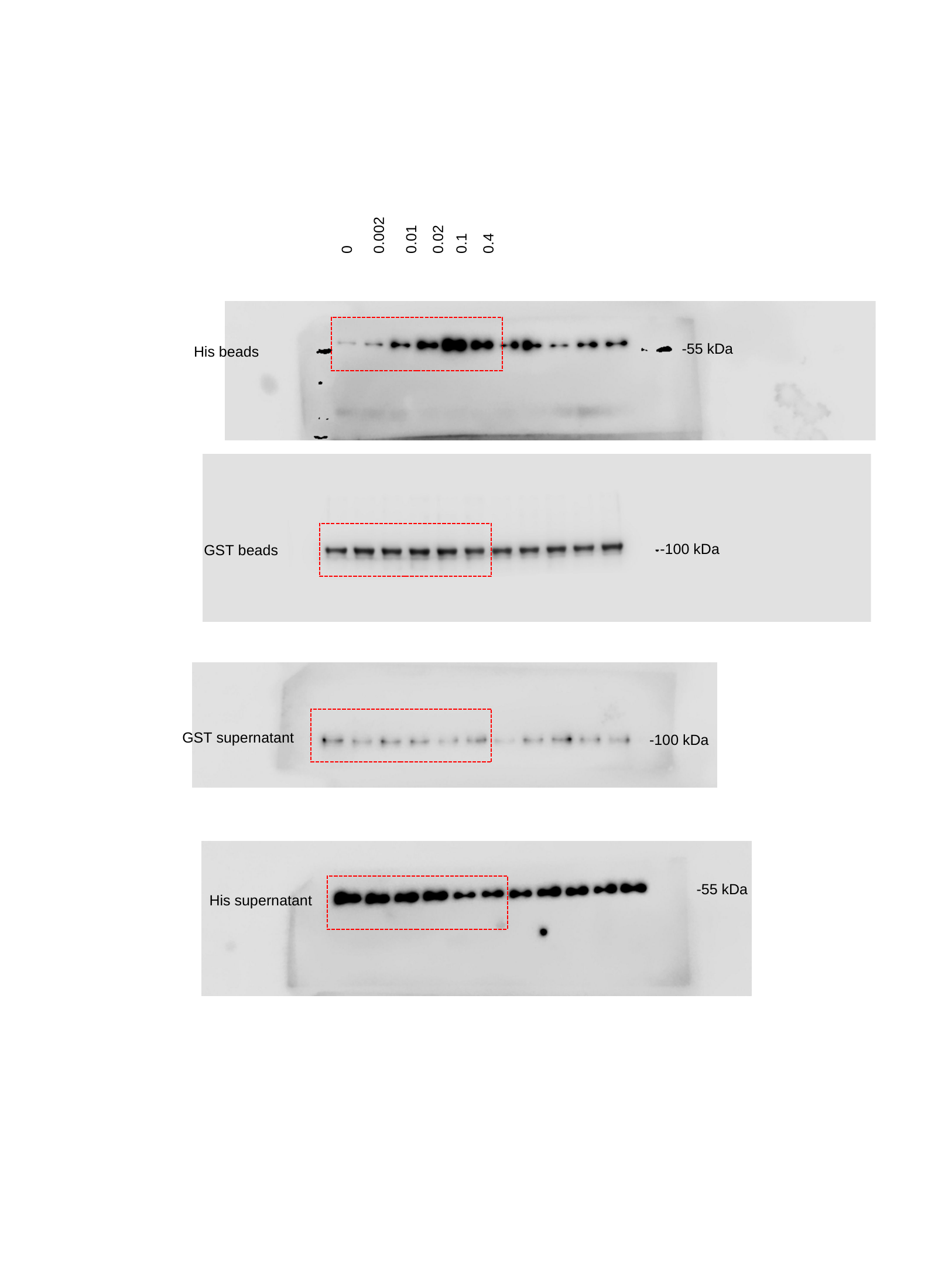

0
0.002
0.01
0.02
0.1
0.4
-55 kDa
His beads
-100 kDa
GST beads
GST supernatant
-100 kDa
-55 kDa
His supernatant

Supplement: Supplementary file 6 — Source data Fig. 5 [file 44318_2024_85_MOESM6_ESM.zip › SD Figure 5/5D.pptx]

## Slide 1
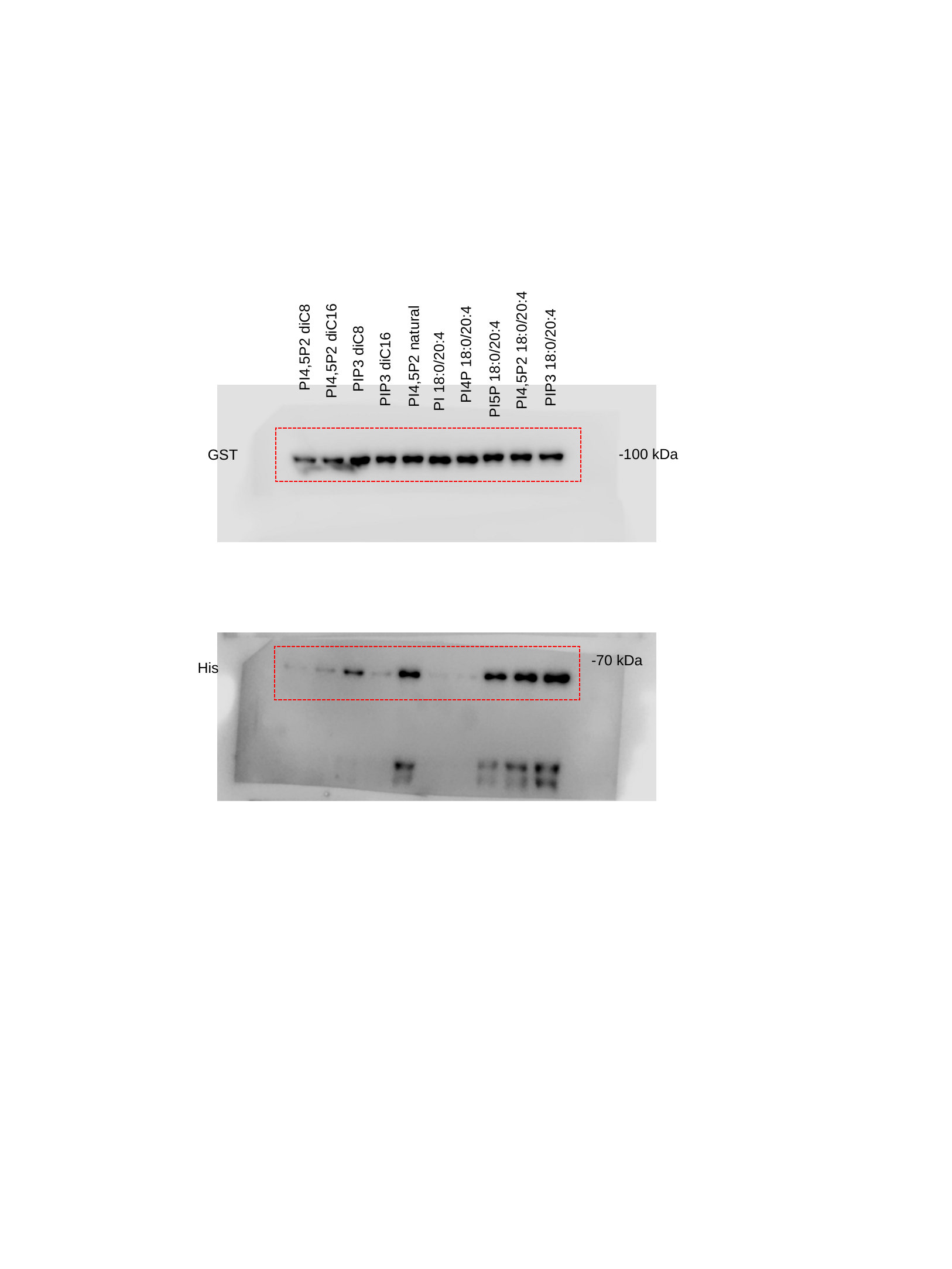

PI4,5P2 diC8
PIP3 diC8
PI4,5P2 diC16
PI4P 18:0/20:4
PIP3 diC16
PIP3 18:0/20:4
PI4,5P2 natural
PI4,5P2 18:0/20:4
PI 18:0/20:4
PI5P 18:0/20:4
-100 kDa
GST
-70 kDa
His

Supplement: Supplementary file 6 — Source data Fig. 5 [file 44318_2024_85_MOESM6_ESM.zip › SD Figure 5/5C.pptx]

## Slide 1
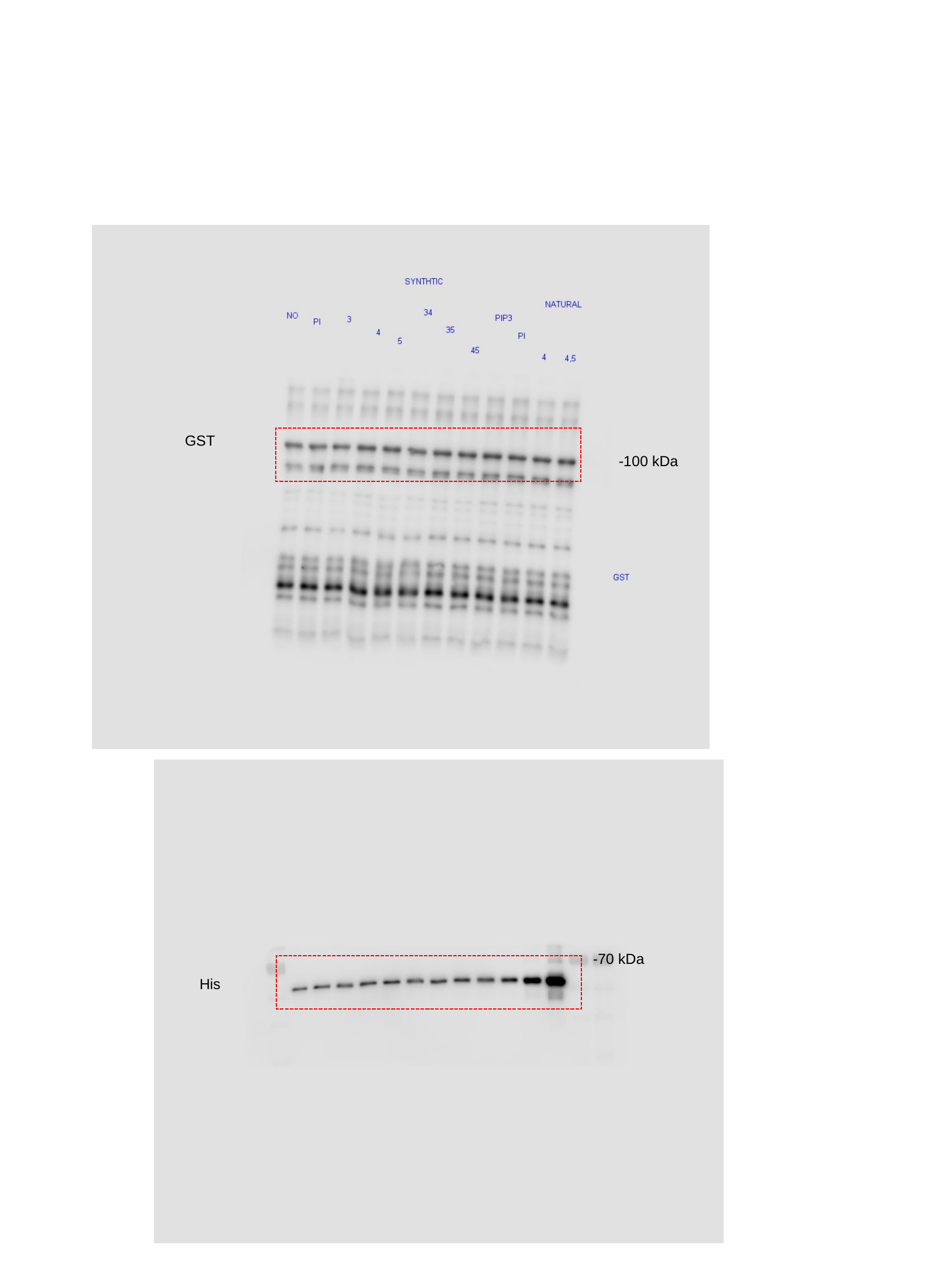

GST
-100 kDa
-70 kDa
His

Supplement: Supplementary file 6 — Source data Fig. 5 [file 44318_2024_85_MOESM6_ESM.zip › SD Figure 5/5B.pptx]

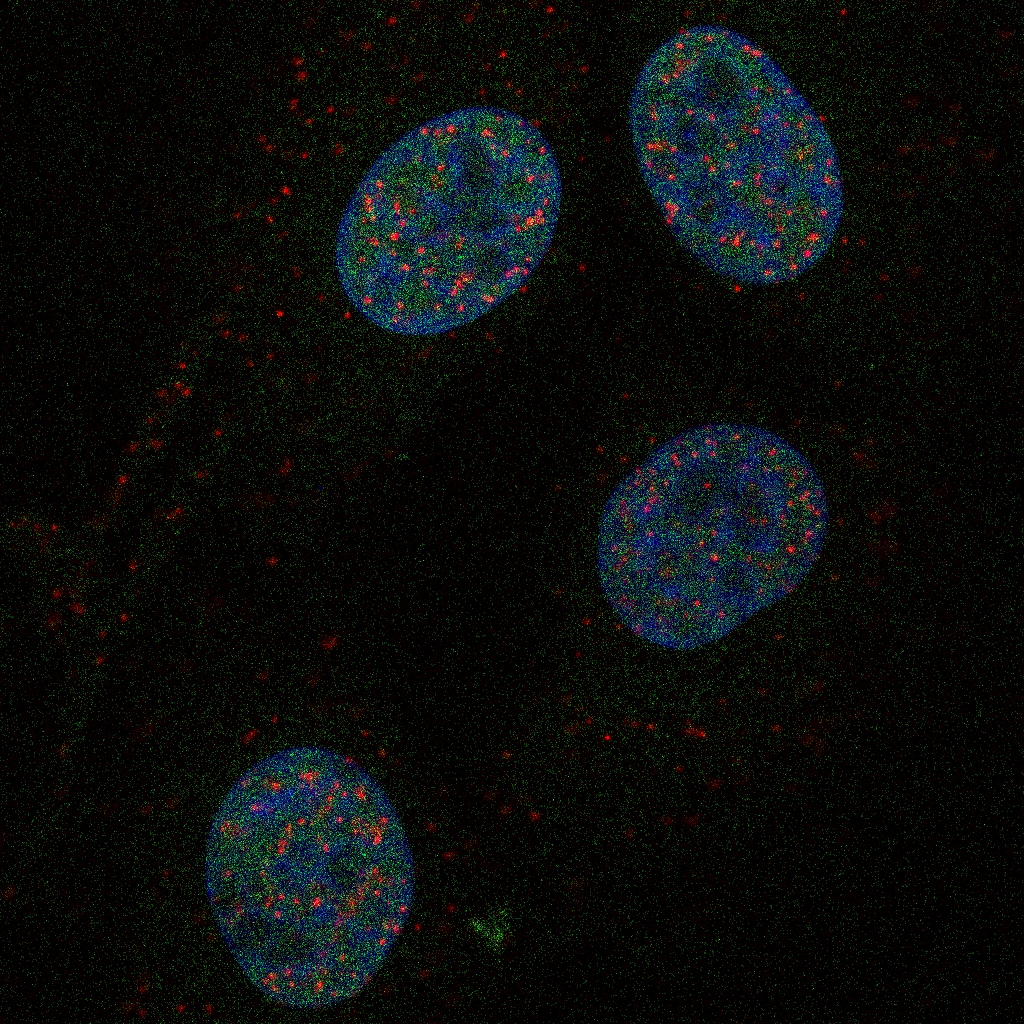

Supplement: Supplementary file 6 — Source data Fig. 5 [file 44318_2024_85_MOESM6_ESM.zip › SD Figure 5/5G high resolution/Merge+DAPI.jpg]

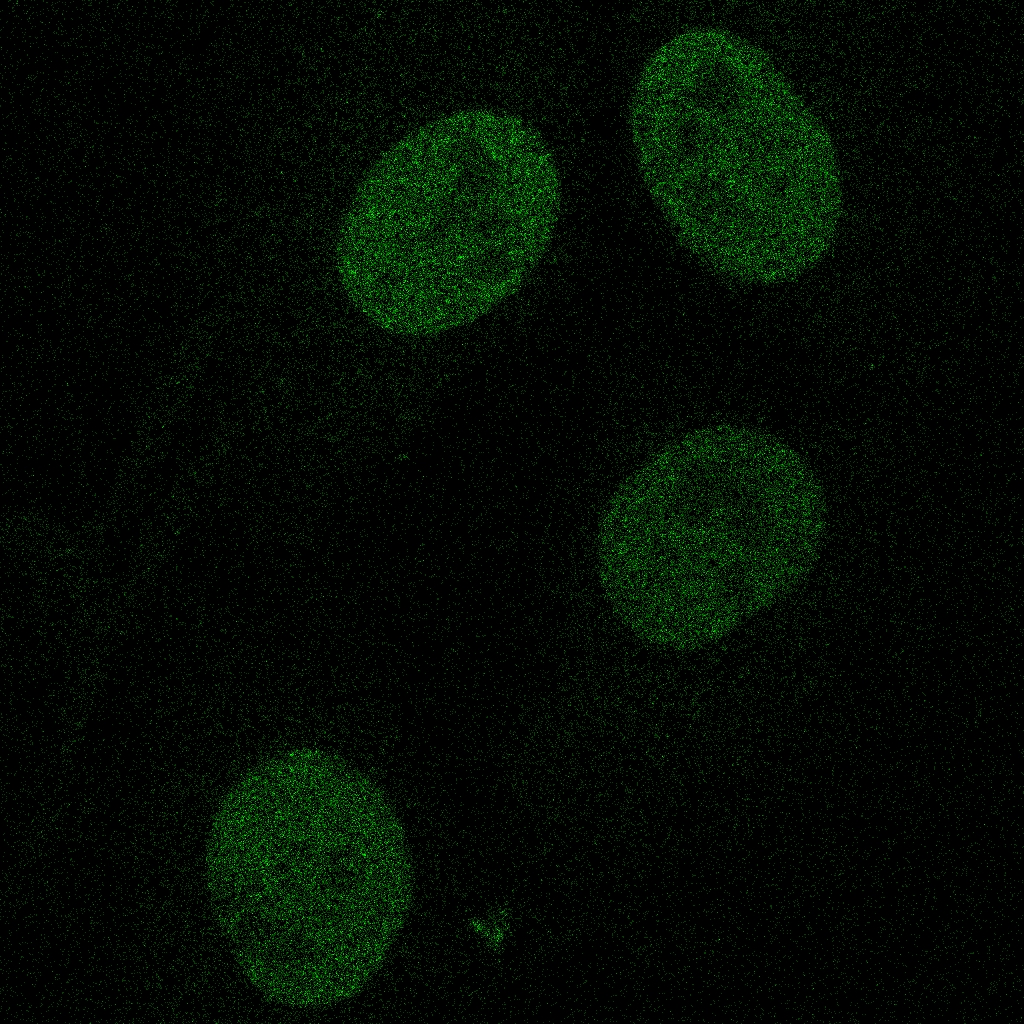

Supplement: Supplementary file 6 — Source data Fig. 5 [file 44318_2024_85_MOESM6_ESM.zip › SD Figure 5/5G high resolution/TEAD1.jpg]

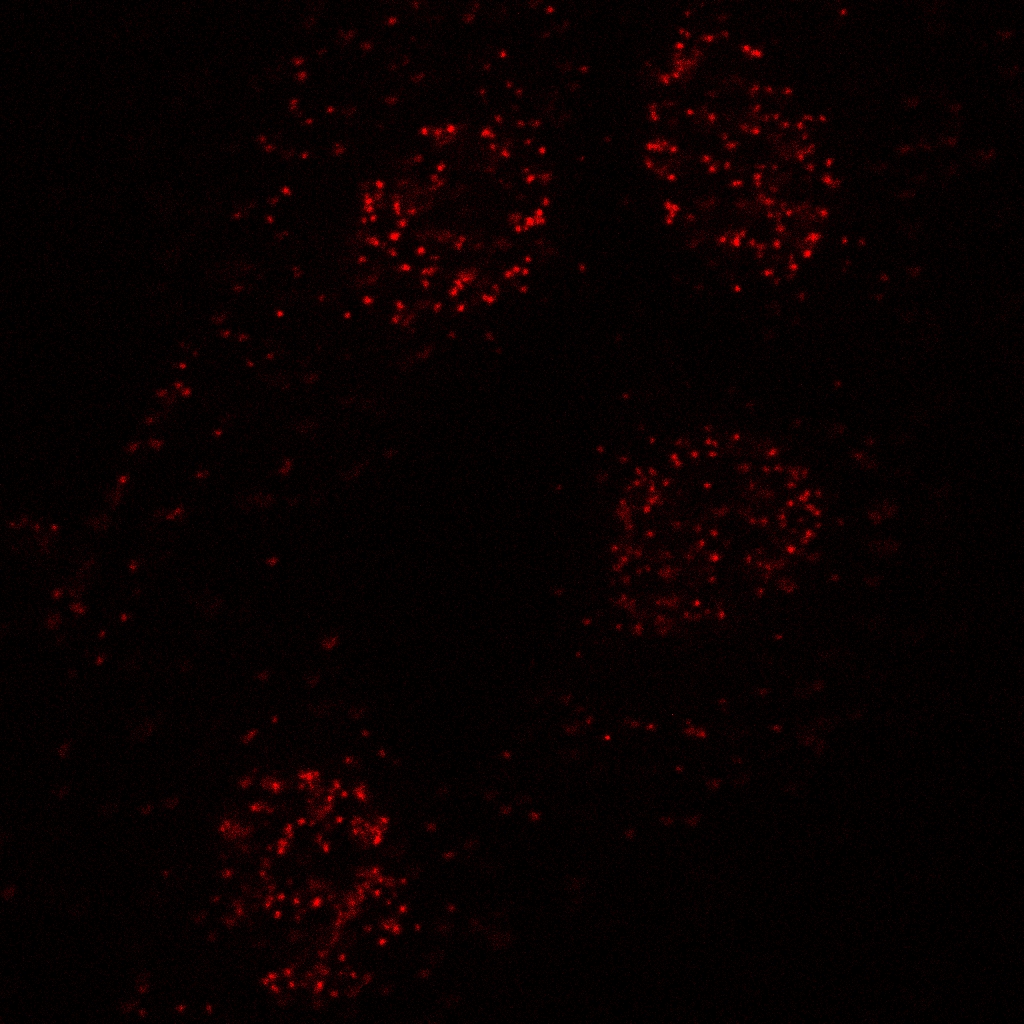

Supplement: Supplementary file 6 — Source data Fig. 5 [file 44318_2024_85_MOESM6_ESM.zip › SD Figure 5/5G high resolution/PLA.jpg]

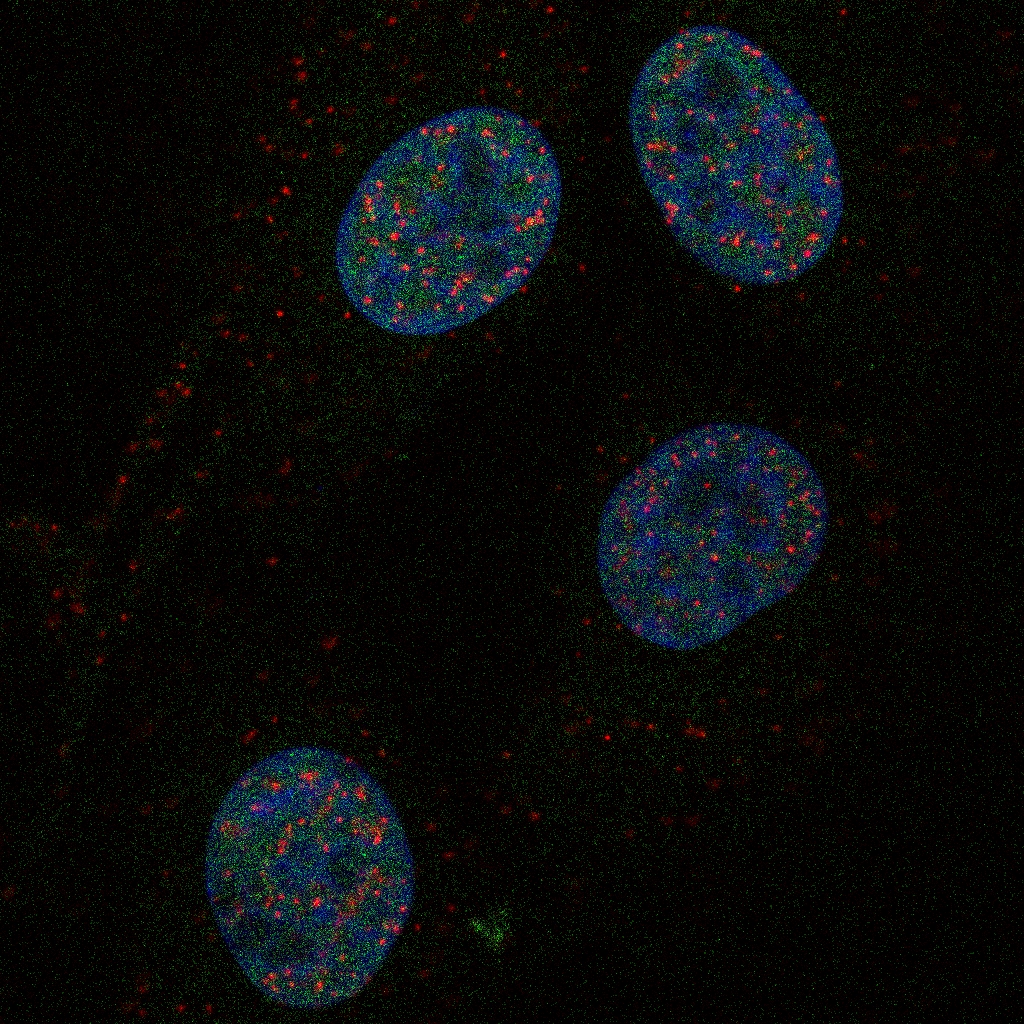

Supplement: Supplementary file 6 — Source data Fig. 5 [file 44318_2024_85_MOESM6_ESM.zip › SD Figure 5/5G high resolution/Merge.jpg]

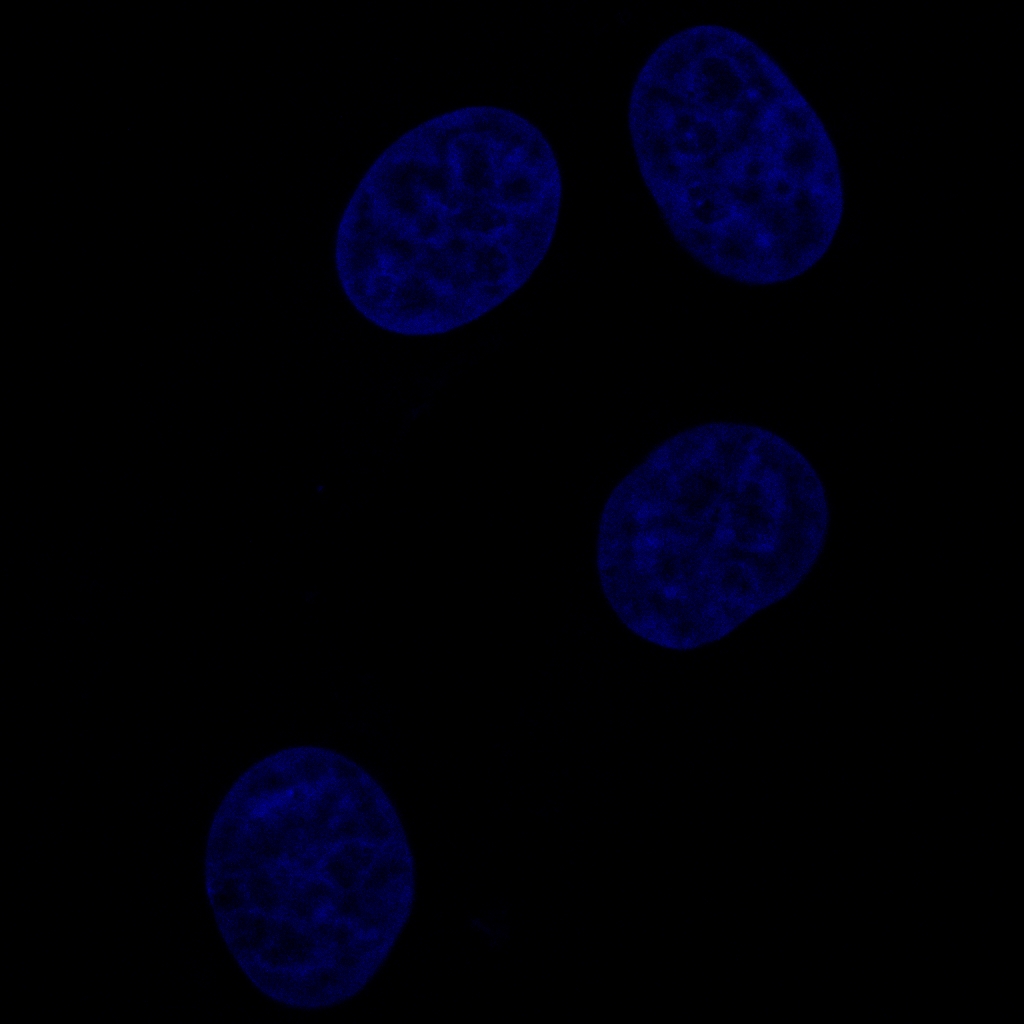

Supplement: Supplementary file 6 — Source data Fig. 5 [file 44318_2024_85_MOESM6_ESM.zip › SD Figure 5/5G high resolution/DAPI.jpg]

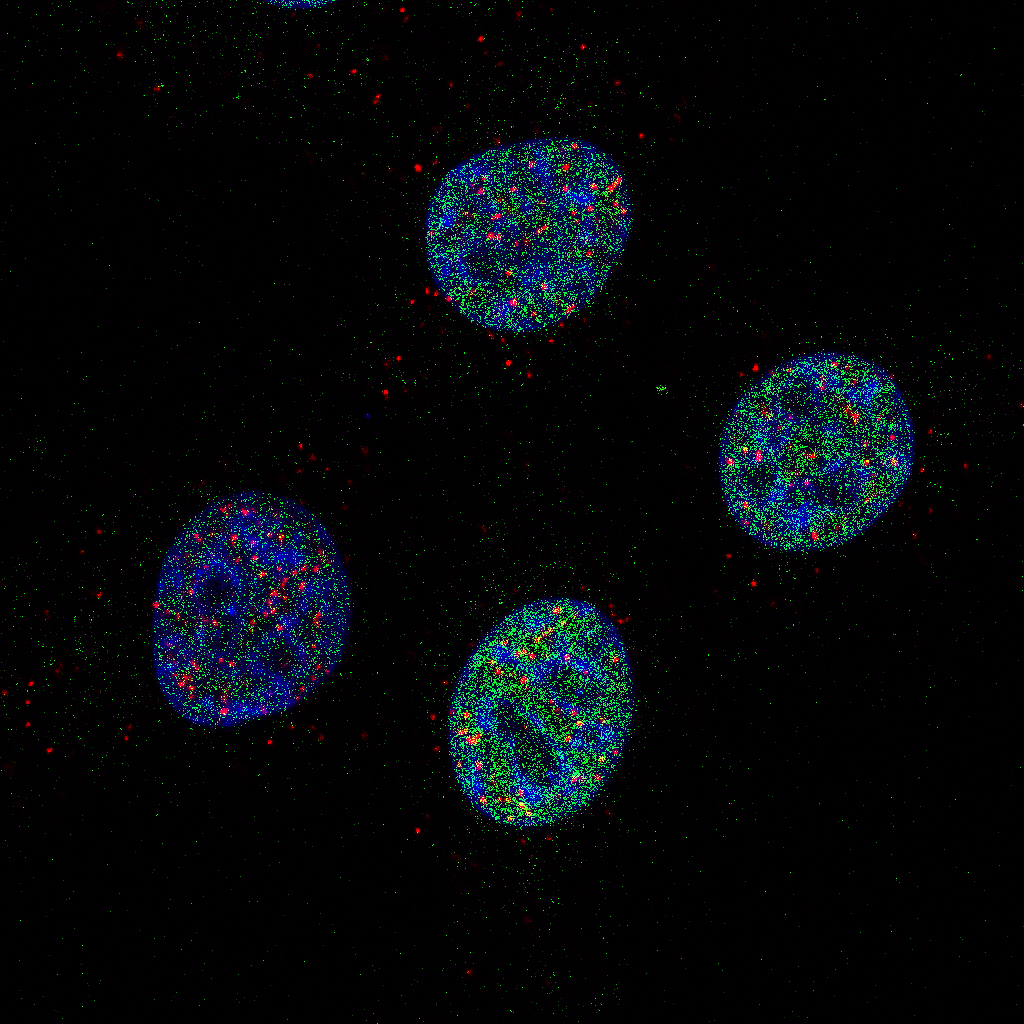

Supplement: Supplementary file 6 — Source data Fig. 5 [file 44318_2024_85_MOESM6_ESM.zip › SD Figure 5/5H high resolution/Merge+DAPI.jpg]

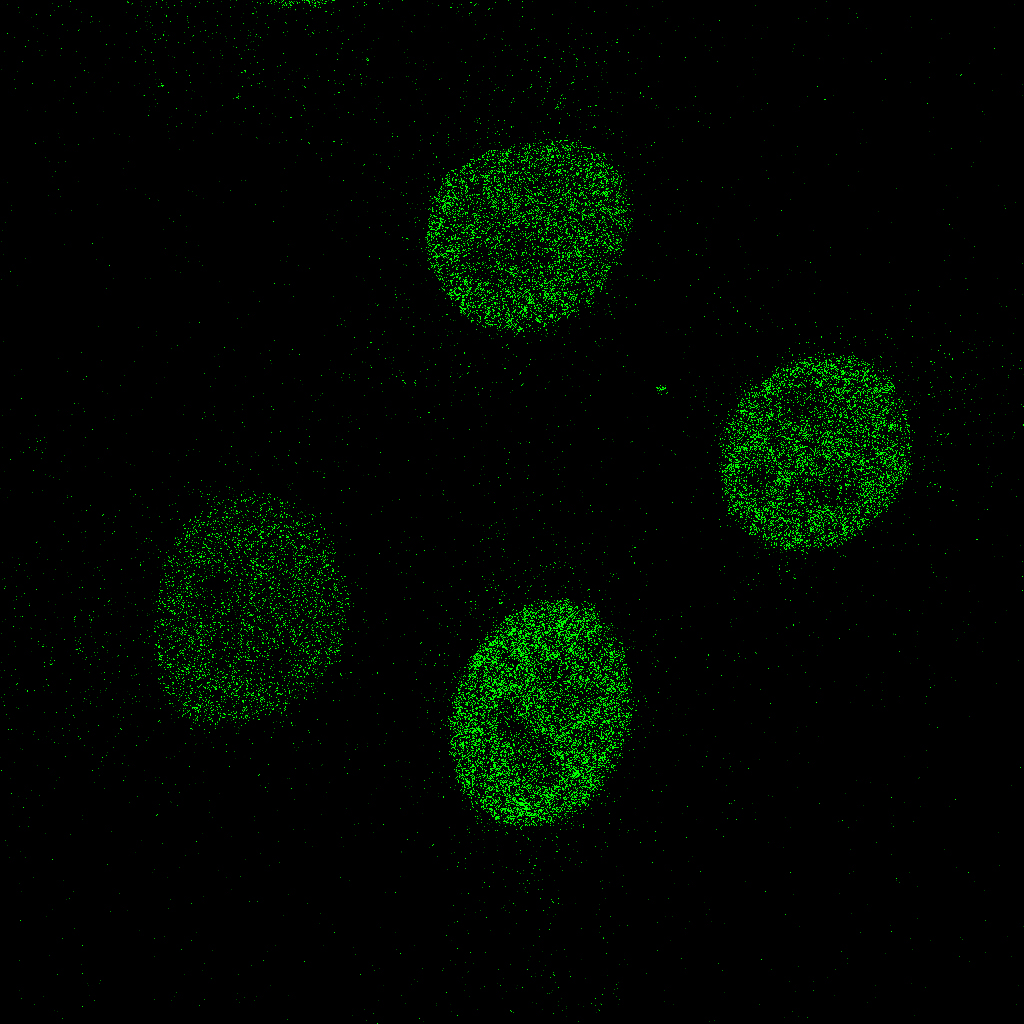

Supplement: Supplementary file 6 — Source data Fig. 5 [file 44318_2024_85_MOESM6_ESM.zip › SD Figure 5/5H high resolution/TEAD1.jpg]

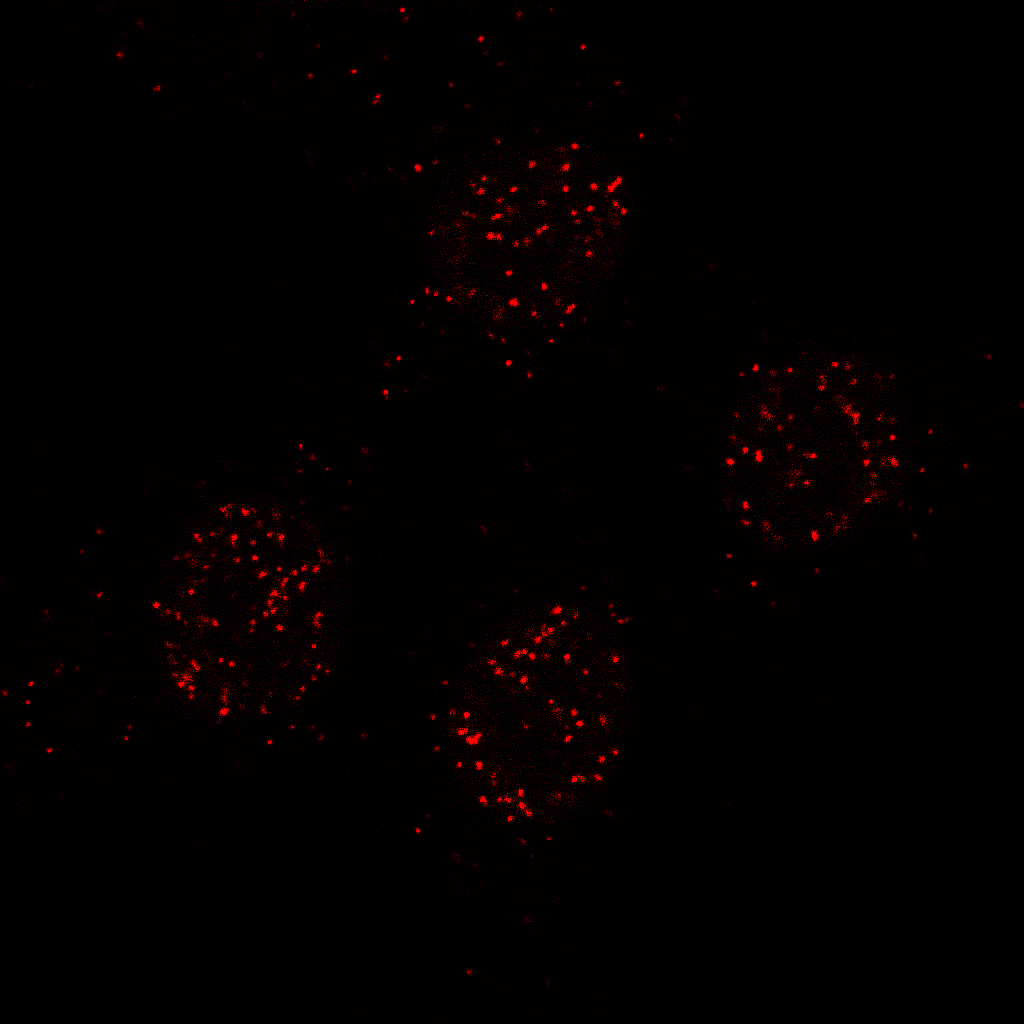

Supplement: Supplementary file 6 — Source data Fig. 5 [file 44318_2024_85_MOESM6_ESM.zip › SD Figure 5/5H high resolution/PLA.jpg]

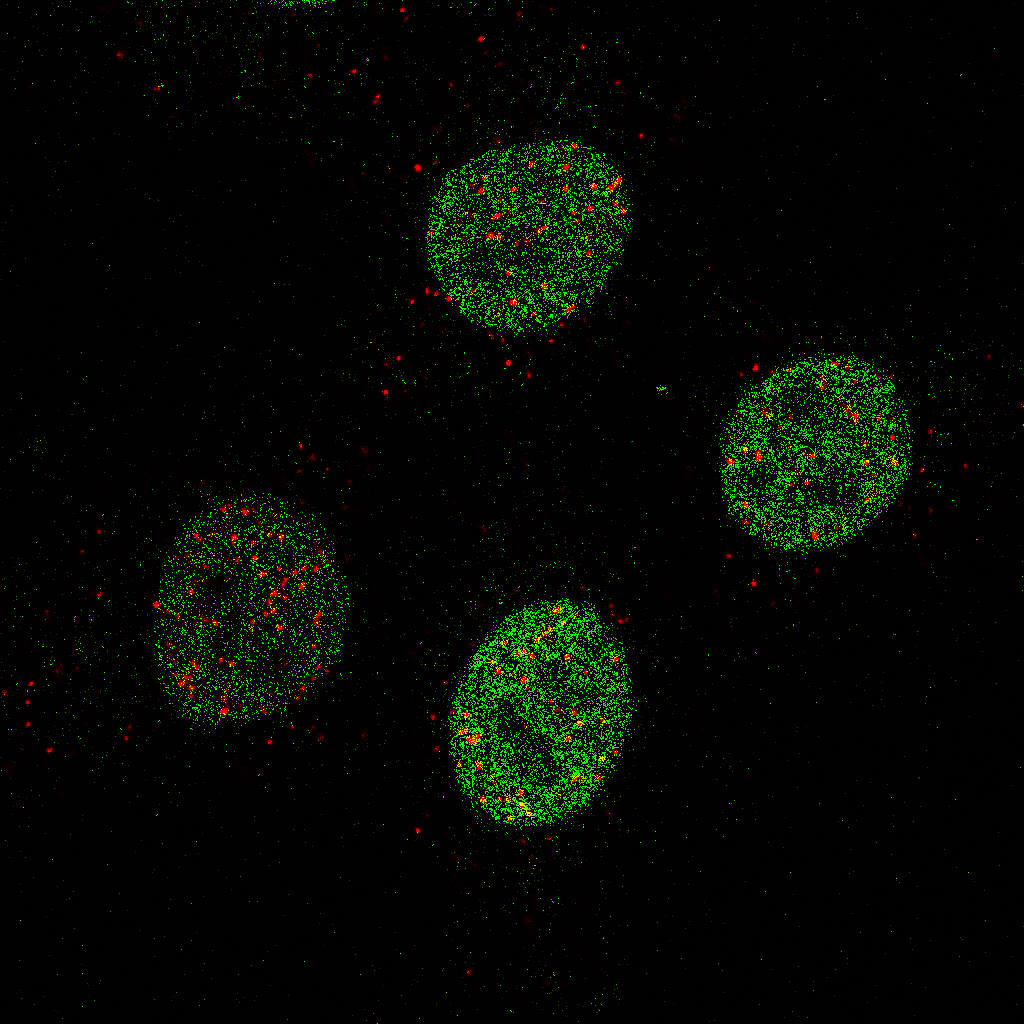

Supplement: Supplementary file 6 — Source data Fig. 5 [file 44318_2024_85_MOESM6_ESM.zip › SD Figure 5/5H high resolution/Merge.jpg]

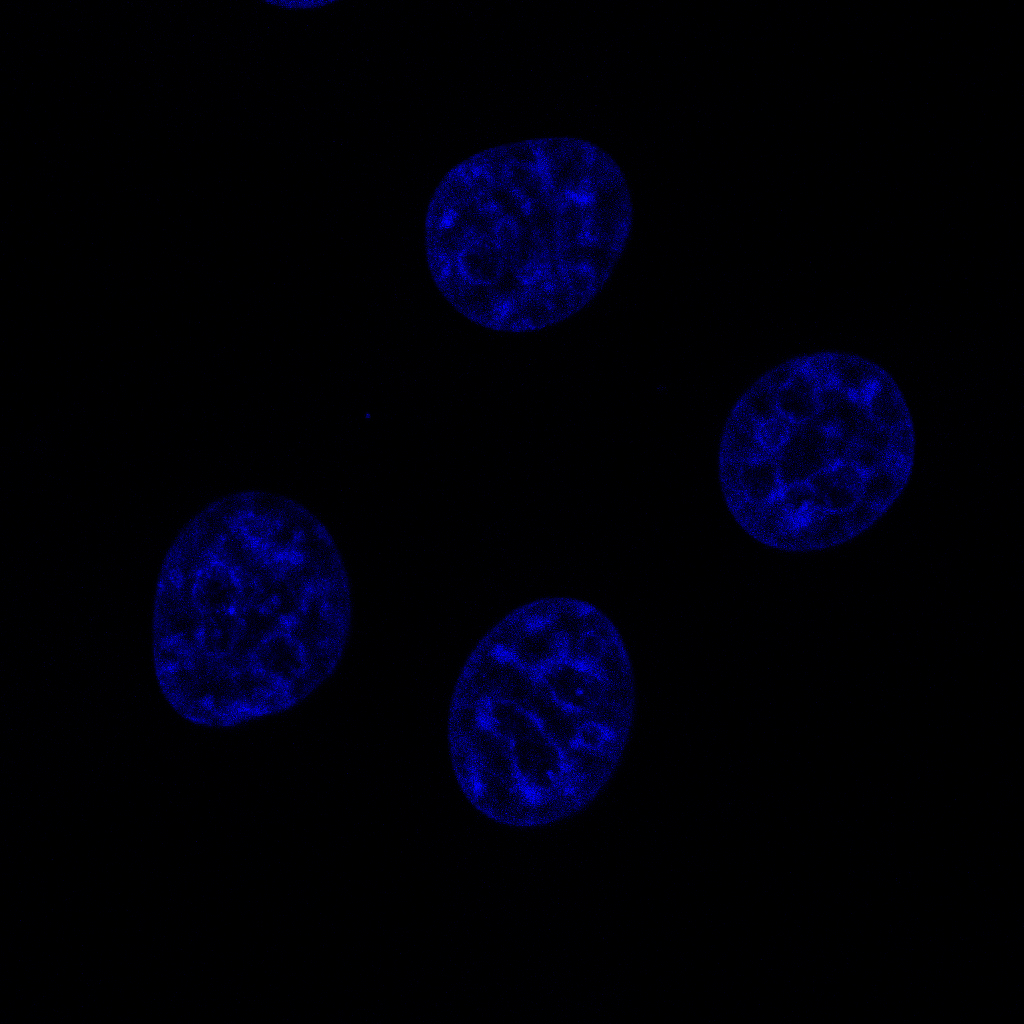

Supplement: Supplementary file 6 — Source data Fig. 5 [file 44318_2024_85_MOESM6_ESM.zip › SD Figure 5/5H high resolution/DAPI.jpg]

## Slide 1
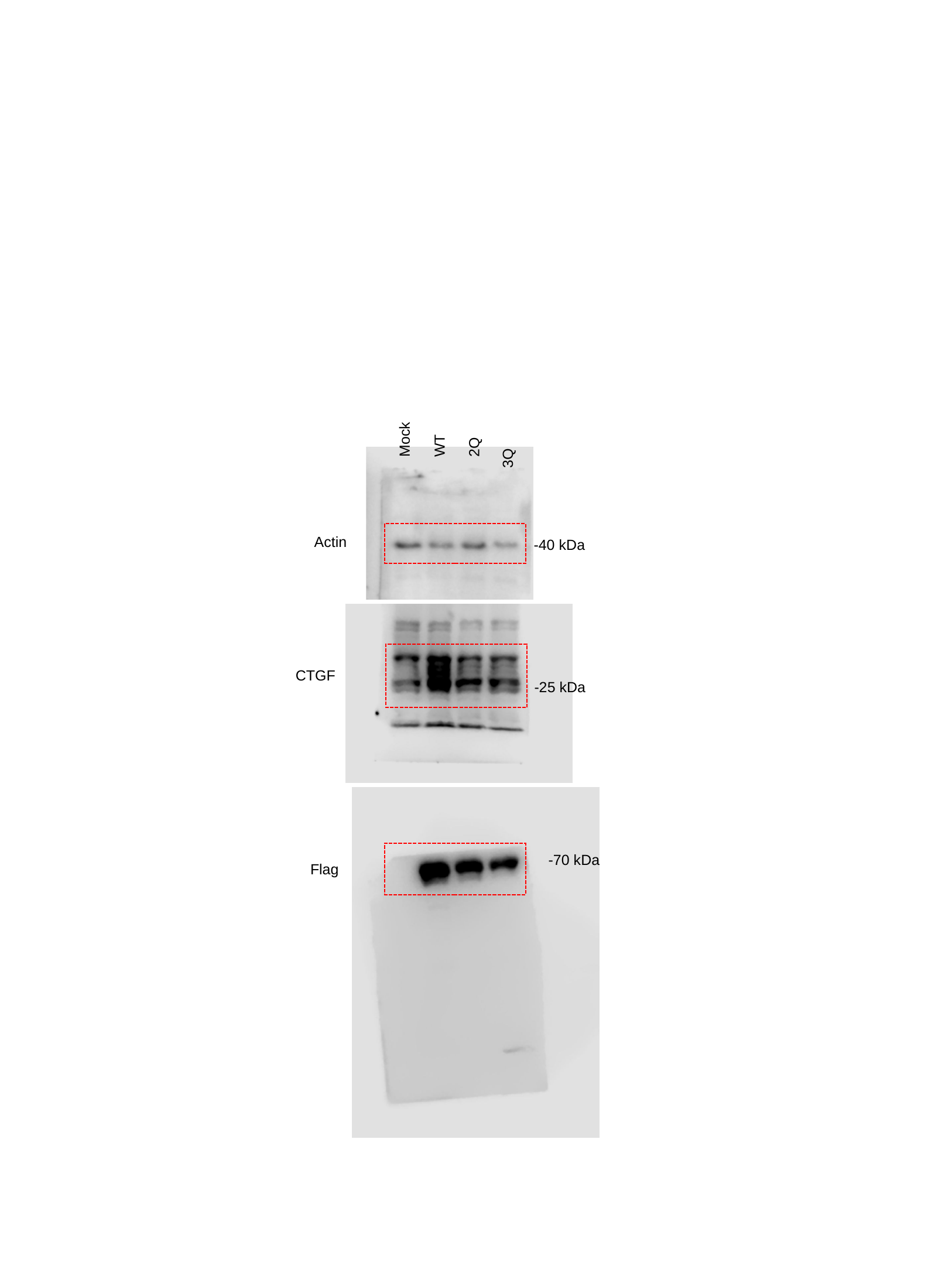

Mock
WT
2Q
3Q
Actin
-40 kDa
CTGF
-25 kDa
-70 kDa
Flag

Supplement: Supplementary file 7 — Source data Fig. 6 [file 44318_2024_85_MOESM7_ESM.zip › SD Figure 6/6F.pptx]

## Slide 1
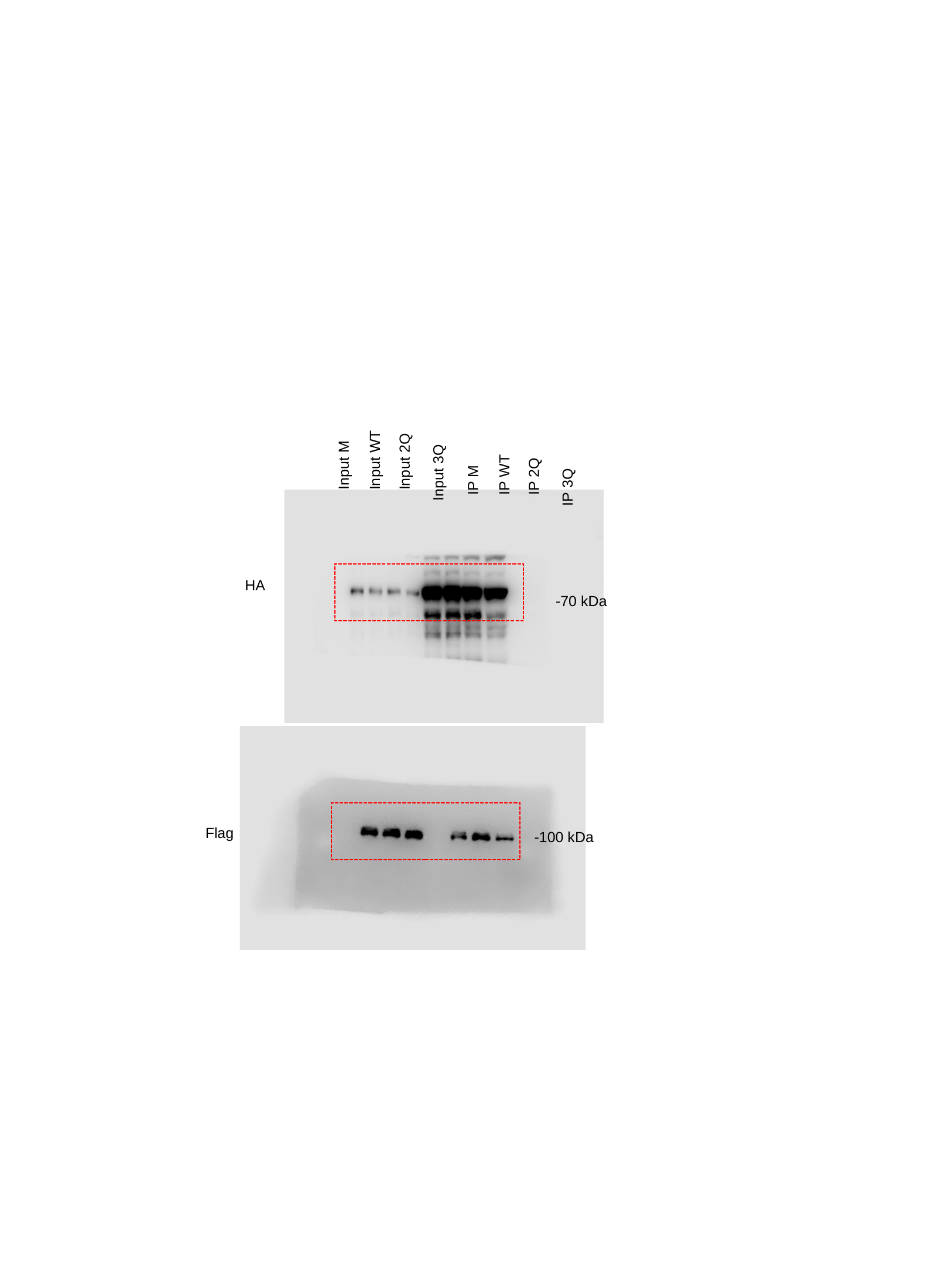

Input M
Input WT
Input 2Q
IP M
IP WT
IP 2Q
Input 3Q
IP 3Q
HA
-70 kDa
Flag
-100 kDa

Supplement: Supplementary file 7 — Source data Fig. 6 [file 44318_2024_85_MOESM7_ESM.zip › SD Figure 6/6E.pptx]

## Slide 1
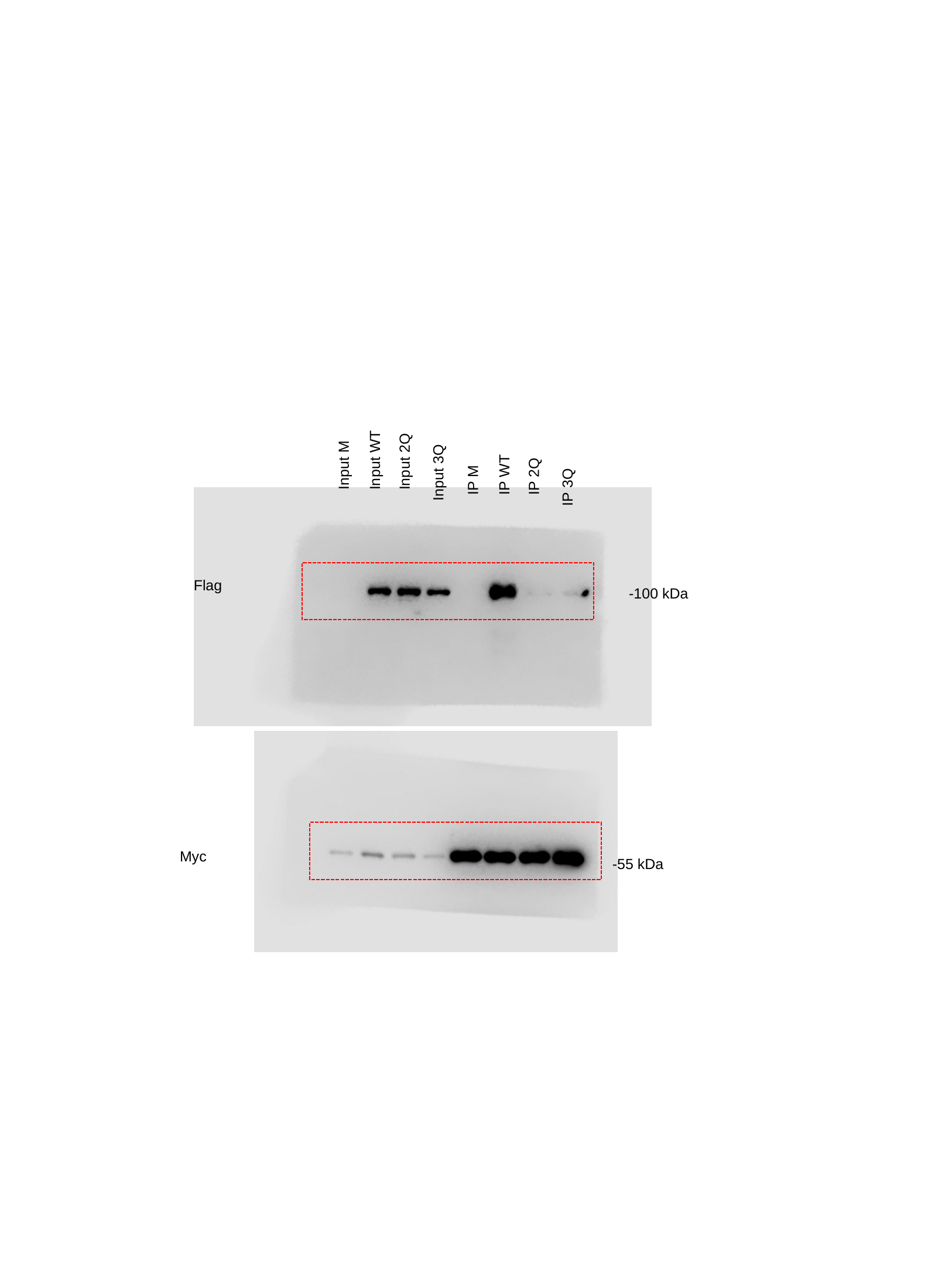

Input M
Input WT
Input 2Q
IP M
IP WT
IP 2Q
Input 3Q
IP 3Q
Flag
-100 kDa
Myc
-55 kDa

Supplement: Supplementary file 7 — Source data Fig. 6 [file 44318_2024_85_MOESM7_ESM.zip › SD Figure 6/6D.pptx]

## Slide 1
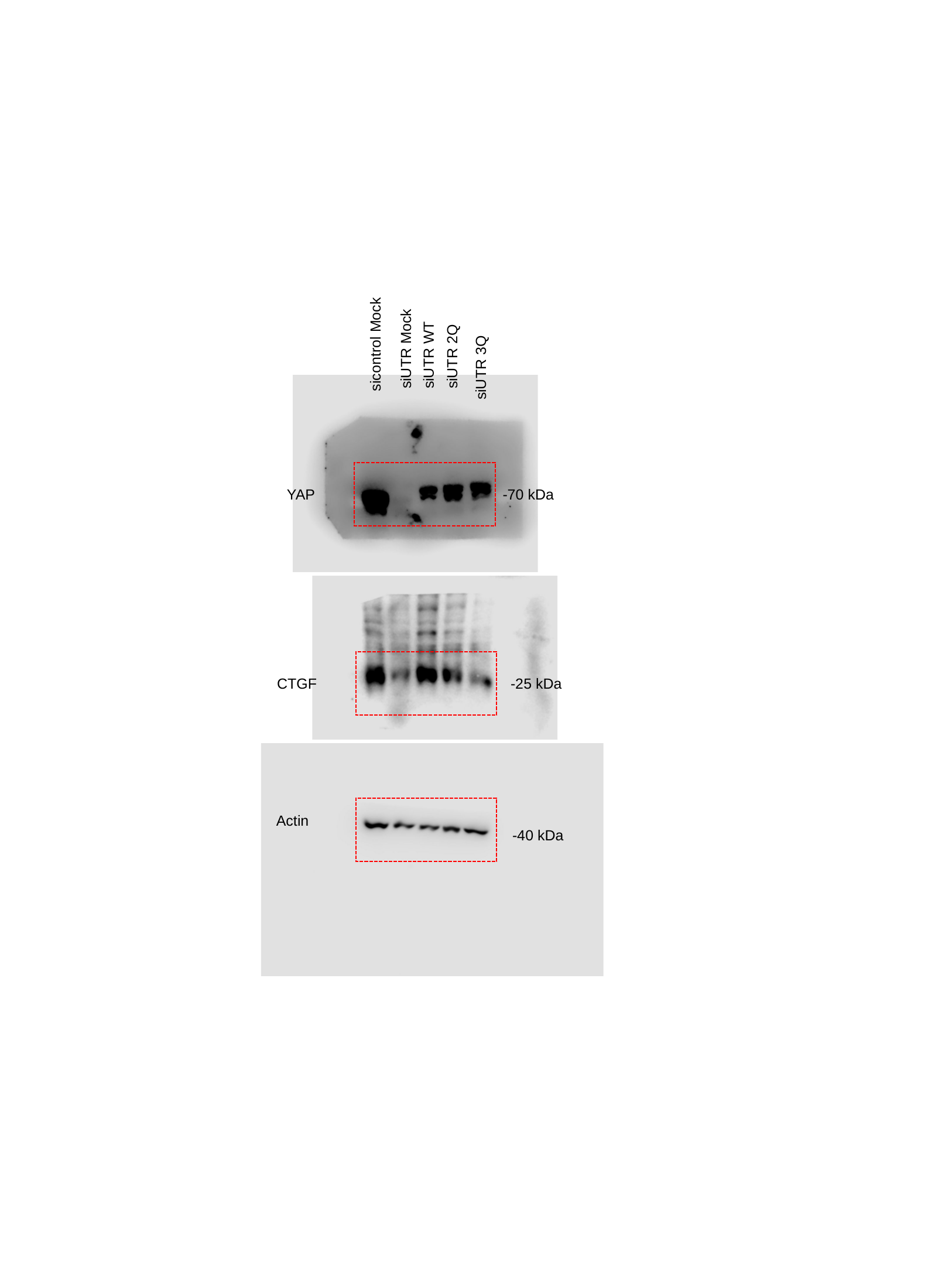

siUTR Mock
siUTR WT
siUTR 2Q
sicontrol Mock
siUTR 3Q
YAP
-70 kDa
CTGF
-25 kDa
Actin
-40 kDa

Supplement: Supplementary file 8 — Source data Fig. 7 [file 44318_2024_85_MOESM8_ESM.zip › SD Figure 7/7A.pptx]
