# Supplementary material for: Nuclear phosphoinositide signaling promotes YAP/TAZ-TEAD transcriptional activity in breast cancer
Source: EMBO J. 2024 Apr 2;43(9):4. doi: 10.1038/s44318-024-00085-6 (PMC11066040; doi:10.1038/s44318-024-00085-6)
Supplement: Supplementary file 4 — Source data Fig. 3 [file 44318_2024_85_MOESM4_ESM.zip › SD Figure 3/3F.pptx]

## Slide 1
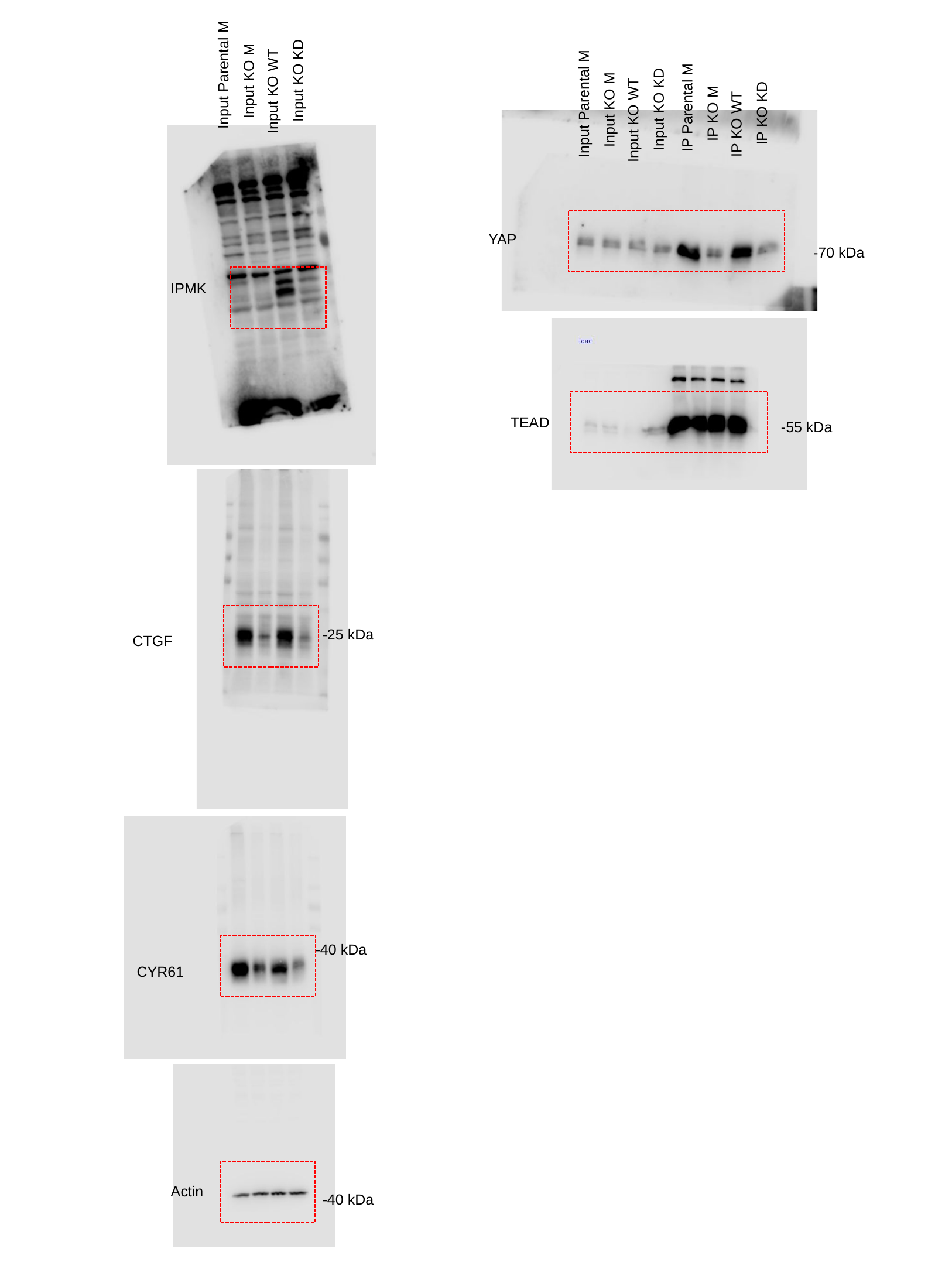

Input Parental M
Input KO KD
Input KO M
Input KO WT
Input Parental M
IP Parental M
Input KO KD
Input KO M
IP KO KD
IP KO M
Input KO WT
IP KO WT
YAP
-70 kDa
IPMK
TEAD
-55 kDa
-25 kDa
CTGF
-40 kDa
CYR61
Actin
-40 kDa
