# Supplementary material for: Nuclear phosphoinositide signaling promotes YAP/TAZ-TEAD transcriptional activity in breast cancer
Source: EMBO J. 2024 Apr 2;43(9):4. doi: 10.1038/s44318-024-00085-6 (PMC11066040; doi:10.1038/s44318-024-00085-6)
Supplement: Supplementary file 4 — Source data Fig. 3 [file 44318_2024_85_MOESM4_ESM.zip › SD Figure 3/3E.pptx]

## Slide 1
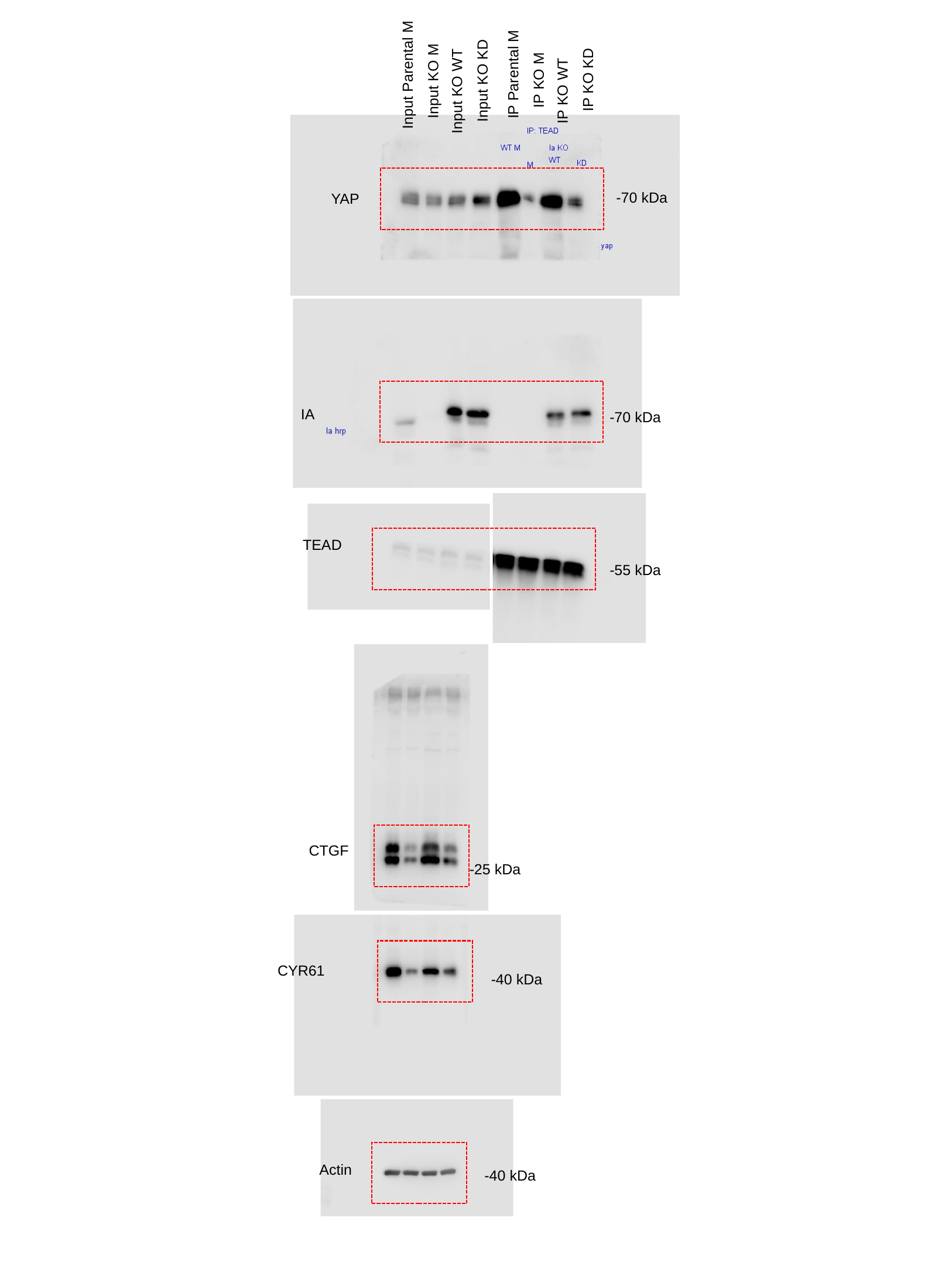

IP Parental M
Input Parental M
IP KO KD
IP KO M
Input KO KD
Input KO M
IP KO WT
Input KO WT
-70 kDa
YAP
IA
-70 kDa
TEAD
-55 kDa
CTGF
-25 kDa
CYR61
-40 kDa
Actin
-40 kDa
