# Supplementary material for: Nuclear phosphoinositide signaling promotes YAP/TAZ-TEAD transcriptional activity in breast cancer
Source: EMBO J. 2024 Apr 2;43(9):4. doi: 10.1038/s44318-024-00085-6 (PMC11066040; doi:10.1038/s44318-024-00085-6)
Supplement: Supplementary file 4 — Source data Fig. 3 [file 44318_2024_85_MOESM4_ESM.zip › SD Figure 3/3B.pptx]

## Slide 1
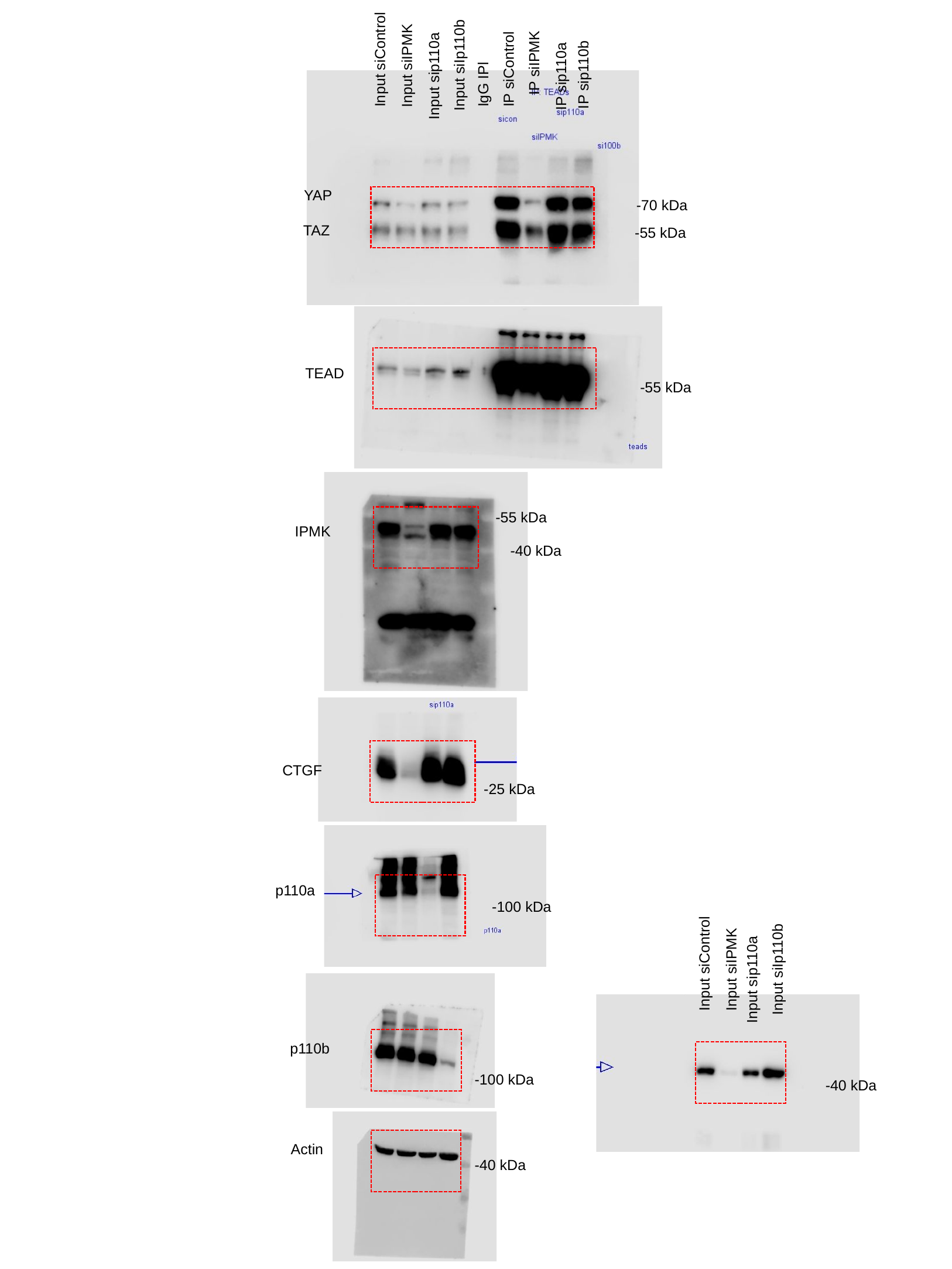

Input siControl
IP siIPMK
Input siIp110b
Input siIPMK
IP siControl
IP sip110b
Input sip110a
IP sip110a
IgG IPl
YAP
-70 kDa
TAZ
-55 kDa
TEAD
-55 kDa
-55 kDa
IPMK
-40 kDa
CTGF
-25 kDa
p110a
-100 kDa
Input siControl
Input siIp110b
Input siIPMK
Input sip110a
p110b
-100 kDa
-40 kDa
Actin
-40 kDa
