# Supplementary material for: Nuclear phosphoinositide signaling promotes YAP/TAZ-TEAD transcriptional activity in breast cancer
Source: EMBO J. 2024 Apr 2;43(9):4. doi: 10.1038/s44318-024-00085-6 (PMC11066040; doi:10.1038/s44318-024-00085-6)
Supplement: Supplementary file 5 — Source data Fig. 4 [file 44318_2024_85_MOESM5_ESM.zip › SD Figure 4/4B.pptx]

## Slide 1
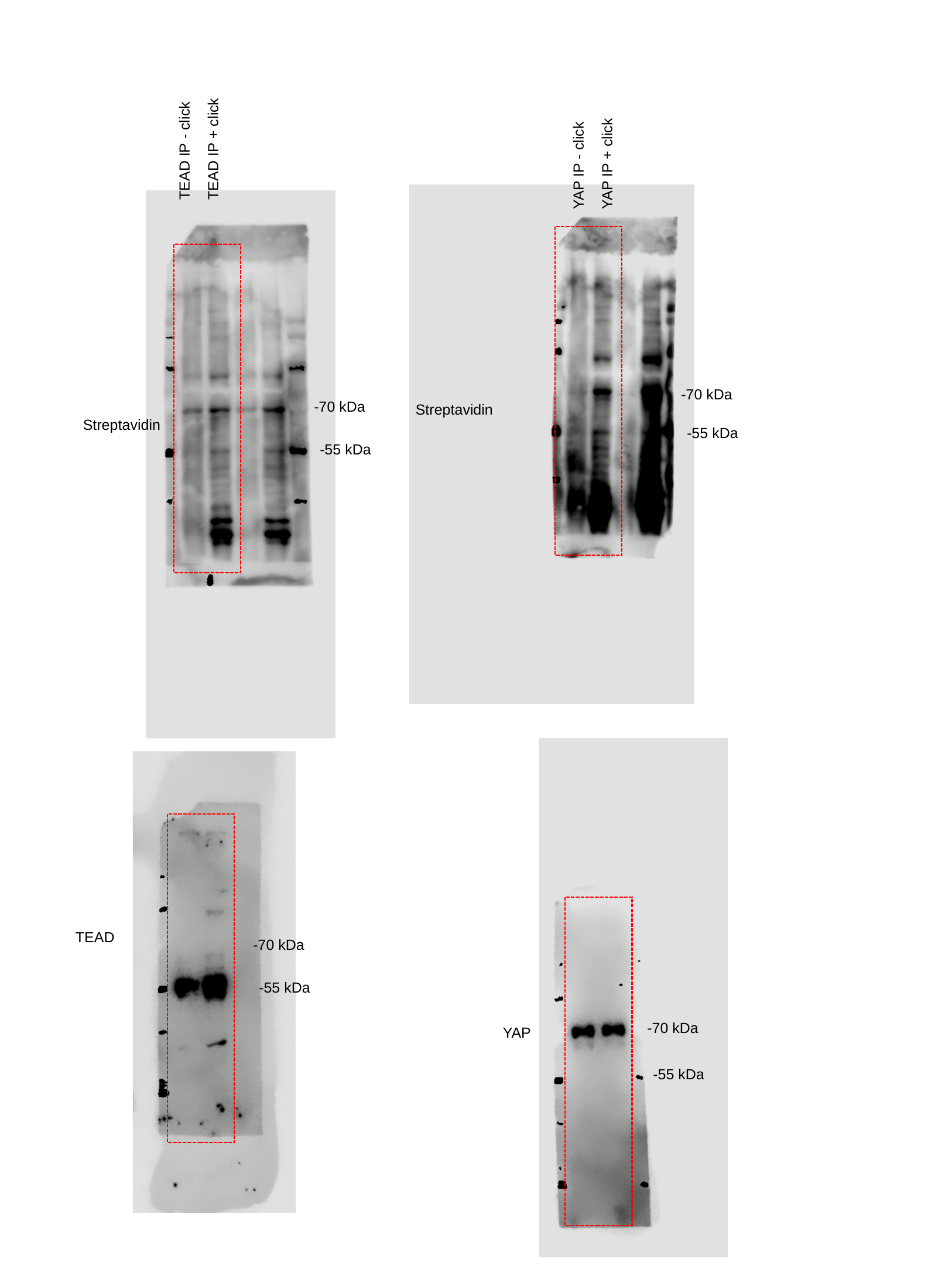

TEAD IP + click
TEAD IP - click
YAP IP + click
YAP IP - click
-70 kDa
-70 kDa
Streptavidin
Streptavidin
-55 kDa
-55 kDa
TEAD
-70 kDa
-55 kDa
-70 kDa
YAP
-55 kDa
