# Supplementary material for: Nuclear phosphoinositide signaling promotes YAP/TAZ-TEAD transcriptional activity in breast cancer
Source: EMBO J. 2024 Apr 2;43(9):4. doi: 10.1038/s44318-024-00085-6 (PMC11066040; doi:10.1038/s44318-024-00085-6)
Supplement: Supplementary file 5 — Source data Fig. 4 [file 44318_2024_85_MOESM5_ESM.zip › SD Figure 4/4I.pptx]

## Slide 1
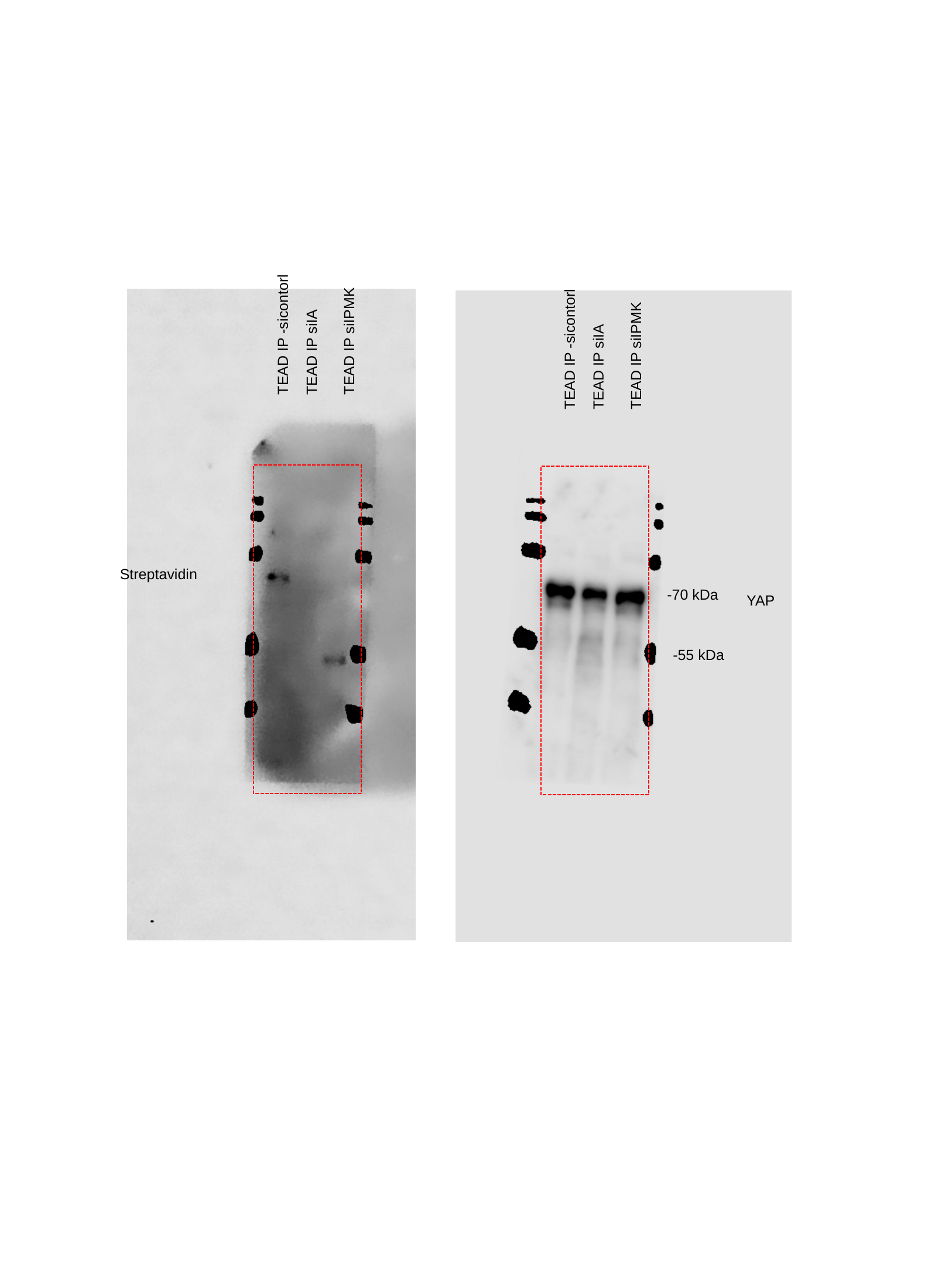

TEAD IP -sicontorl
TEAD IP siIPMK
TEAD IP -sicontorl
TEAD IP siIA
TEAD IP siIPMK
TEAD IP siIA
Streptavidin
-70 kDa
YAP
-55 kDa
