# Supplementary material for: Nuclear phosphoinositide signaling promotes YAP/TAZ-TEAD transcriptional activity in breast cancer
Source: EMBO J. 2024 Apr 2;43(9):4. doi: 10.1038/s44318-024-00085-6 (PMC11066040; doi:10.1038/s44318-024-00085-6)
Supplement: Supplementary file 7 — Source data Fig. 6 [file 44318_2024_85_MOESM7_ESM.zip › SD Figure 6/6C.pptx]

## Slide 1
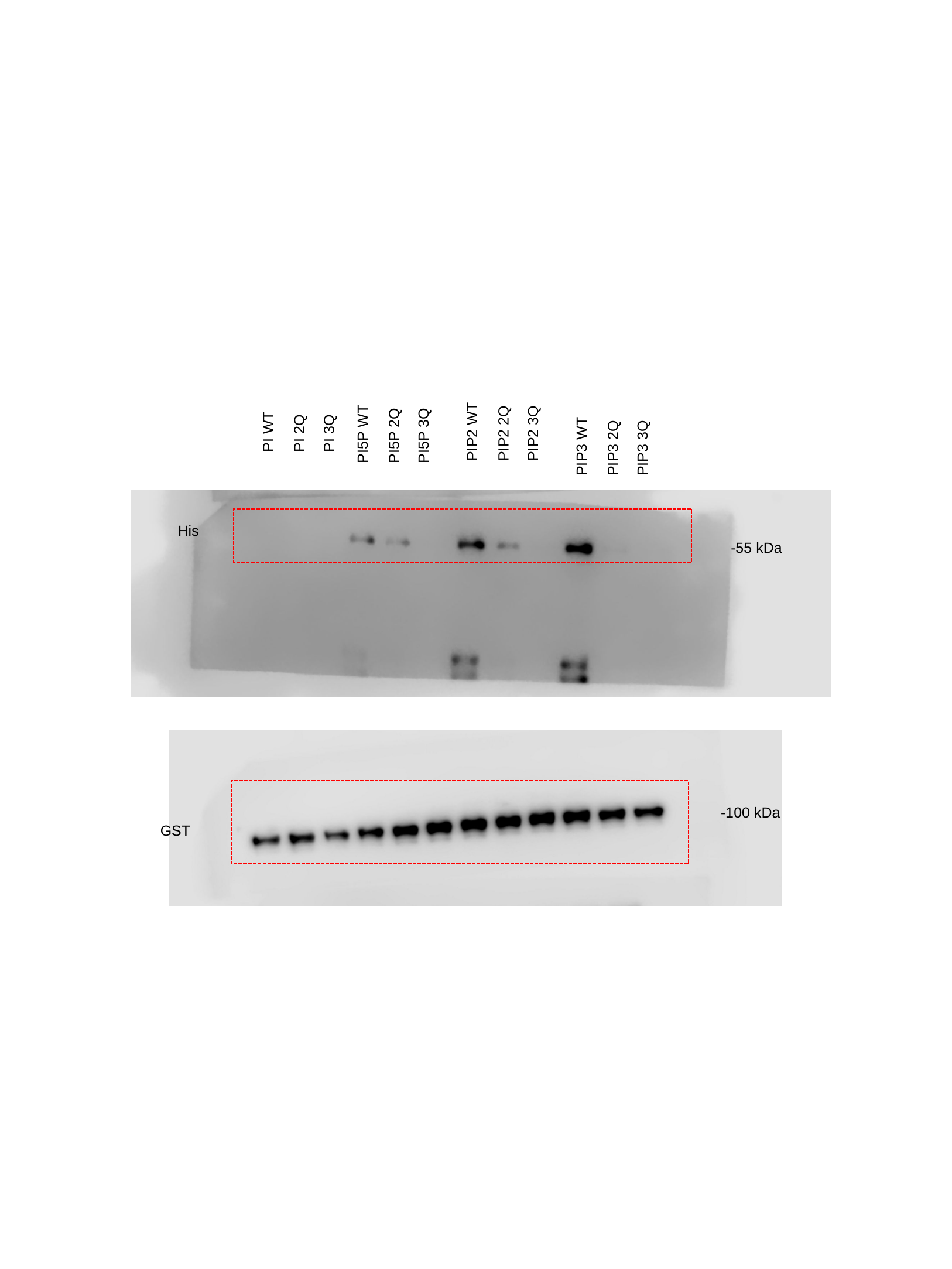

PI WT
PI 2Q
PI 3Q
PIP2 WT
PIP2 2Q
PIP2 3Q
PI5P WT
PI5P 2Q
PI5P 3Q
PIP3 WT
PIP3 2Q
PIP3 3Q
PIP2 GST-1Q
PIP3 GST-WT
PIP3 GST-3Q
PIP3 GST-2Q
PIP3 GST-1Q
His
-55 kDa
-100 kDa
GST
