# Supplementary material for: Nuclear phosphoinositide signaling promotes YAP/TAZ-TEAD transcriptional activity in breast cancer
Source: EMBO J. 2024 Apr 2;43(9):4. doi: 10.1038/s44318-024-00085-6 (PMC11066040; doi:10.1038/s44318-024-00085-6)
Supplement: Supplementary file 7 — Source data Fig. 6 [file 44318_2024_85_MOESM7_ESM.zip › SD Figure 6/6B.pptx]

## Slide 1
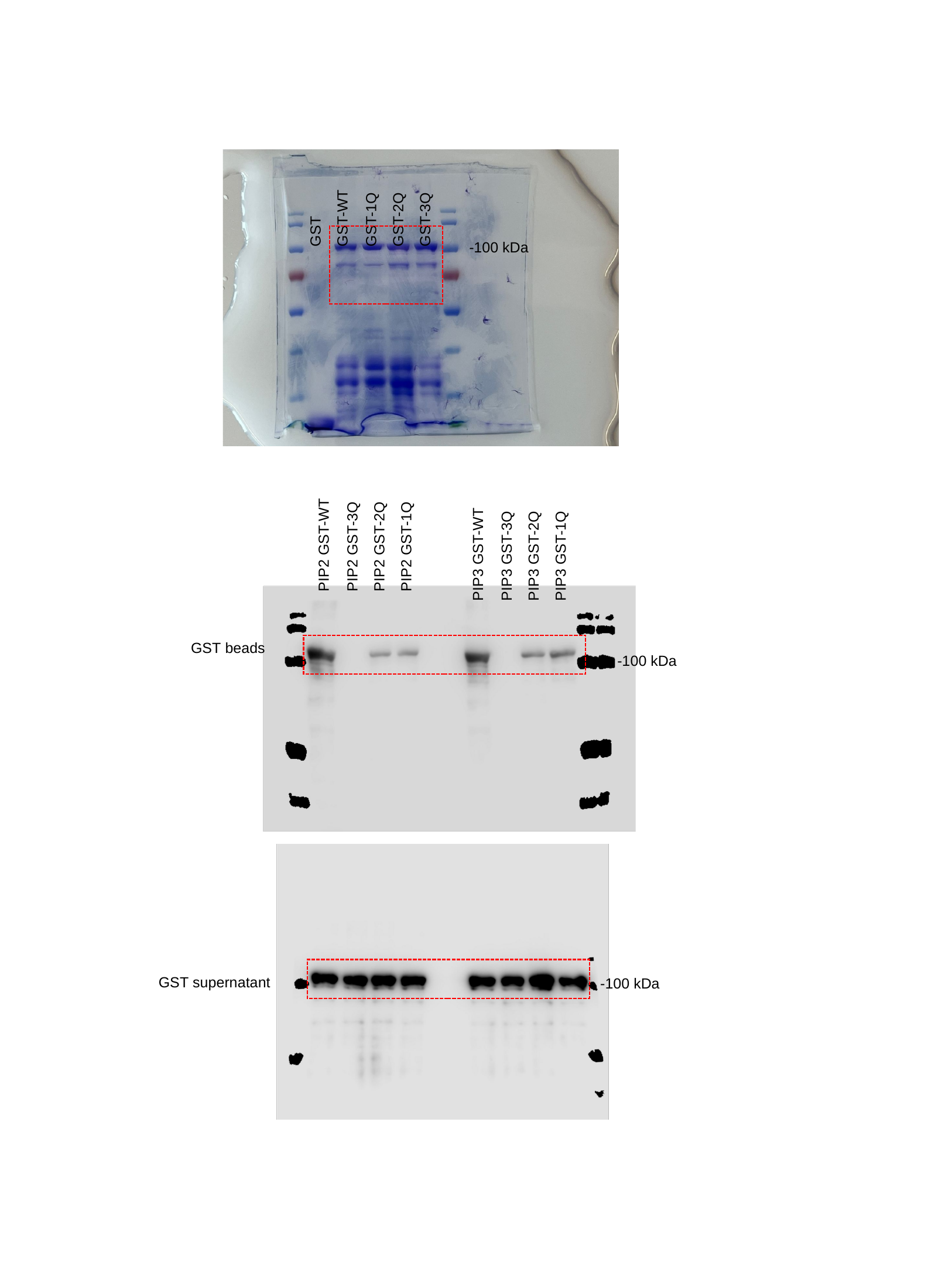

GST
GST-WT
GST-1Q
GST-2Q
GST-3Q
-100 kDa
PIP2 GST-WT
PIP2 GST-3Q
PIP2 GST-2Q
PIP2 GST-1Q
PIP3 GST-WT
PIP3 GST-3Q
PIP3 GST-2Q
PIP3 GST-1Q
GST beads
-100 kDa
GST supernatant
-100 kDa
