# Supplementary material for: Nuclear phosphoinositide signaling promotes YAP/TAZ-TEAD transcriptional activity in breast cancer
Source: EMBO J. 2024 Apr 2;43(9):4. doi: 10.1038/s44318-024-00085-6 (PMC11066040; doi:10.1038/s44318-024-00085-6)
Supplement: Supplementary file 10 — Expanded View Figures [file 44318_2024_85_MOESM10_ESM.pdf]

Expanded View Figures

Figure EV1. PIPKIα and IPMK are required for the expression of YAP/TAZ target genes, and agonist stimulation increases YAP associations with PIPKIα and IPMK in triple-negative breast cancer cells.

(**A**) PIPKIα and IPMK were transiently knocked down in MDA-MB-468 cells, another triple-negative cell line, by transfecting siRNA for 72 h and the expression of several proteins was analyzed by immunoblotting. Representative immunoblot images of $n = 3$ independent experiments are shown. The expression of CTGF and CYR61 but not another known YAP/TAZ target AXL was reduced by PIPKIα or IPMK knockdown. (**B**) A YAP/TAZ–TEAD firefly luciferase reporter construct along with a *Renilla* luciferase construct were transfected in MDA-MB-231 cells 24 h after siRNA transfection against PIPKIα and IPMK. After another 48 h incubation, firefly and *Renilla* luciferase activities were measured and the graph is shown as mean ± s.d. of $n = 3$ independent experiments. PIPKIα or IPMK knockdown significantly reduced the activity of the YAP/TAZ–TEAD promoter. *$P < 0.05$; **$P < 0.01$, and n.s.; not significant in Student's *t* test. (**C**, **D**) In all, 0.1 μM GST alone and GST-YAP recombinant proteins were incubated with 0.05 μM His_6-tagged recombinant PIPKIα (**C**) or IPMK (**D**). YAP proteins were pulled down with glutathione beads and the associated PIPKIα and IPMK were analyzed with immunoblotting. Representative immunoblot images of $n = 3$ independent experiments are shown. These results show YAP can directly interact with PIPKIα and IPMK. (**E**) Schematic representations of WT vs. a mutant YAP (mtWW) which contains inactivating mutations in the WW domains are shown. TBD TEAD-binding domain, TAD transactivation domain. (**F**) A schematic representation of the modules involved in the interactions of YAP/TAZ with PIPKIα and IPMK is shown. (**G**) Serum-starved MDA-MB-231 cells were treated with 10% serum or 5 μM LPA for 1 h. Cells were lysed and endogenous YAP was immunoprecipitated and the associated endogenous PIPKIα and IPMK were analyzed by immunoblotting. Representative immunoblot images of $n = 3$ independent experiments are shown. Treating the cells with serum or LPA increased the association of YAP with PIPKIα and IPMK. (**H**) MDA-MB-231 cells grown in 10% serum were treated with the indicated agonists for 90 min. Cells were lysed, endogenous YAP was immunoprecipitated, and the associated proteins were analyzed by immunoblotting. Representative immunoblot images of $n = 3$ independent experiments are shown. Treating the cells with any of the three agonists increased the association of YAP with PIPKIα and IPMK. (**I**) HEK293 cells grown in 10% serum were treated with 5 μM LPA for 2 h. The expression of endogenous YAP, phosphoS127 YAP, and CTGF was analyzed by immunoblotting. Representative immunoblot images of $n = 2$ independent experiments are shown. (**J**) Flag-YAP was co-transfected with HA-PIPKIα in HEK293 cells for 48 h. Cells grown in 10% serum were treated with 5 μM LPA for 2 h. Exogenous YAP was immunoprecipitated with an anti-Flag antibody and the associated exogenous PIPKIα was detected by immunoblotting with an anti-HA antibody. Representative immunoblot images of $n = 2$ independent experiments are shown. Treating the cells with LPA increased the association of PIPKIα with YAP. (**K**) Starved-parental or the indicated KO cells (pooled clones) were treated with 10% serum for 1 hr. Cells were lysed and the cell lysates were analyzed by immunoblotting with the indicated antibodies. Representative immunoblot images of $n = 2$ independent experiments are shown. KO of neither PIPKIα nor IPMK altered YAP phosphorylation at S127 residue. (**L**) Starved-parental or the indicated KO cells (pooled clones) were treated with 10% serum for 1 h. Cells were fixed and endogenous YAP localization was analyzed by immunofluorescence. Representative confocal images of $n = 2$ independent experiments are shown. Scale bar, 20 μm. (**M**) Starved-parental or YAP KO cells (pooled clones) were treated with 10% serum for 1 h. Endogenous TEAD was immunoprecipitated and the associated proteins were analyzed by immunoblotting with the indicated antibodies (top). Expression of the indicated proteins were analyzed by immunoblotting with the indicated antibodies (bottom). Representative immunoblot images of $n = 2$ independent experiments are shown. Serum treatment did not alter TEAD associations with PIPKIα and IPMK.

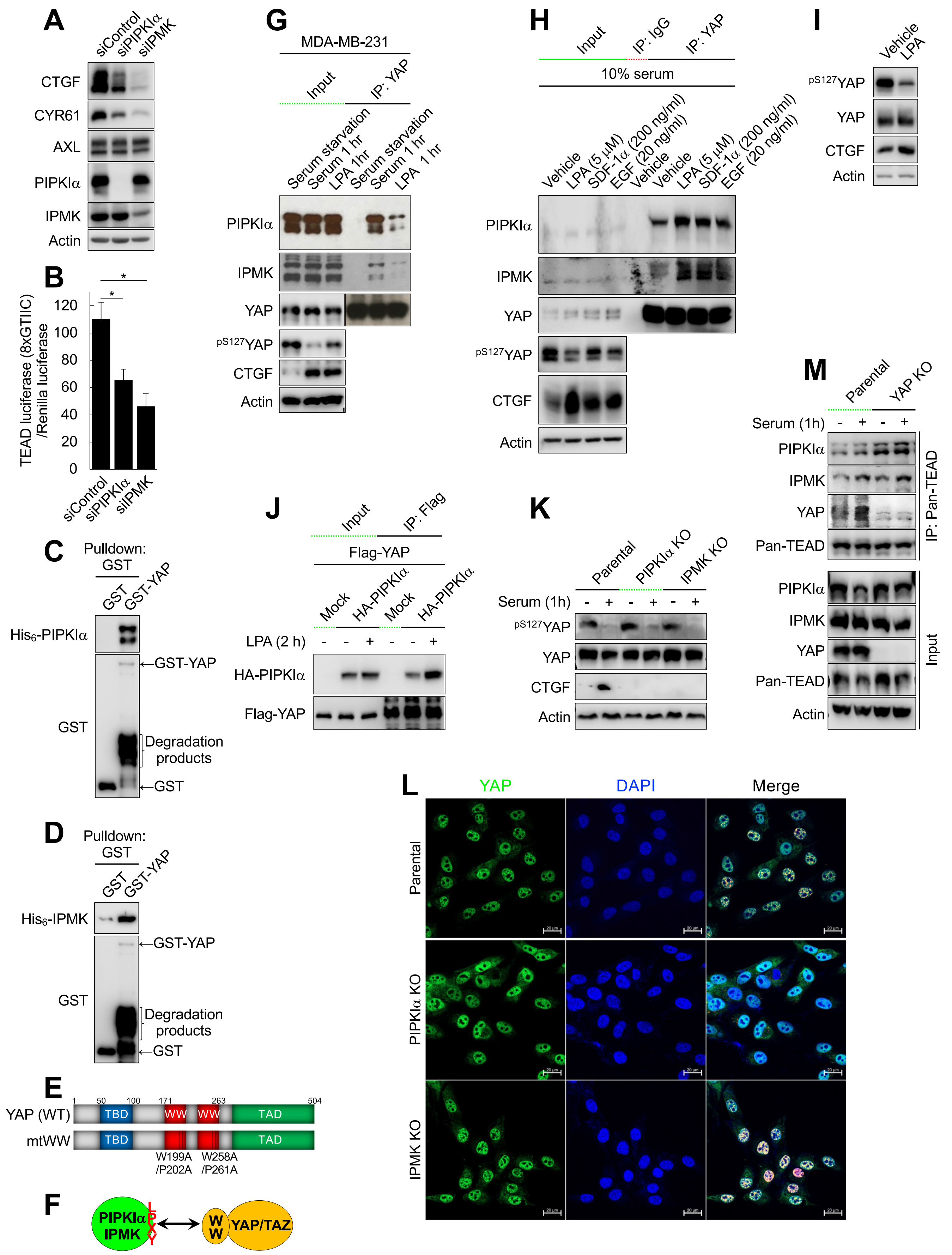
A
siControl
siPIPKIα
siIPMK
CTGF
CYR61
AXL
PIPKIα
IPMK
Actin
B
TEAD luciferase (8xGTIIC) /Renilla luciferase
siControl
siPIPKIα
siIPMK
C
Pulldown: GST
GST
GST-YAP
His6-PIPKIα
GST-YAP
GST
Degradation products
D
His6-IPMK
E
YAP (WT)
mtWW
TBD
WW
TAD
W199A /P202A
W258A /P261A
F
PIPKIα
IPMK
W
YAP/TAZ
G
MDA-MB-231
Input
IP: YAP
Serum starvation
Serum 1 hr
LPA 1hr
LPA 1 hr
YAP
pS127YAP
H
IP: IgG
10% serum
Vehicle
LPA (5 μM)
SDF-1α (200 ng/ml)
EGF (20 ng/ml)
I
LPA
J
IP: Flag
Flag-YAP
Mock
HA-PIPKIα
LPA (2 h)
Flag-YAP
K
Parental
PIPKIα KO
IPMK KO
Serum (1h)
L
YAP
DAPI
Merge
M
YAP KO
Pan-TEAD
IP: Pan-TEAD
Input

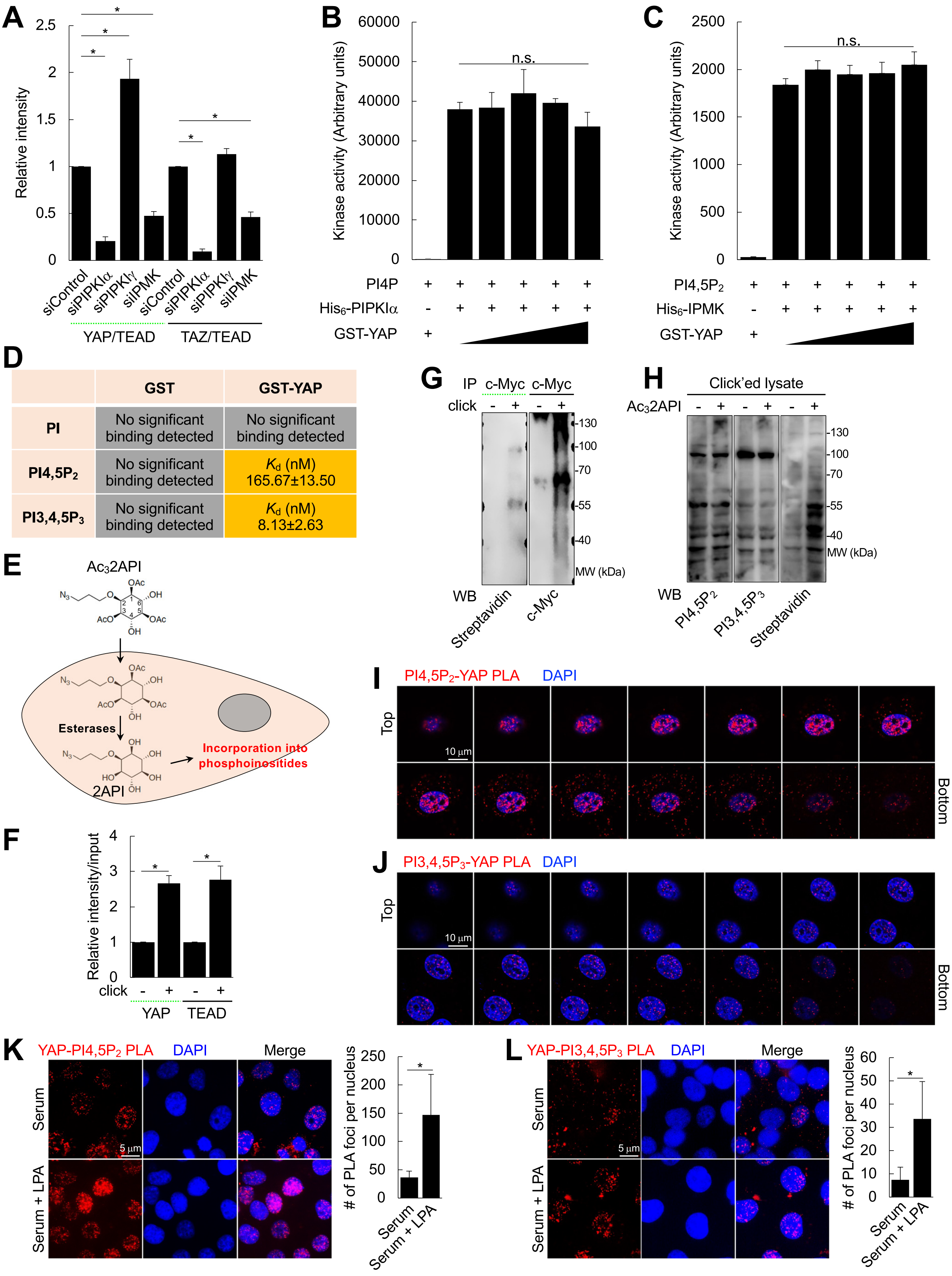
A
Relative intensity
siControl
siPIPKIα
siPIPKIγ
siPMK
siControl
siPIPKIα
siPIPKIγ
siPMK
YAP/TEAD
TAZ/TEAD
B
Kinase activity (Arbitrary units)
n.s.
PI4P
His6-PIPKIα
GST-YAP
C
Kinase activity (Arbitrary units)
n.s.
PI4,5P2
His6-IPMK
GST-YAP
D
GST
GST-YAP
PI
No significant binding detected
No significant binding detected
PI4,5P2
No significant binding detected
Kd (nM) 165.67±13.50
PI3,4,5P3
No significant binding detected
Kd (nM) 8.13±2.63
E
Ac32API
Esterases
2API
Incorporation into phosphoinositides
F
Relative intensity/input
click
YAP
TEAD
G
IP c-Myc c-Myc
click
MW (kDa)
WB
Streptavidin
c-Myc
H
Click'ed lysate
Ac32API
MW (kDa)
WB
PI4,5P2
PI3,4,5P3
Streptavidin
I
PI4,5P2-YAP PLA DAPI
Top
Bottom
10 μm
J
PI3,4,5P3-YAP PLA DAPI
Top
Bottom
10 μm
K
YAP-PI4,5P2 PLA
DAPI
Merge
Serum
Serum + LPA
5 μm
# of PLA foci per nucleus
L
YAP-PI3,4,5P3 PLA
DAPI
Merge
Serum
Serum + LPA
5 μm
# of PLA foci per nucleus

Figure EV2. YAP does not alter the kinase activity of PIPKIα or IPMK, and serum and LPA stimulate the associations of YAP with PI(4,5)P_2 and PI(3,4,5)P_3 in the nucleus.

(**A**) The intensity of immunoblots of Fig. 3A was quantified using ImageJ and the graph shows the mean ± s.d. of $n = 3$ independent experiments. *$P < 0.05$; **$P < 0.01$, and n.s.; not significant in Student's *t* test. (**B**, **C**) 0.05 μM His_6-tagged PIPKIα (**B**) and IPMK (**C**) were incubated with 0.2 μM diC8 PI(4)P or diC8 PI(4,5)P_2, respectively, in the absence or presence of various concentrations of GST-YAP (0.001, 0.01, 0.1, 1.0, and 10.0 μM). The activities of the kinases were measured using the ADP-Glo assay (Promega). The graphs show the mean±s.d. of $n = 3$ independent experiments. YAP did not alter the activity of either kinase. *$P < 0.05$; **$P < 0.01$, and n.s.; not significant in Student's *t* test. (**D**) Summary of GST alone or GST-YAP bindings to PI, PI(4,5)P_2, or PI(3,4,5)P_3 measured by MST. Raw data are available in Appendix Fig. S1. (**E**) A schematic representation of the molecular structure $Ac_3$2API and how it can become metabolically incorporated into phosphoinositides after removal of acetyl groups by esterase to produce azido-*myo*-inositol probe 2API. (**F**) The intensity of the immunoblots of Fig. 4B was quantified using ImageJ and the graph shows the mean ± s.d. of $n = 3$ independent experiments. *$P < 0.05$; **$P < 0.01$, and n.s.; not significant in Student's *t* test. (**G**) Starved MDA-MB-231 cells were fed with $Ac_3$2API for 24 h in the presence of 10% dialyzed serum. Cells were lysed and azide-tagged molecules were conjugated to biotin–alkyne through a click reaction. Endogenous c-Myc was immunoprecipitated and the associated complexes were analyzed by immunoblotting. Representative immunoblot images of $n = 2$ independent experiments are shown. (**H**) The clicked lysates were analyzed by anti-PI(4,5)P_2 or PI(3,4,5)P_3 antibodies. Biotinylated 2API was resolved by streptavidin. Many immunoblot bands overlapped with streptavidin signals. Representative immunoblot images of $n = 3$ independent experiments are shown. (**I**, **J**) Starved MDA-MB-231 cells were stimulated with 10% serum for 1 h. The images are z-stacks of PI(4,5)P_2-YAP PLA (**E**) and PI(3,4,5)P_3-YAP PLA (**F**) taken using a confocal microscope with each frame differing by 0.2 μm. DAPI was used to stain the nucleus. Representative images of $n = 3$ independent experiments are shown. Scale bar, 10 μm. (**K**, **L**) MDA-MB-231 cells grown in 10% serum were stimulated with 5 μM LPA for 90 min. Cells were fixed and the association of YAP with PI(4,5)P_2 (**G**) or PI(3,4,5)P_3 (**H**) was visualized by PLA. The images were obtained by widefield epifluorescence microscopy. The number of PLA puncta was counted from at least 10 cells and the graph shows the mean±s.d. of $n = 3$ independent experiments. DAPI staining was used to distinguish the nucleus from the cytoplasm. Treating the cells with LPA significantly increased the number of nuclear puncta. Scale bar, 10 μm. *$P < 0.05$; **$P < 0.01$, and n.s; not significant in Student's *t* test.

 © The Author(s)

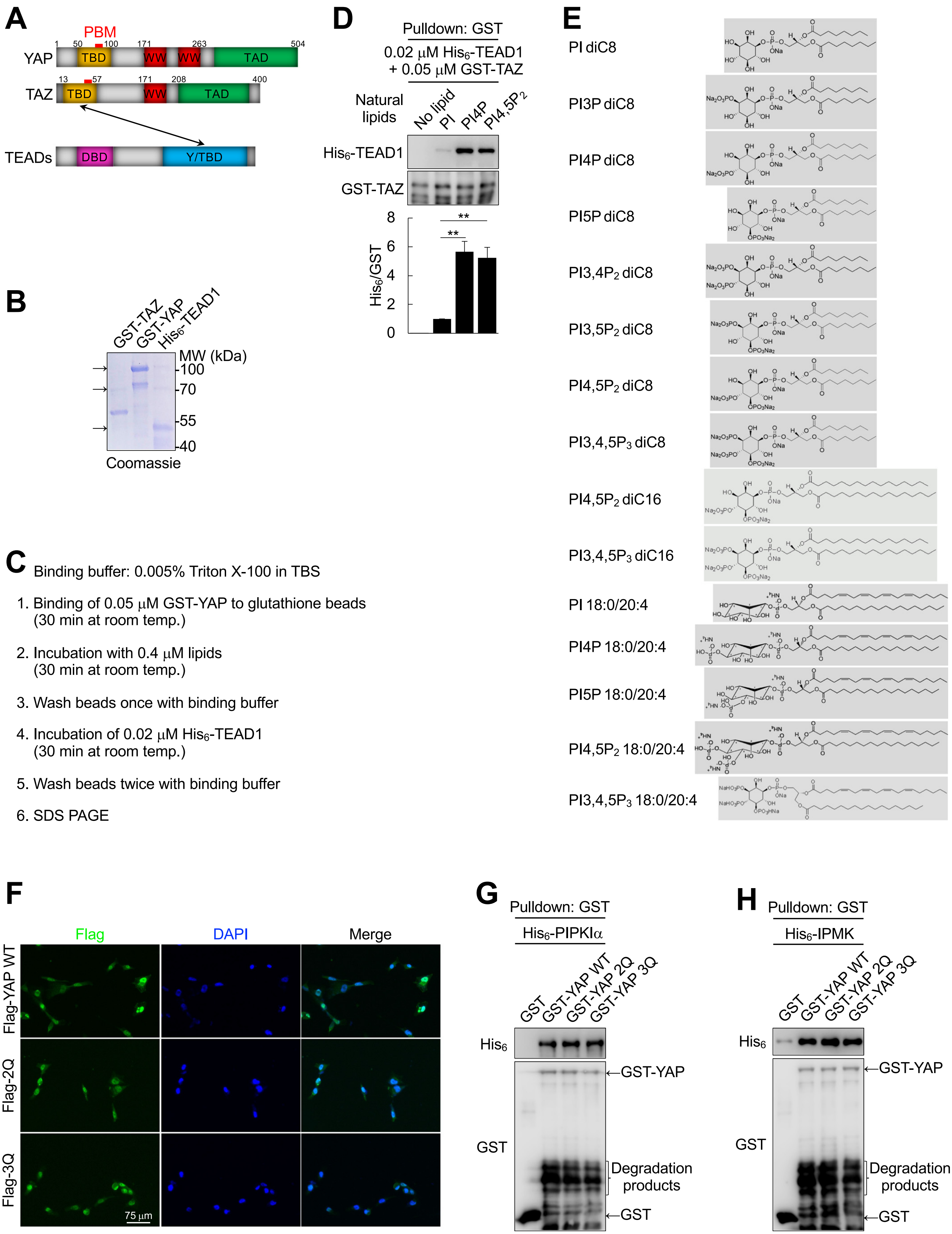
A
PBM
1 50 100 171 263 504
YAP TBD WW WW TAD
13 57 171 208 400
TAZ TBD WW TAD
TEADs DBD Y/TBD
B
GST-TAZ GST-YAP His6-TEAD1
MW (kDa)
100
70
55
40
Coomassie
C
Binding buffer: 0.005% Triton X-100 in TBS
1. Binding of 0.05 μM GST-YAP to glutathione beads (30 min at room temp.)
2. Incubation with 0.4 μM lipids (30 min at room temp.)
3. Wash beads once with binding buffer
4. Incubation of 0.02 μM His6-TEAD1 (30 min at room temp.)
5. Wash beads twice with binding buffer
6. SDS PAGE
D
Pulldown: GST
0.02 μM His6-TEAD1 + 0.05 μM GST-TAZ
Natural lipids
No lipid PI PI4P PI4,5P2
His6-TEAD1
GST-TAZ
His6/GST
8 6 4 2 0
**
**
E
PI diC8
PI3P diC8
PI4P diC8
PI5P diC8
PI3,4P2 diC8
PI3,5P2 diC8
PI4,5P2 diC8
PI3,4,5P3 diC8
PI4,5P2 diC16
PI3,4,5P3 diC16
PI 18:0/20:4
PI4P 18:0/20:4
PI5P 18:0/20:4
PI4,5P2 18:0/20:4
PI3,4,5P3 18:0/20:4
F
Flag DAPI Merge
Flag-YAP WT
Flag-2Q
Flag-3Q
75 μm
G
Pulldown: GST
His6-PIPKIα
GST GST-YAP WT GST-YAP 2Q GST-YAP 3Q
His6
GST
GST-YAP
Degradation products
GST
H
Pulldown: GST
His6-IPMK
GST GST-YAP WT GST-YAP 2Q GST-YAP 3Q
His6
GST
GST-YAP
Degradation products
GST

◀ **Figure EV3. The association of TAZ with TEAD1 is facilitated by phosphoinositides, and the binding of phosphoinositides does not alter the nuclear localization of YAP nor its binding to PIPKIα and IPMK.**

(**A**) The schematic diagrams show the polybasic motifs (PBM) located within the TEAD-binding domains (TBD) of YAP and TAZ that may mediate their binding to phosphoinositides. DBD, DNA-binding domain; TAD, transactivation domain; Y/TBD, YAP/TAZ-binding domain in the TEADs. (**B**) A Coomassie-stained gel of the three recombinant proteins used in the study is shown. The positions of the full-length proteins are indicated by arrows. (**C**) A detailed protocol used for the in vitro binding assays with lipids is presented. Note that the lipids and Triton X-100 were used at concentrations below their critical micelle concentrations. (**D**) 0.02 μM His_6-TEAD1 was incubated with 0.05 μM GST-TAZ in the absence or presence of the indicated lipids (0.4 μM). TAZ was pulled down with glutathione beads and the associated TEAD1 was analyzed by immunoblotting. The graph shows the mean ± s.d. of $n = 3$ independent experiments. *$P < 0.05$; **$P < 0.01$, and n.s.; not significant in Student's *t* test. (**E**) The chemical structures of the lipids used in the study are shown. (**F**) Flag-tagged WT YAP and the 2Q and 3Q mutants were transiently expressed in MDA-MB-231 cells. Cells were fixed and exogenous YAP proteins were visualized by immunostaining with an anti-Flag antibody. The images were obtained by widefield epifluorescence microscopy. DAPI staining was used to distinguish the nucleus from the cytoplasm. Representative immunostaining images of $n = 2$ independent experiments are shown. Scale bar, 75 μm. All the forms of YAP localize to the nucleus. (**G**, **H**) 0.1 μM GST alone, GST-WT, GST-2Q, and GST-3Q YAP recombinant proteins were incubated with 0.05 μM His_6-tagged recombinant PIPKIα (**B**) or IPMK (**C**). The YAP proteins were pulled down with glutathione beads and the associated PIPKIα and IPMK were analyzed with immunoblotting. Representative immunoblot images of $n = 3$ independent experiments are shown. Both PIPKIα and IPMK bound equally well to all the forms of YAP.

 © The Author(s)

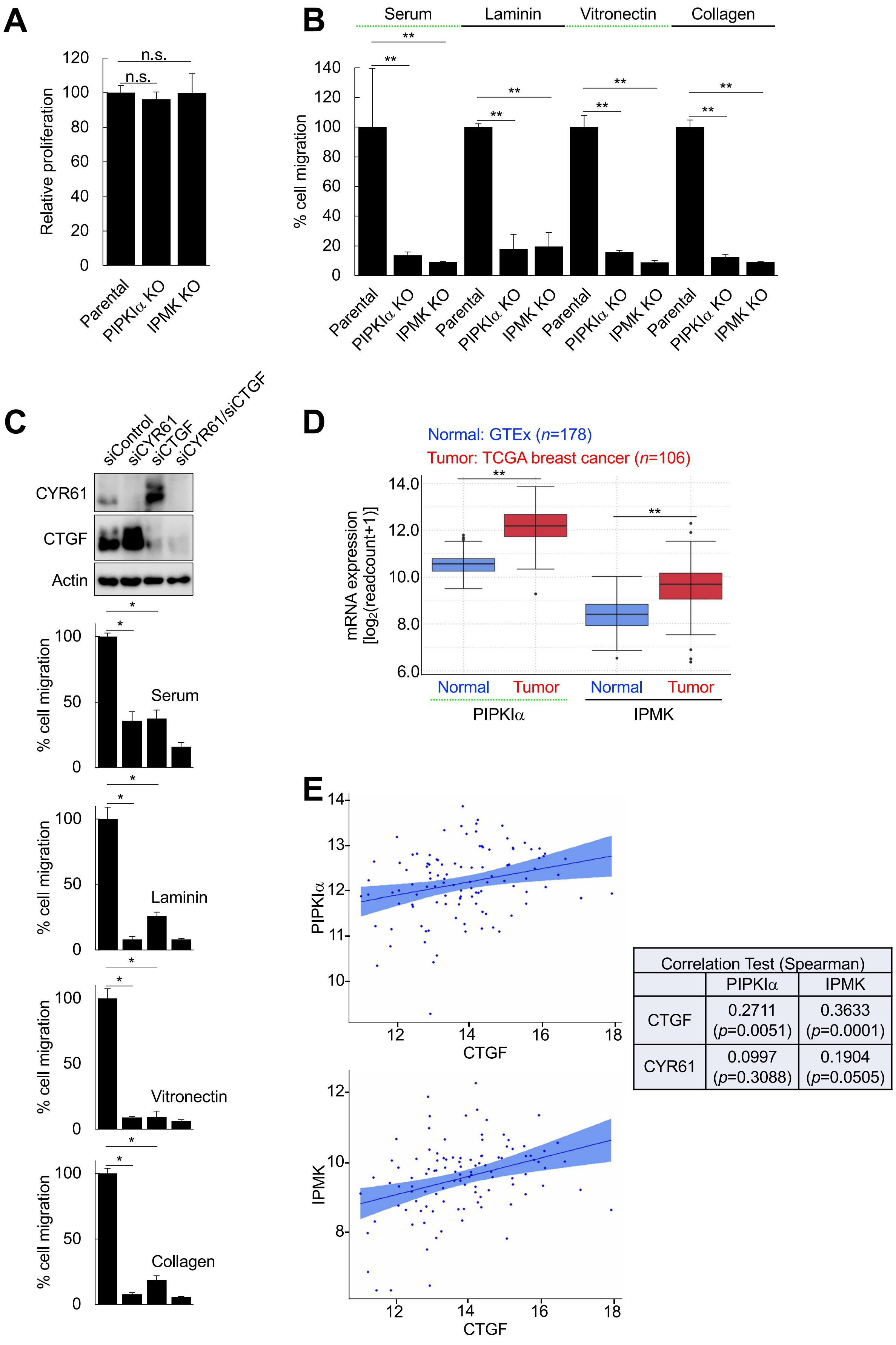
A
n.s.
n.s.
Relative proliferation
0
20
40
60
80
100
120
Parental
PIPKIα KO
IPMK KO
B
Serum
Laminin
Vitronectin
Collagen
**
% cell migration
0
20
40
60
80
100
120
140
Parental
PIPKIα KO
IPMK KO
C
siControl
siCYR61
siCTGF
siCYR61/siCTGF
CYR61
CTGF
Actin
*
% cell migration
0
50
100
Serum
Laminin
Vitronectin
Collagen
D
Normal: GTEx (n=178)
Tumor: TCGA breast cancer (n=106)
mRNA expression [log2(readcount+1)]
6.0
8.0
10.0
12.0
14.0
Normal
Tumor
PIPKIα
IPMK
E
PIPKIα
IPMK
CTGF
12
14
16
18
Correlation Test (Spearman)
PIPKIα
IPMK
CTGF
0.2711 (p=0.0051)
0.3633 (p=0.0001)
CYR61
0.0997 (p=0.3088)
0.1904 (p=0.0505)

Figure EV4. PIPKIα and IPMK are required for breast cancer cell motility and are overexpressed in breast tumors.

(**A**) The relative proliferation rates of MDA-MB-231 cells and cells with either PIPKIα or IPMK knocked out were measured using the Cell-Titer Glo assay (Promega). The graph shows the mean ± s.d. of $n = 3$ independent experiments. Knocking down either PIPKIα or IPMK did not affect the proliferation rate. *$P < 0.05$; **$P < 0.01$, and n.s.; not significant in Student's *t* test. (**B**) The migration of MDA-MB-231 cells and cells with either PIPKIα or IPMK knocked out towards 10% serum, 10 μg/ml laminin, 10 μg/ml vitronectin, or 25 μg/ml collagen IV was measured using Transwell inserts with 8.0 μm pores (Corning). The cells that migrated through the filter were visualized by DAPI and crystal violet staining and quantified by counting (Appendix Fig. S2A). The graph shows the mean ± s.d. of $n = 3$ independent experiments. In each case the migration of the KO cells was significantly lower than that of the parental cells. *$P < 0.05$; **$P < 0.01$, and n.s; not significant in Student's *t* test. (**C**) CTGF and CYR61 were knocked down singly or together in MDA-MB-231 cells. The extent of knockdown was determined by blotting. Cell migration induced by serum or several extracellular matrix components was measured. The graphs show the mean ± s.d. of $n = 3$ independent experiments. In each case, knocking down CTGF or CYR61 either alone or together impaired the migration rate. *$P < 0.05$; **$P < 0.01$, and n.s.; not significant in Student's *t* test. (**D**) RNA sequencing data of normal breast (GTEx) (Consortium, 2013) and malignant breast tumor (TCGA breast cancer) (Cancer Genome Atlas Research et al, 2013) tissues were used to compare relative mRNA expression levels of PIPKIα and IPMK. The expression of both PIPKIα and IPMK is significantly higher in breast cancer tissue. Center lines of boxplots are the median of each cohort data. Bounds of boxplot is the range from the first quartile to the third quartile. The lower whiskers of boxplots are distance between the first quartile and the first quartile minus 1.5 interquartile. The higher whiskers of boxplots are distance between the third quartile and the third quartile plus 1.5 interquartile. *$P < 0.05$; **$P < 0.01$, and n.s.; not significant in Wilcoxon rank-sum test. (**E**) Using the RNA sequencing data from the TCGA database of malignant breast tumors ($n = 106$), the correlation of PIPKIα and IPMK mRNA expression with that of CTGF and CYR61 was analyzed via Spearman method. The expression of CTGF is significantly correlated with the expression of PIPKIα and IPMK.

 © The Author(s)
